# In Silico Antiprotozoal Evaluation of 1,4-Naphthoquinone Derivatives against Chagas and Leishmaniasis Diseases Using QSAR, Molecular Docking, and ADME Approaches

**DOI:** 10.3390/ph15060687

**Published:** 2022-05-31

**Authors:** Lina S. Prieto Cárdenas, Karen A. Arias Soler, Diana L. Nossa González, Wilson E. Rozo Núñez, Agobardo Cárdenas-Chaparro, Pablo R. Duchowicz, Jovanny A. Gómez Castaño

**Affiliations:** 1 Grupo Química-Física Molecular y Modelamiento Computacional (QUIMOL), Facultad de Ciencias, Universidad Pedagógica y Tecnológica de Colombia (UPTC), Avenida Central del Norte, Tunja 050030, Colombia; lina.prieto02@uptc.edu.co (L.S.P.C.); karen.arias02@uptc.edu.co (K.A.A.S.); dlissethn@gmail.com (D.L.N.G.); wilson.rozo@uptc.edu.co (W.E.R.N.); agobardo.cardenas01@uptc.edu.co (A.C.-C.); 2 Instituto de Investigaciones Fisicoquímicas Teóricas y Aplicadas, (CONICET—Universidad Nacional de La Plata), Diagonal 113 y Calle 64, C.C. 16, Sucursal 4, La Plata 1900, Argentina; pabloducho@gmail.com

**Keywords:** chagas, leishmaniasis, naphthoquinones, antiprotozoal evaluation, QSAR, molecular docking, ADME

## Abstract

Chagas and leishmaniasis are two neglected diseases considered as public health problems worldwide, for which there is no effective, low-cost, and low-toxicity treatment for the host. Naphthoquinones are ligands with redox properties involved in oxidative biological processes with a wide variety of activities, including antiparasitic. In this work, in silico methods of quantitative structure–activity relationship (QSAR), molecular docking, and calculation of ADME (absorption, distribution, metabolism, and excretion) properties were used to evaluate naphthoquinone derivatives with unknown antiprotozoal activity. QSAR models were developed for predicting antiparasitic activity against *Trypanosoma cruzi*, *Leishmania amazonensis*, and *Leishmania infatum*, as well as the QSAR model for toxicity activity. Most of the evaluated ligands presented high antiparasitic activity. According to the docking results, the family of triazole derivatives presented the best affinity with the different macromolecular targets. The ADME results showed that most of the evaluated compounds present adequate conditions to be administered orally. Naphthoquinone derivatives show good biological activity results, depending on the substituents attached to the quinone ring, and perhaps the potential to be converted into drugs or starting molecules.

## 1. Introduction

Chagas and leishmaniasis are two parasitic infectious diseases endemic to Latin America, considered by the World Health Organization (WHO) as neglected tropical diseases, for which there is currently no effective, safe, and economical chemotherapy treatment. For chagas, the WHO estimates that between 7 and 8 million people are infected, with 12,000 deaths and 70 million people at risk of contracting it per year [1,2], while for leishmaniasis, there is an estimated figure of 12 million infected people, 1.6 million new cases each year, and 350 million people at risk of acquiring it [3,4,5]. In response to this problem, a growing number of investigations, mainly in Latin America, are being carried out to find new powerful anti-chagas and anti-leishmaniasis agents with low toxicity.

Chagas disease is a zoonosis caused by the protozoan *Trypanosoma cruzi*, which affects around 150 species of mammals, including humans, and is mainly vector-borne by hematophagous insects of the *Tritominidae* subfamily (popularly known as kissing bugs, bed bugs, and whistles, among other names). Since 1970, the treatment against this pathology has been based mainly on the antiparasitic drugs Nifurtimox (NFX) and Benznidazole (BNZ). These medicines have an effectiveness of 70% during the acute phase, where the prevailing parasitic form is blood trypomastigotes, and decreases to 20% in the chronic phase, where the predominant form corresponds to the intracellular phase of amastigotes [6,7]. It has been proven that NFX and BNZ have a high toxicity for mammalian cells, with serious side effects, such as peripheral neuropathies, anorexia, nausea, and vomiting, as well as neurological reactions such as anxiety and disorientation, among others [8]. On the other hand, leishmaniasis encompasses a group of infectious diseases caused by at least 20 species of the *Leishmania* genus, including *L. braziliensis*, *L. amazonensis*, *L. infantum*, *L. guyanensis*, *L. panamensis*, and *L. mexicana*, which are the species that affect humans. Its transmission is mainly through vectors via insect bites of the *Psychodidae* family. This disease presents three different clinical forms in humans, canines, and several wild vertebrates: cutaneous, mucocutaneous, and visceral [3]. Current treatment against leishmaniasis is based on the use of pentavalent antimonials, such as Miltefosine, Amphotericin B (AmB), Paromomycin, and Pentamidine; however, there are restrictions on the use of these drugs due to their side effects, which include hepatic, cardiac, and renal toxicity [5]. Additionally, they present limitations, such as high production cost and increased resistance by the parasite [9].

Naphthoquinones stand out among the most studied natural compounds and synthetic derivatives for their anti-chagas and anti-leishmaniasis (mainly against *L. infantum* and *L. amazonensis*) activity. β-lapachone derivatives have exhibited potential anticancer, antiviral, antiparasitic (including anti-chagas and anti-leishmanial activity), antimicrobial, anti-inflammatory, anti-obesity, antioxidant, and neuroprotective activity, while showing low levels of toxicity to normal cells [10]. Naphthoimidazoles derived from β-lapachone have been prepared, and their trypanocidal activity has been evaluated using electron microscopy, flow cytometry, and biochemical techniques, indicating that some compounds lead to an oxidative imbalance, which generates the production of ROS and the death of the parasite [10,11,12,13]. De Silva et al. carried out molecular docking studies on two cysteine proteases: cruzin and rhodesain, which are fundamental in the metabolism of the *T. cruzi* parasite [14]. This allowed for the identification of 14 naphthoquinone derivatives with potential antiprotozoal activity, which were synthesized and tested in vitro. On the other hand, 2,3-diphenyl-1,4-naphthoquinone (DPNQ) is considered a potential chemotherapeutic agent against *T. cruzi* due to its high trypanocidal activity in phenotypic screening and experimental murine infection by *T. cruzi*. Treatment with DPNQ in infected female mice promoted a halving of the parasite load, and ensured a 60% survival rate of the animal [15]. In 2021, Becerra et al. used pharmacophoric models based on different studies to design and subsequently synthesize nine phenolic derivatives, which were tested against *T. cruzi* strains, most of which showed good activity compared to BNZ, and the best prospects showed low toxicity [16]. Plumbagin is a plant-derived naphthoquinone metabolite (5-hydroxy-2-methyl-1,4-naphthaquinone) that inhibits trypanothione reductase, and has been validated as a drug which is responsible for promoting oxidative stress in *Leishmania* [9]. Similarly, naphtherocarpanquinone (LQB-118), which has been evaluated against cutaneous leishmaniasis, showed activity against intracellular amastigotes of *L. amazonensis*. It is proposed, through an anti-leishmanial model, that a good level of administration could counteract this clinical form of leishmaniasis [17]. The leishmanicidal activity of a series of substituted bis-2-hydroxy-1,4-naphthoquinones prepared from lawsone with aliphatic and aromatic aldehydes has also been evaluated [18]. Overall, four of the bis-lawsone analogs showed results similar to Pentamidine, but without cytotoxicity to host cells. Other studies demonstrated the high activity and low toxicity for host cells of derivatives of nor-α-lapachone and α-lapachone fused with triazole; despite them, however, resistance similar to that presented by the reference drug (Pentamidine) was observed [19]. Likewise, α-lapachone has shown a similar inhibition to sodium stibogluconate (Pentostam) [20]. Acetylisolapachol showed greater activity against *L. amazonensis* and does not exhibit a risk to the host [21].

Continuing with our search for novel structures with high antiparasitic activity and low toxicity [22], in this work, we have carried out an extensive in silico study of substituted derivatives of 1,4-naphthoquinone as potential anti-chagas and anti-leishmanial candidates. QSAR modeling of anti-*T. cruzi* in trypomastigotes, anti-*L. amazonensis*, *anti-L. infantum,* and toxicity has been carried out, based on the antiparasitic derivatives of naphthoquinone and other related structures reported in the literature. The best QSAR models were used to evaluate the predictive antiparasitic and toxicity activity of 68 1,4-naphthoquinone derivatives with hitherto unknown biological activity. This study was complemented by the evaluation of the 68 candidates against 5 macromolecular targets related to vital processes for the survival of these protozoa. In the case of *T. cruzi*, trypanothione reductase (*Tc*TR) and lanosterol α-demethylase (*Tc*LαD) proteins were selected. For the *Leishmania* genus in general, trypanothione reductase (*L*TR) protein was selected, while for the *L. amazonensis* and *L. infantum* strains, arginase (*La*A) and tyrosine aminotransferase (*Li*TA) were selected, respectively. Additionally, the ADME (absorption, distribution, metabolism, and excretion) properties of the 68 naphthoquinone derivatives were evaluated. The best anti-chagas and anti-leishmanial candidates derived from this in silico study are currently in the organic synthesis stage at our lab for their subsequent in vitro evaluation.

## 2. Results and Discussion

### 2.1. QSAR Modeling

In total, four QSAR models were performed based on in vitro reports of naphthoquinone derivatives and related structures with anti-*T. cruzi* (in trypomastigotes) [23,24,25,26,27,28,29,30,31], anti-*L. amazonensis* [18,32,33,34,35,36,37,38,39], and anti-*L. infantum* [40,41,42,43,44,45] activities, as well as toxicity [30,46,47,48,49,50,51] evaluation. For each QSAR study, the top seven models were generated, which are shown in Tables S1 and S2 in the Supplementary Materials. In turn, the best model (in bold) among the seven was selected, which was performed considering high homogeneity (fitting, R^2^) between the calibration and validation groups with the lowest root mean square error (RMSE), thereby avoiding over-adjusted models. For selecting the best model, the Ockham’s principle of parsimony was also considered, which states that if there are models with statistical parameters of equal quality, the simplest one should be selected [52].

#### 2.1.1. QSAR Model for Anti-chagas Activity

**Figure A1:**
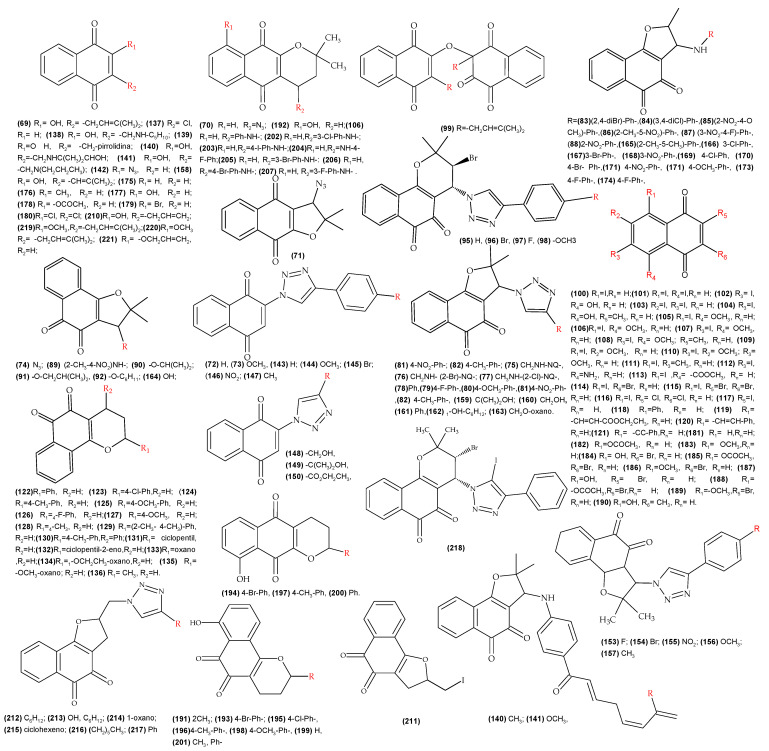
Molecules with reported biological activity (IC_50_) used for the development of the QSAR anti-*T. cruzi*.

Equation (1) shows the best QSAR model for the anti-chagas activity predicted as LogIC_50_, which was developed based on 153 derivatives of 1,2-naphthoquinone and 1,4-naphthoquinone (Figure A1) evaluating in vitro blood trypomastigotes of strain Y of *T. cruzi*. A description of the molecular descriptors that define this model is listed in Table 1.

(1) $${\mathrm{LogIC}}_{50}=4.5112+0.3637a_{1}+1.1416a_{2}-0.1003a_{3}+0.9440a_{4}+0.1929a_{5}-1.8425\times 10^{-4}a_{6}$$

The descriptors $a_{1}$ and $a_{2}$ are fingerprints calculated in Fragmentor [53], and refer to the presence of oxygen followed by an unsaturation; while the second was calculated in PaDeL [54], and belongs to the MACCS keys, a group of 166 free access fragments [55], which involves an oxygen–oxygen arrangement at a distance equal to three bonds and two intermediate atoms of any type. According to this QSAR model (Equation (1)), these molecular descriptors contribute negatively to the evaluated activity (i.e., they decrease the activity). The other descriptors of the model were calculated in the program QUBILS-MAS [56]. The descriptors $a_{3}$, $a_{4}$, and $a_{5}$ are calculated from the Kurtosis, which is a statistical invariant, while $a_{6}$ belongs to the Minkowski indicators. $a_{3}$ is a quadratic index, $a_{4}$ and $a_{5}$ are bilinear, and $a_{6}$ is linear. The descriptor $a_{3}$ is a global index that contributes to the biological activity. This is related to molar refractivity, which can sometimes be used to model London dispersion forces or attractive van der Waals interactions; these are factors related to the presence of strong interactions between a ligand and the active sites of a given macromolecular receptor. However, refractivity is the consequence of repulsive nonbonding interactions, and is highly dependent on the flexibility of the ligand [57]. The descriptor $a_{4}$ describes the electronegativity and polarizability of the fragment of carbon atoms in aliphatic chains, while $a_{5}$ is associated with the refractivity and charge of the subset of heteroatoms in the molecule [4]. Both descriptors contribute negatively to the anti-chagas activity. Considering the nature of descriptors $a_{4}$ and $a_{5}$, it is hypothesized that the anti-chagas activity could be related to the ability of the ligands to dock and inhibit essential macromolecules in the metabolism of *T. cruzi*. For its part, the descriptor $a_{6}$ provides a positive contribution to the biological activity, and is a function of the mass of aromatic carbon atoms, which suggests that the presence of aromatic systems improves activity.

Table S3 in the Supplementary Materials shows the intercorrelation relationship between each pair of descriptors included in the anti-chagas QSAR model, while Table S4 lists the values of each descriptor for each molecule. Table S5 shows the experimental values together with those predicted by the anti-chagas activity model as log IC_50_.

#### 2.1.2. QSAR Model for Anti-*L. amazonensis* Activity

**Figure A2:**
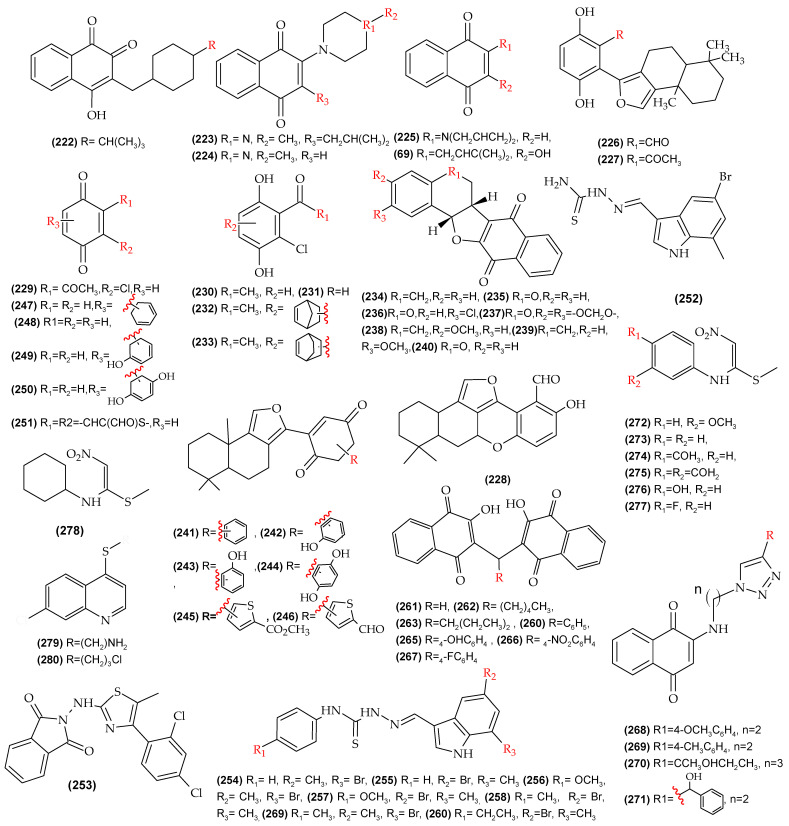
Molecules with reported biological activity used for the development of the anti-L. amazonensis QSAR model.

For the anti-*L. amazonensis* (cutaneous and mucocutaneous leishmaniasis) activity (expressed as LogIC_50_), the QSAR model with four descriptors was selected (Table S1), which is described according to Equation (2). This model was developed based on 60 ligands (Figure A2), with in vitro anti-leishmanial activity reported as IC_50_:

(2) $${\mathrm{LogIC}}_{50}=0.26-1.00b_{1}+0.24b_{2}+0.42b_{3}-0.05b_{4},$$

where $b_{1}$,$\;b_{2}$, $b_{3}$, and $b_{4}$ are described as shown in Table 2.

The $b_{1}$ descriptor refers to the Klekota–Roth fingerprint KRFP2, and it comprises part of six of the seven anti-*L. amazonensis* QSAR models considered (Table S1). This descriptor provides a measure of chemical similarity related to a bond between two carbon atoms, where each carbon atom has, as its substituents, a hydrogen atom and two nonhydrogen atoms [54,58]. On the other hand, $b_{2}$, $b_{3}$, and $b_{4}$ refer to 2D type descriptors. The minHBint9 ($b_{2}$) descriptor describes the topological state of the atom’s environment, such as the electronic interactions present in the atoms of the molecule at a topological distance of nine with each atom. According to Equation (2), this characteristic decreases the IC_50_, that is, the less topological distance there is between the atoms that make up the structure, the better its antiprotozoal activity [59]. The IC3 descriptor ($b_{3}$) is based on the three-order neighborhood symmetry. This means that molecules with lower symmetry will have a better predictive activity [60]. Finally, the descriptor MDEC-23 ($b_{4}$) refers to the information regarding the edge of the molecular distance among all secondary and tertiary carbons. The presence and value of $b_{4}$ enhance the anti-leishmaniasis activity predicted by the model, indicating that the presence of secondary and tertiary carbon atoms in these molecules is related to their biological activity [61,62,63].

#### 2.1.3. QSAR Model for anti-*L. infantum* Activity

**Figure A3:**
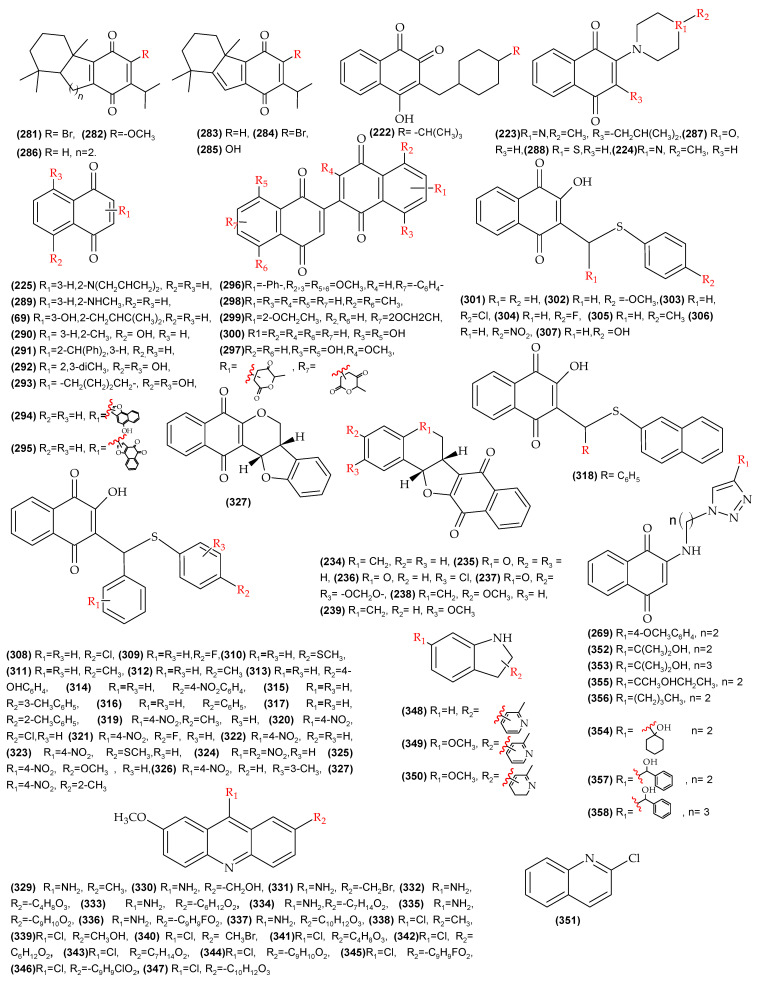
Molecules with reported biological activity used for the development of the anti-L. infantum QSAR model.

For predicting anti-*L. infantum* (visceral leishmaniasis) activity such as LogIC_50_, the model with six descriptors was selected (Table S1), which is represented according to Equation (3). The molecules used for constructing this QSAR model are shown in Figure A3:

(3) $${\mathrm{LogIC}}_{50}=-1.51+6.83c_{1}+0.29c_{2}-3.88\times 10^{-6}c_{3}+0.04c_{4}-3.72c_{5}-3.68\times 10^{-6}c_{6}$$

where $c_{n}\left(n=1\;\mathrm{to}\;6\right)$ are 2D descriptors described as shown in Table 3.

The MATS3c ($c_{1}$) descriptor refers to Moran’s autocorrelation, which expresses the partial charge values of atoms separated by three distances, that is, it estimates the correlation of charges divided into three bonds [64,65]. The descriptor nHBint7 ($c_{2}$) counts resistance E-state descriptors for hydrogen bonds with a path length of seven. The $c_{3}$–$c_{6}$ descriptors are part of the QuBiLS-MAS program [66]. The $c_{3}$ descriptor refers to the partition algorithm, which is used to calculate estimates of most neutral organic compounds that have C, H, O, N, S, Se, P, B, Si, and halogen atoms. This descriptor is based on AlogP, which can also estimate local hydrophobicity, visualize molecular hydrophobicity maps, and evaluate hydrophobic interactions when protein–ligand complexes are formed [67,68]. The $c_{4}$ descriptor considers bilinear indices and aliphatic chain carbon atoms, and correlates the topological area of the polar surface and the van der Waals volume. The polar surface area (PSA) is a molecular descriptor widely used in the study of drug transport properties, related to the penetration of the blood–brain barrier and its intestinal absorption. Additionally, the descriptor $c_{4}$ alludes to the sum of the contributions of polar atoms such as oxygen, nitrogen, and hydrogen to the molecular surface area [69,70]. The descriptor $c_{5}$ correlates the carbon atoms of aliphatic chains with the van der Waals volume and charge. Molecular volume is defined as a measure of the space around electron-filled atomic nuclei, and is geometrically interpreted as the combined volume of the superimposed spheres centered on the nuclei, similar to a space-filling molecular model [71,72]. Finally, the descriptor $c_{6}$ presents a nonstochastic matrix of order 15; in this case, the descriptor considers the heteroatoms different from C and H, correlating them with the partition algorithm and estimating the different atoms. This descriptor includes most of the zwitterionic compounds that have amine, carboxylic acids, and ammonium halide salts. The $c_{6}$ descriptor is also based on an intrinsically atomistic model, which is useful for drug design, since it makes estimates of the local or general hydrophobicity of a molecule [66,68].

Table S7 in the Supporting Materials shows the intercorrelation relationship between each pair of descriptors included in the anti-leishmanial QSAR models (Equations (2) and (3)), while Table S8 (*L. amazonensis*) and Table S7 (*L. infantum*) show the values of each descriptor calculated for each molecule used in the development of these QSAR models. Tables S10 and S11 list the experimental and model-predicted values of anti-leishmanial activity as log IC_50_.

#### 2.1.4. QSAR Model for Toxicity

**Figure A4:**
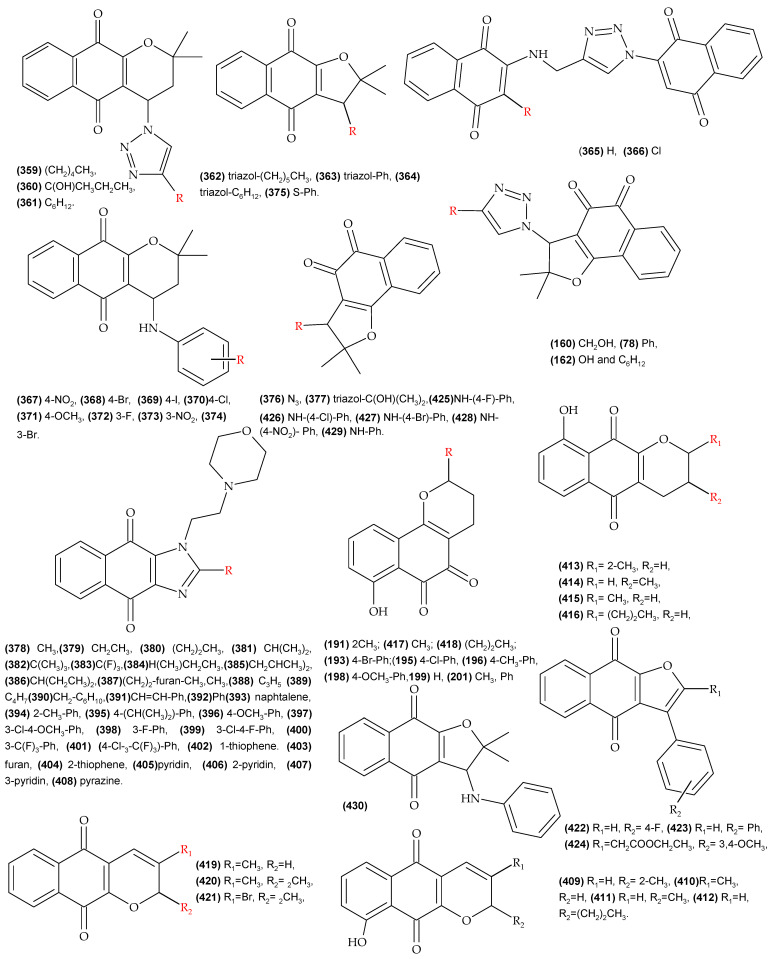
Molecules with reported toxicity in mouse fibroblasts (L929) used for the development of the QSAR model of toxicity.

From the QSAR study for toxicity (expressed as LogIC_50_), a model with five descriptors was selected (Table S2), which is represented in Equation (4). This model was developed based on 76 naphthoquinone derivatives, as shown in Figure A4.

(4) $${\mathrm{LogIC}}_{50}=0.37+0.11d_{1}-0.21d_{2}+0.36d_{3}-0.05d_{4}-0.17d_{5},$$

where $d_{n}\left(n=1\;\mathrm{to}\;5\right)$ are 2D descriptors described as shown in Table 4.

The toxicity model descriptors were calculated using the QUBILS-MAS program [66]. The descriptors $d_{1}$, $d_{2}$, $d_{4}$, and $d_{5}$ are calculated from the Kurtosis, which is a statistical invariant of distribution. The $d_{1}$ descriptor correlates with physicochemical properties, polarized topological surface area, and refractivity. As indicated above, the topological polar surface area of a molecule depends on the sum of the surface area of polar atoms, such as oxygen, nitrogen, and hydrogen, and facilitates the ability of a molecule to penetrate cells. According to this, the greater the value of the polar topological surface in a molecule, the greater its probability of being transported [73]. Meanwhile, refractivity is a measure of the volume occupied by an atom or a group of atoms [69]. These last two properties allow for a theoretical prediction of the pharmacological potential of a molecule in a biological environment [74]. The descriptor $d_{2}\;$correlates the polarization with the charge of aromatic carbon atoms because it facilitates the distortion of the atomic or molecular charge in electromagnetic fields. This descriptor refers to an electronic parameter, which impacts chemical–biological interactions [75]. The descriptor $d_{3}$ correlates the polar surface area with the polarization of the carbon atoms of the aromatic moiety and the heteroatoms attached to this moiety. The $d_{4}\;$descriptor is associated with the partition algorithm, heteroatoms, and electronegativity. This descriptor is based on the tendency of a heteroatom or functional group to attract electrons and estimate the local or general hydrophobicity of a molecule [67,68,69,70,71,72,73,74,76,77]. Finally, the descriptor $d_{5}\;$refers to the charge and the carbon atoms in the aliphatic chains.

Table S13 in Supporting Materials shows the intercorrelation relationship between each pair of descriptors included in the QSAR model of toxicity, while Table S15 shows the experimental and predicted values in log IC_50_ used for constructing of the QSAR model of toxicity.

#### 2.1.5. Validation of QSAR Models

Table 5 contains a compilation of the statistical parameters used for the internal and external validation of the four QSAR models developed. From Y-randomization, it was found that *RMSE_cal_* < *RMSE_rand_*, thus indicating that all QSAR models are robust due to the absence of random correlation [78] Internal validation by the Leave-One-Out (LOO) method yielded a value of the squared correlation coefficient ($R_{loo}^{2}$) ≥ 0.5 [78,79], which ensures the statistical stability of each model. In addition to presenting good internal validation parameters, all of the QSAR models are predictive, since they meet the following requirements: the slopes *k* and *k’* of the plots of observed and predicted values are in the range 0.85–1.15 (with *k* corresponding to the case when the predicted values are plotted on the x-axis and the experimental values on the y-axis, while *k’* is the inverse graph). The CCC statistically evaluates the models, this parameter verifies the difference between the experimental and predicted values. The squared correlation coefficients have values greater than 0.5, which validate the models [80].

**Figure 1:**
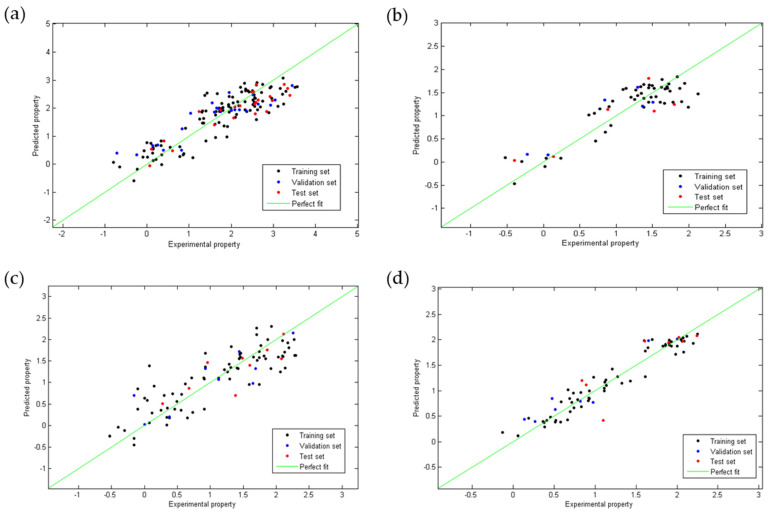
Linear correlation plots between the experimental and the predicted values obtained using the QSAR Equations (1) (**a**), (2) (**b**), (3) (**c**) and (4) (**d**).

Figure 1 shows the dispersion diagram of the experimental values of anti-chagas and anti-*Leishmania* (*L. amazonensis* and *L. infantum*) activity and toxicity expressed as *Log*(IC_50_) as a function of the values predicted by each model. In all cases, it is observed how points adopt a linear trend around the line of perfect fit (in green), which confirms a multivariate linear relationship.

**Figure 2:**
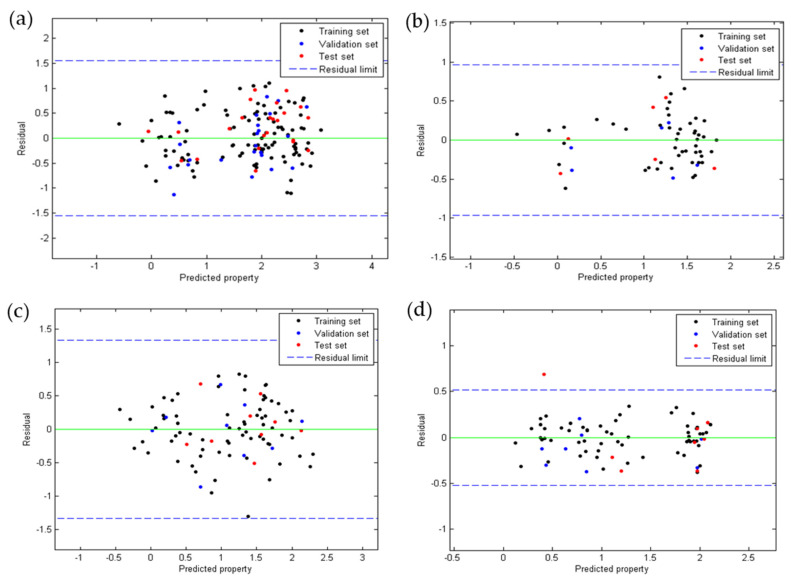
Dispersion of residues according to QSAR Equations (1) (**a**), (2) (**b**), (3) (**c**), and (4) (**d**).

Figure 2 shows the plots of the residuals for the four QSAR models developed. It was observed that for anti-chagas (Figure 2a) and anti-leishmaniasis (Figure 2b,c) activity, no residual is greater than three times the standard deviation (3S) of the model (outliers), while the toxicity model (Figure 2d) presented one outlier of the test group. In all QSAR models, the points follow a random distribution around the line at y=0, which suggests that these properties are modeled using of multiple linear regression.

**Figure 3:**
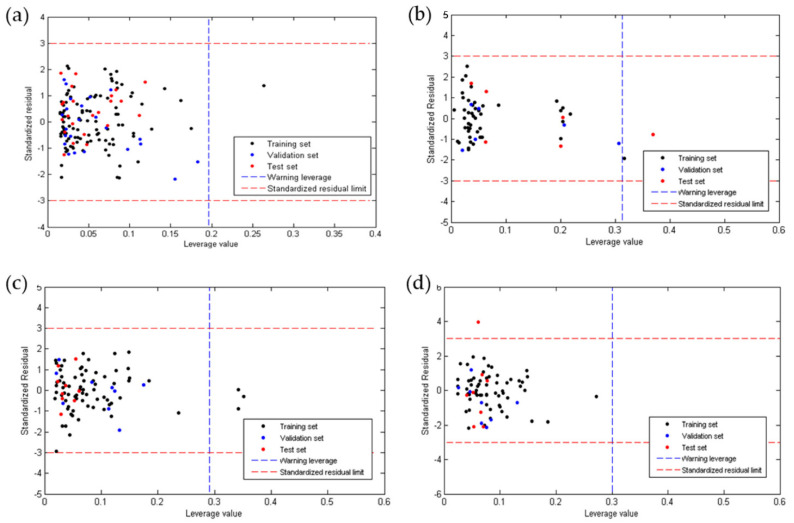
*h^lim^* values for (**a**) anti-chagas (Table S5); (**b**) anti-*L. amazonensis* (Table S10); (**c**) anti-*L. infantum* (Table S11) activity, and (**d**) toxicity (Table S15).

Due to the low diversity of molecules considered in the development of the QSAR models, the acceptable predictions are restricted to structural analogs derived from naphthoquinone, whose influence value is less than the critical influence value (h*) in each model (Figure 3). As shown in Figure 3a,c, for anti-chagas and anti-*L. infantum* activity, all molecules of the validation group and of the test group are within the domain of applicability, and molecules of the calibration group are considered outside the value of h*, a fact that reinforces the predictive capacity of these models. For the anti-*L. amazonensis* activity model, Figure 3b shows how a molecule of the test group is rejected, thus demonstrating its ability to reject molecules with large differences. For its part, the toxicity model shows that all molecules considered for its development and validation are within the domain of applicability.

#### 2.1.6. Molecular Design and Applicability of QSAR Models

Both *ortho*-naphthoquinone (1,2-substituted) and *para*-naphthoquinone (1,4-substituted) are recognized as highly active cores due to the synergy between its acid base and oxidation reduction properties [81], whose derivatives have exhibited several tunable antiparasitic effects according to the substitutions made in their fused rings [82]. As can be verified from the structural compilation used for constructing our QSAR antiprotozoal models (Figure A1, Figure A2 and Figure A3 in Appendix A), a high in vitro anti-chagas or anti-leishmanial effect is achieved when substitutions with heterocyclic, aromatic, or aliphatic groups are made at the 2 and 3 positions, for the case of the *para*-naphthoquinone, or at the 3 and 4 positions, for the case of the *ortho*-naphthoquinone nucleus.

**Figure 4:**
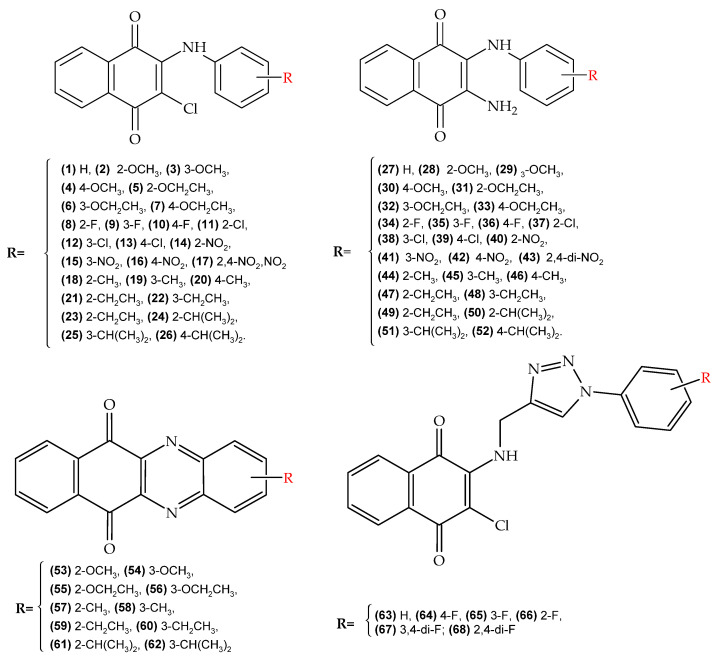
Molecular design of 1,4-naphthoquinone derivatives with potential antiparasitic activity (chagas and leishmaniasis). (**top-left**) (NQ–Chlorine family), (**top-right**) (NQ–Amine family), (**bottom-left**) (NQ–Phenazine), and (**bottom-right**) (NQ–Triazole family).

The molecular design of our selection group was based on the following principles: (i) restriction to only derivatives of the 1,4-naphthoquinone nucleus at positions 2 and 3; (ii) use of open-chain and heterocyclic substituents at these positions; (iii) use of nitrogenous derivatives in position 2; (iv) variation in position 3 with electro-withdrawing and electro-donor groups; and (v) substitution in the amino nitrogen, located in position 2, with both highly functionalized aromatic and heterocycle rings. Due to this strategy, four families of 1,4-naphthoquinone derivatives (Figure 4) were selected for in silico evaluation: (a) 2-chloro-3-arylamino family (NQ–Chlorine); (b) 2-amino-3-arylamino family (NQ–Amine); (c) 2-amino-3-triazolamino family (NQ–Triazole); and (d) phenazine family (NQ–Phenazine). In particular, the use of triazoles and phenazines as substituents was established based on the high intrinsic activity of these groups, as well as their enhancing effect when incorporated into other active nucleus [23,82,83,84].

QSAR models established according to Equations (1)–(4) were used to predict anti-chagas (IC_50_ in trypomastigotes), anti-*L. amazonensis* (cutaneous and mucocutaneous leishmaniasis), and anti-*L. infantum* (visceral leishmaniasis) activity, as well as toxicity, of the 68 derivatives of 1,4-naphthoquinone shown in Figure 4, which are structures that do not present experimental anti-chagas or anti-leishmanial activity (in vitro or in vivo) reported so far.

**Figure 5:**
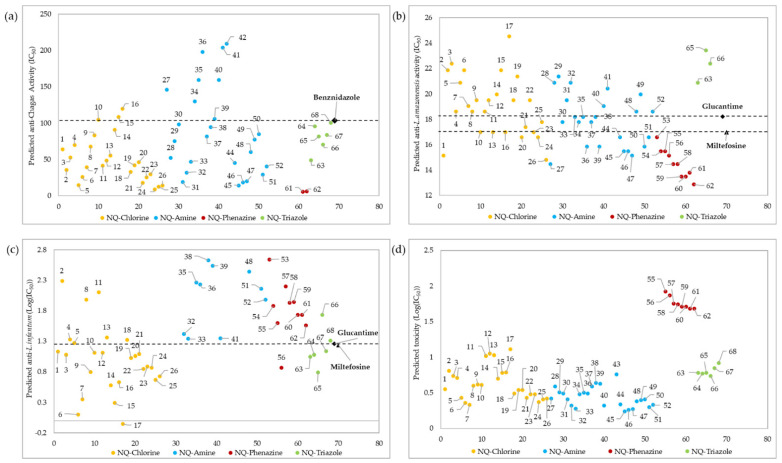
Prediction of anti-chagas (IC_50_) (Table S6) (**a**), anti-*L. amazonensis* (IC_50_) (**b**), and anti-*L. infantum* (LogIC_50_) (Table S12) (**c**) activity, as well as toxicity (LogIC_50_) (Table S16) (**d**), for the 68 1,4-naphthoquinone derivatives (*x*-axis) evaluated using QSAR Equations (1)–(4), respectively. Only structures with reliable activity, as determined by the influence value (h*), are presented. The predicted values for anti-*L. infantum* and toxicity are presented in LogIC_50_ because of the wide dispersion of the data.

Figure 5a shows the prediction values of anti-chagas activity (IC_50_) for the 68 1,4-naphthoquinone derivatives evaluated using QSAR Equation (1). As a result, 47 (69%) of the 68 evaluated molecules had a better predicted IC_50_ than the experimental value of the reference drug BNZ. Overall, 10 of these derivatives (structures 17, 43, 53–60) were not within the applicability domain of the model, so their predicted activity should be considered unreliable. High anti-chagasic activity was found for the NQ–Phenazine derivatives substituted with the isopropyl group (structures 61 and 62), as well as for the NQ–Chlorine and NQ–Amine derivatives containing the same substituent (structures 24–26 and 51–52, respectively). Additionally, the derivatives coupled to the triazole ring (NQ–Triazole family) show a slightly better predicted activity than BNZ. According to this QSAR model, those amino derivatives with electron-withdrawing substituents (nitro and fluorine, structures 34–36 and 40–42, respectively) present the less parasitic activity toward *T. cruzi* trypomastigotes.

The predicted IC_50_ values of anti-*Leishmania* activity (*L. amazonensis* and *L. infantum*) for the 68 naphthoquinone derivatives evaluated with QSAR Equations (2) and (3) are presented in Figure 5b,c respectively. According to the influence value (h*), 63 (93%) of the 68 naphthoquinone derivatives showed reliable anti-*Leishmania* activity, and five molecules (42, 43, 64, 67 and 68) did not show reliable activity. It was found that all molecules belonging to NQ–Phenazine family, as well as some derivatives of the NQ–Chloro (structures 1, 20, 24, and 26), and NQ–Amine families (structures 27, 36, 39, 44–47, 50–51), presented a better anti-*L. amazonensis* effect than the reference drugs Miltefosine and Glucantime (see Figure 5b), unlike the all of NQ–Triazole derivatives that had lower results than the reference drugs. According to Equation (3), 51 derivatives presented a reliable predictive anti-*L. infantum* activity, while the other 17 molecules were not found within the influence parameter (0.24). Among the molecules that presented a reliable activity, the best activity was that of molecule 6 together with several other NQ–Chloro derivatives (structures 1, 3, 6–7, 9–10, 12, 14–17, and 19–26), one NQ–Phenazine derivative (structure 56), and four of the NQ–Triazole derivatives (structures 63–65 and 67), all of which presented better activity than the reference drug. All of the NQ–Amine derivatives presented lower anti-*L. infantum* activity than the two reference drugs.

The toxicity values (LogIC_50_) predicted by QSAR Equation (4) for the 68 naphthoquinone derivatives are shown in Figure 5d. A total of 64 molecules presented a reliable predicted activity, while 4 of them (structures 41–42, 53 and 54) were outside of the influence parameter (h*). Among the 68 molecules evaluated, all of the structures belonging to the NQ–Phenazine family presented the lowest toxicity values.

### 2.2. Molecular Docking

Ligand–protein docking simulations were carried out to determine the most effective binding mode of each of the 68 1,4-naphthoquinone derivatives within the catalytic sites of the 5 chosen molecular targets (*Tc*TR, *Tc*LαD, *L*TR, *La*R, and *Li*TA). These macromolecular receptors were selected due to their relevance in the processes of survival, metabolism, reproduction, and proliferation of the parasites *T. cruzi*, *L. amazonensis,* and *L. infantum*. Conformational flexibility was allowed in all rotational bonds of the ligand, while the protein was used as a rigid structure. The best poses were selected according to the MVD scoring function, which helped to elucidate the electronic and structural aspects of the binding mode of the ligands in the active site of each protein.

#### 2.2.1. Docking in Trypanothione Reductase and Lanosterol α-Demethylase Proteins of *T. Cruzi*

**Figure 6:**
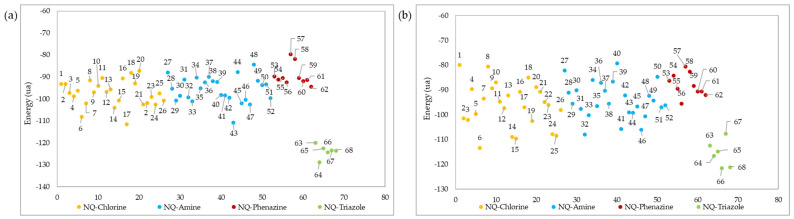
Ligand–protein docking energies of 1,4-naphthoquinone derivatives against (**a**) *Tc*TR and (**b**) *Tc*LαD receptors.

For the *Tc*TR protein a 496 Å^3^ cavity was used, while for the *Tc*LαD protein, a 481 Å^3^ cavity was used (Figure S1). The results of the ligand–protein coupling are shown in Figure 6a,b, where the 68 1,4-naphthoquinone derivatives show a good interaction (from −128.75 to −79.68 kcal/mol) with the two selected molecular targets. In this study, as the interaction energy decreases, the affinity of the compounds with the enzyme improves.

**Figure 7:**
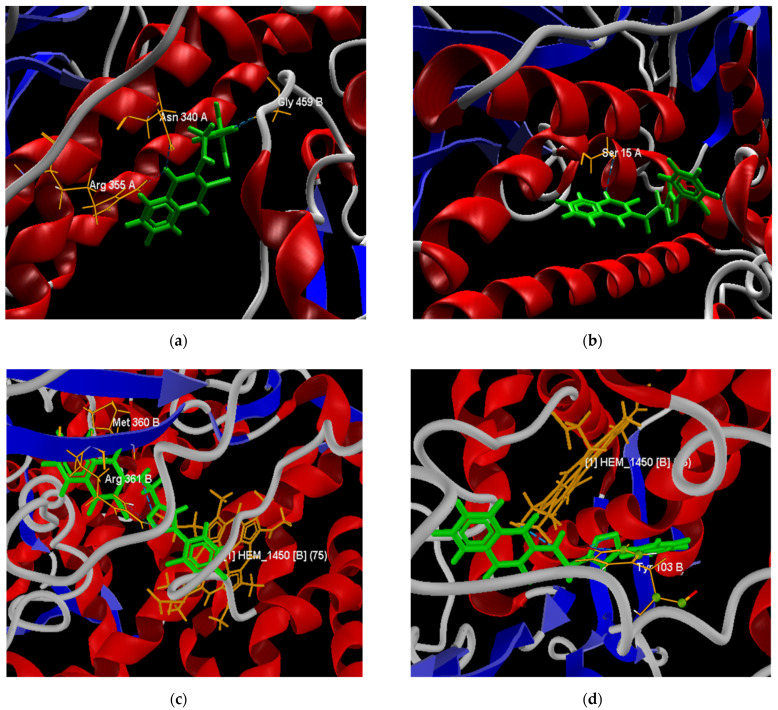
Best poses of derivatives 64 (**a**) and 66 (**b**) in the active site of the *Tc*TR protein. Best poses of derivatives 66 (**c**) and 68 (**d**) in the active site of the *Tc*LαD protein.

As shown in Figure 6a, the triazole-fused naphthoquinone derivatives had better interaction energy (from −128.75 to −120.03 kcal/mol) with the active site of the *Tc*TR. Among these, NQ–Triazole structure 64 had the best affinity, presenting 4 hydrogen bonds: 1 with the Asn 340 A residue, another with the Arg 355 A residue, and 1 with the Gly 459 B residue (Figure 7a), with the latter 2 not present in its triazole analogs; as well as steric interactions with the amino acids His 461, Thr 335, Ile 339, Glu 466, Glu 19, Pro 336, and Ser 470. Chacón et al. [85] also reported that the hydrogen bond interaction with the Gly 459 residue provided the best affinity energy to the quinoxaline derivative of the group they evaluated; and reported for this compound the same hydrophobic interactions. Similarly, the natural substrate co-crystallized in the active site of the *Tc*TR protein presented interactions with residues Glu 19 A, Gly 459 B, Pro 336 A, Ile 339 A, and His 461 B [86]. NQ–Triazole 66 presented an energy close to that of molecule 64, and in addition to the same hydrophobic interactions, it presented unions with the amino acids Leu 18 A, Tyr 111 A, and Val 54, which are also part of the *Tc*TR-trypanothione union (Figure 7b). The only hydrogen bond that this derivative presents together with the other NQ–triazole molecules is with the Ser 15 A residue, which is a solvent-mediated hydrogen bond interaction [86]. Note that this interaction occurs only in this series of compounds. Table S17 in the Supporting Materials shows the interaction energy of each ligand, as well as the hydrogen bonds with the different amino acid residues of the active site of the *Tc*TR and *Tc*LαD proteins.

NQ–Chlorine derivatives 6 (−108.09 kcal/mol), and 17 (−111.59 kcal/mol), as well as NQ–Amine derivative 43 (−110.87 kcal/mol), also presented high ligand–protein interaction energies. Although these compounds have hydrogen bonds in the *Tc*TR active site (Figure 7c), the strength of these hydrogen bonds is comparable to those in compounds that have the least favored interaction energies, i.e., NQ–Phenazine structures 57 (−79.68 kcal/mol) and 58 (−81.93 kcal/mol). From the above, it follows that the compounds 6, 17, and 43 achieve their highest affinity and stability through other types of interactions.

The molecular docking evaluation against the *Tc*LαD protein showed that molecule 66 was again the ligand with the best affinity (−121.60 kcal/mol), followed by molecule 68 (−121.18 kcal/mol). Both structures belong to the family of triazoles substituted with fluorine atoms in the terminal aromatic ring, in this case, in the *meta* and *ortho*-*para* positions, respectively. Derivative 66 presents three hydrogen bonds between the three nitrogen atoms of the triazole ring with the donor oxygen atom of the Tyr 103 (B) residue; this type of interaction was also found in the binding site of Fluconazole and Posaconazole with a single hydrogen bond [87]. Ligand 66 also exhibits hydrophobic interactions with residues Tyr 116 (B), Ala 291 (B), Ala 287 (B), Met 460 (B), Phe 290 (B), Met 106 (B), and Met 40 (B). A capture the pose of compound 66 at the active site of the *Tc*LαD target is presented in Figure 7c. Structure 68 presents two hydrogen bonds, the strongest with the iron protoporphyrin IX enzymatic cofactor (HEM_1450 or Heme) co-crystallized in the amino acid chain B, and the other with the Tyr 103 residue (B) (Figure 7d), which is consistent with the interactions reported for this receptor with Fluconazole and Posaconazole [87]. The steric interactions that stabilize ligand 68, also present in the two azoles mentioned [87], occur with residues Ala 291 (B), Ala 287 (B), Tyr 116 (B), and Phe 110 (B). Cardoso et al. reported a hydrogen bond between their furan–naphthoquinones with the Tyr 116 residue in the binding site of this protein [88]; however, for molecules 66 and 68, the interactions with this residue are of the steric type. These same authors report cation-π interactions with the Fe of the Heme group, which were not observed in any 1,4-naphthoquinone derivative tested here.

Other structures that presented favorable interaction energies with the active site of the *Tc*LαD protein were the NQ–Amine derivatives 32 (−107.97 kcal/mol), 41 (−105.71 kcal/mol), and 46 (−106.12 kcal/mol), and the NQ–Chlorine derivatives 6 (−113.43 kcal/mol), 14 (−108.92 kcal/mol), 15 (−109.63 kcal/mol), 24 (−107.88 kcal/mol), and 25 (−108.50 kcal/mol). Among these, the lowest energy was found for the derivative substituted with an ethoxide group in the *meta* position of the phenylamino aromatic ring (structure 6), which did not present hydrogen bond interactions. The other 30 molecules did not present formation of hydrogen bonds with any amino acid residue of the active site.

#### 2.2.2. Docking in Trypanothione Reductase, Arginase, and Aminotransferase Proteins of *Leismania* Genus

The docking evaluation of the 68 1,4-naphptoquinone derivatives in the catalytic sites of the proteins trypanothione reductase (*L*Tr), arginase (*La*A), and aminotransfera (*Li*AT) was carried out using cavities of 705, 305, and 1171 Å^3^, respectively (Figure S2).

**Figure 8:**
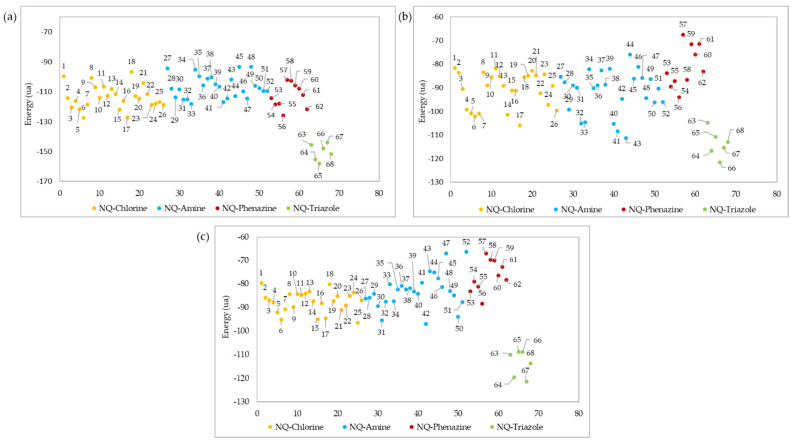
Ligand–protein docking energies of 1,4-naphthoquinone derivatives against (**a**) *L*Tr (Table S18), (**b**) *La*A (Table S19), and (**c**) *Li*AT (Table S20) receptors.

The ligand–protein interaction energies for the best pose of each of the 68 derivatives evaluated are shown in Figure 8. In all cases, the most effective interactions against the three macromolecular receptors of the *Leishmania* genus were obtained for derivatives belonging to the NQ–triazoles. Molecules 65, 66, and 67 showed the best interaction energies (−158,024, −121,516, and −121,426 kcal/mol) against *L*Tr, *La*A, and *Li*At active sites, respectively, while derivative 64 showed the second-best interaction energy (−155,221, −116,708, and −119,504 kcal/mol) in all three docking studies. Only in the case of the docking evaluation against the *La*A site (Figure 8b) did derivative 17, belonging to the NQ–Chlorine family, and derivatives 32, 33, 40, 41, and 43 of the NQ–amino family, show a better affinity interaction than member 63 of the NQ–triazole family.

These results conform with some studies of drugs used to combat parasitic diseases based on triazoles or azoles. These compounds lead to alterations in the mitochondria and accumulation of lipid bodies, thus interfering with the biosynthesis of the cell membrane [89], and leading to cell death of the parasite [90,91]. Thus, triazole-substituted naphthoquinone derivatives have potential antiparasitic activity against *Leishmania*, since they have also been shown to be a type of compound tolerable by patients [89].

**Figure 9:**
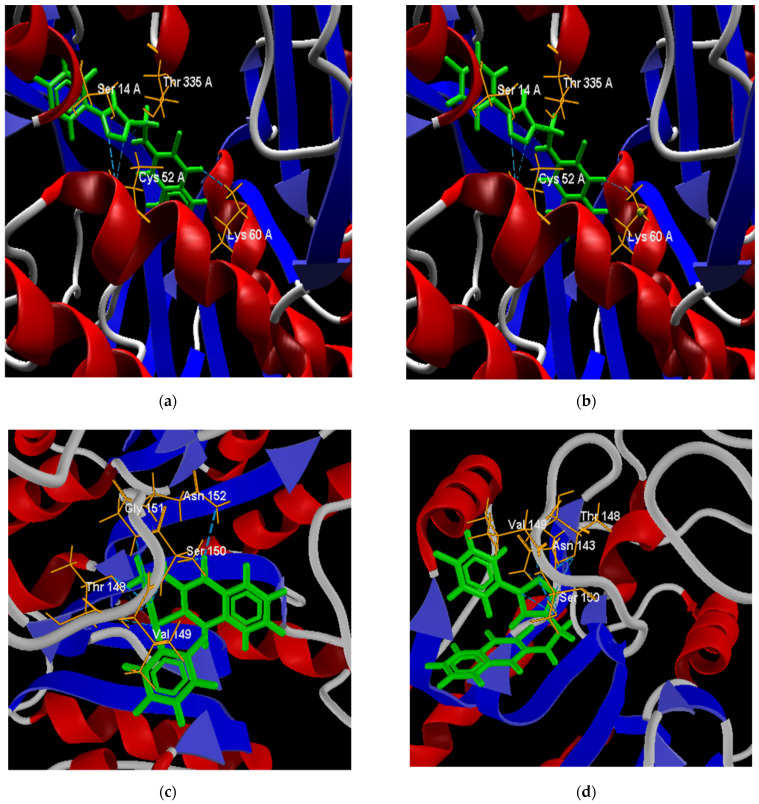

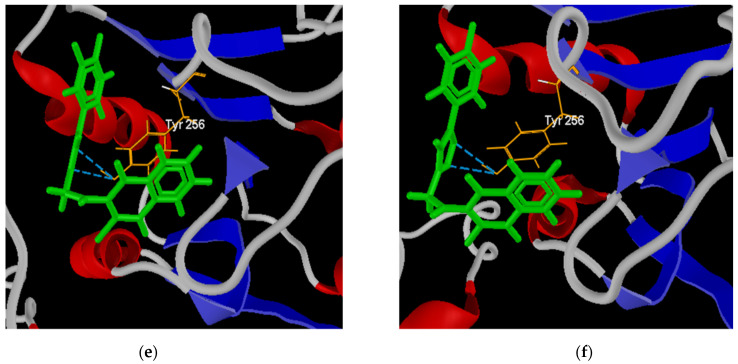
Best poses of derivatives: 64 (**a**) and 65 (**b**) in the active site of the *L*Tr protein; 64 (**c**) and 66 (**d**) in the active site of the *La*A protein; and 64 (**e**) and 67 (**f**) in the active site of the *Li*At protein.

The two best poses adopted within the active site for each docking evaluation are presented in Figure 9. In the case of the poses of derivatives 64 and 65 against the *L*Tr protein (Figure 9a,b), both present interactions by hydrogen bonds with the amino acid residues Ser 14, Thr 335, Cys 52, and Lys 60 of *L*Tr. The two cysteine residues (Cys52 and Cys57) present in the active site form the disulfide bond in the oxidized form of the protein [92], which is a bond critical in the parasite’s defense mechanism, while the interactions with the residues Thr 335 and Lys 60 are destined exclusively to the binding domain of FAD [93]. In this way, the binding of the ligands to these last residues prevents the binding of FAD and its orientation toward the active site during the reduction, which inhibits the *L*Tr enzyme [93].

The best positions of structures 64 and 66 in the active site of the *La*A protein (Figure 9c,d) presented both interactions by hydrogen bonds with residues Ser 150, Thr 148, and Val 149. Structure 64 presented hydrogen bonds with residues Gly151 and Asn 152, while structure 66 formed an interaction with residue Asn 143. In similar studies [94,95], some flavonoid ligands showed interactions with amino acid residues Ser150, Asn 152, and Asn153 within the active site, exhibited high in vitro activity against L. *amazonensis* cultures, and low toxicity in mammalian cells. Residues Ser150 and Asn143 are involved in the Mn(II) metal bridge in coordination with the active site of arginase, which is required to conduct its catalytic activity [95,96]. According to this, molecules 64 and 66 inhibit the coupling of the metallic bridge, and would be good inhibitors of the arginase protein.

The poses of structures 64 and 67 facing the *Li*At receptor are presented in Figure 9e,f. Both structures present interaction by hydrogen bonds with the residue Tyr 256. It has been shown that this residue prevents the rotation of the pyridine ring of the pyridoxal phosphate cofactor until the entrance of the amino acid, avoiding its stability [97].

### 2.3. ADME Analysis

Theoretical early estimation of ADME properties for the series of 68 1,4-naphthoquinone derivatives was performed in the free SwissADME web tool [98]. The data of lipophilicity, solubility, pharmacokinetic properties, and skin permeability parameters are collected in Table S21.

**Figure 10:**
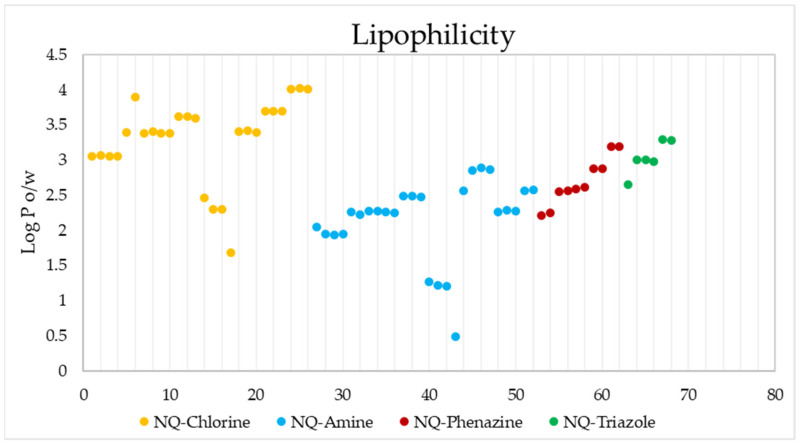
Lipophilicity values of the 68 naphthoquinone derivatives.

Figure 10 shows the estimated lipophilicity values of the 68 ligands, expressed as the logarithm of the octanol–water partition coefficient (Log P_o/w_). This parameter makes it possible to determine hydrophobicity, which is a parameter considered in the initial phases of drug development, and which allows inferring how the compound will behave in biological fluids and its possible diffusion through biological membranes [77,99]. The lipophilic results show that the 68 derivatives have a Log *p* < 5 [100], so according to the Lipinski Rules, they can be administered orally [101]. NQ–Amine derivative 43 presented the lowest value (log P_o/w_ = 0.49), that is, it is more hydrophilic than the rest.

**Figure 11:**
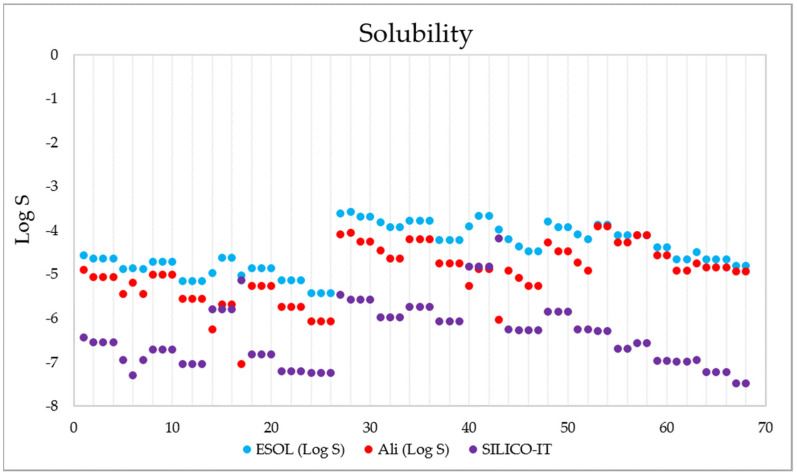
Solubility data (in Log S) calculated for the 68 1,4-naphthoquinone derivatives using the ESOL (blue), Ali-adapted (orange), and SILICO-IT (magenta) methods.

Figure 11 shows the SWISSADME solubility values (expressed as log S) of the 68 derivatives according to the ESOL (Estimated SOLubility), solubility adapted by Ali et.al., and SILICO-IT [102] methods. The ESOL method estimates the solubility in water directly from the molecular structure, followed by its weight [103]; the Ali method incorporates the effect of the topological polar surface area [104]; and the SILICO-IT method is calculated using a fragmented procedure [102]. The solubility (Log S) scales used are insoluble < −10, poor < −6, moderate < −4, soluble < −2, and high < 0. The calculated solubility data presented ESOL values of −5.42 < Log S < −3.57, Ali values of −7.04 < Log S < −3.9 and SILICO-IT values of −8.33 < Log S < −4.28, indicating that all 68 compounds have moderate to poor water solubility.

**Figure 12:**
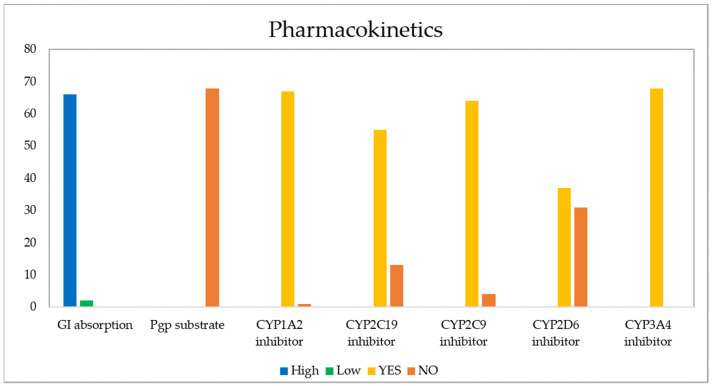
Pharmacokinetic properties calculated for the 68 naphthoquinone derivatives.

The evaluation of pharmacokinetic parameters of gastrointestinal (GI) absorption, P-gp substrate, and cytochrome P450 (CYP) inhibition or interaction for all 68 derivatives is presented in Figure 12.

**Figure 13:**
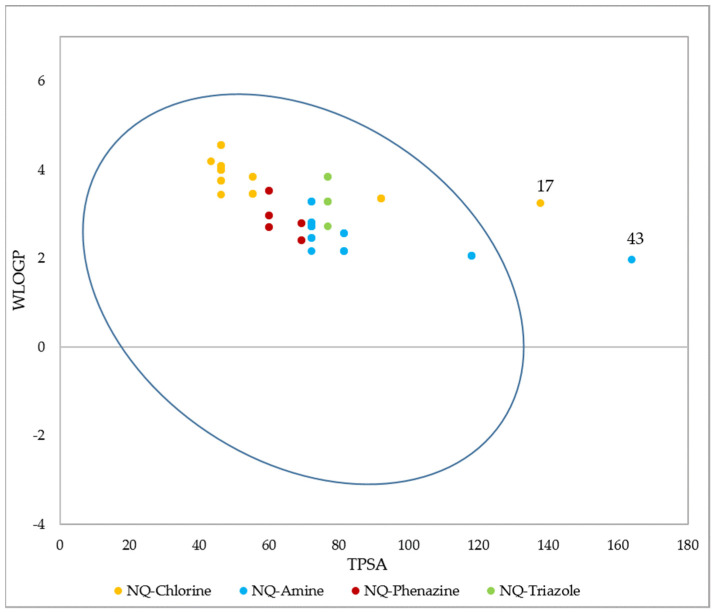
BOILED-Egg (adapted with permission from Ref. [108]) evaluation of gastrointestinal absorption for the 68 1,4-naphthoquinone derivatives. Molecules inside the oval indicate that they have GI permeability.

The GI barrier has a complex structure given the characteristics of a semi-permeable membrane, which allows fat-soluble molecules to penetrate it through a diffusion process, as is the case with most drugs [105]. As Figure 13 shows, passive human gastrointestinal (GI) absorption data estimate that 97% of the 68 compounds present high absorption in the digestive tract, while the remaining 3%, corresponding to structures 17 and 43, would present low intestinal absorption. For its part, P-glycoprotein (PGP) is a permeability protein that pumps substances out of the body and prevents their absorption, which causes drug resistance to form [105,106]. It was found that none of the compounds behaved as a substrate for P-gp; therefore, they could conduct the function without any resistance [107].

**Figure 14:**
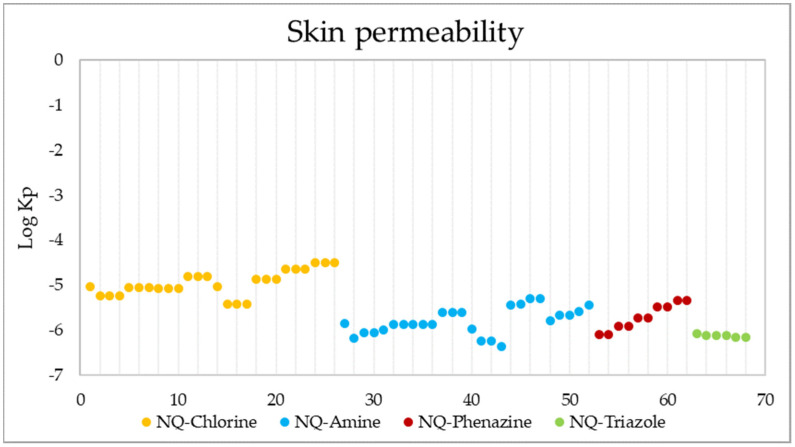
Skin permeability values in Log Kp (Kp in cm/s) of the 68 derivatives.

Another form of drug administration is through the skin (transdermal distribution), which allows the transport of substances through the epidermis. This parameter is related to the skin permeability coefficient (Kp), whose values indicate that the more negative the log Kp (with Kp in cm/s), the less permeant the molecule is [109,110]. Figure 14 shows the skin permeability of the 68 evaluated compounds, where it is evident that the best candidates to be administered transdermally are NQ–Chloro derivatives; among them, the best are 25, 26, and 27, while the ligand with the lowest permeability is 43.

The main enzyme isoforms CYP1A2, CYP2C19, CYP2C9, CYP2D6, and CYP3A4 [98] are involved in drug metabolism, and inhibitors block their metabolic activity in a dose-dependent manner. Structure 43 was found to be the only derivative unable to inhibit CYP1A2, while 13 derivatives (36–38, 40–43, and 54–58), 4 derivatives (35–37, and 57), and 31 derivatives (11–16, 24–30, 40–43, 55–68) projected a negative inhibitory response of CYP2C19, CYP2C9, and CYP2D6, respectively. In each of these cases, the activity of the remaining molecules would be affected by their metabolism, due to their ability to bind to these enzymes, which may generate adverse effects such as high toxicity [111,112,113]. None of the 68 molecules reported the inhibition of CYP3A4.

Based on a similarity to drugs, filters based on Lipinski (Pfizer) [101], Ghose (Amgen) [114], Veber (GSK) [115], Egan (Pharmacia) [116], and Muegge (Bayer) [117] rules were also evaluated, as well as the bioavailability score. These filters qualitatively define the feasibility of a compound to become an oral drug candidate. The results show that 100% of the 1,4-naphthoquinone derivatives comply with the Lipinski and Ghose rules, and 98.5% comply with the Veber and Egan rules. For these last two cases, molecule 43 presented a violation, caused by a value of TPSA = 163.83 Å^2^ that is above the established ranges (TPSA ≤ 140 and TPSA ≤ 131.6, respectively). Finally, the Muegge filter indicated that 90% of the derivatives present valid conditions to become oral drugs. The remaining 10% presented a violation since their XLOGP value is outside of the allowed range (−2 ≤ XLOGP ≤ 5) (structures 21–26), while the TPSA value of structure 43 is again above the range settled down. All compounds had a bioavailability value of 0.55, indicating oral bioavailability based on Lipinski’s rules. Additionally, it was established that an AB score of 0.55 is required to be considered a sufficiently absorbable molecule orally [118].

## 3. Materials and Methods

### 3.1. QSAR Modelling

#### 3.1.1. In Vitro Anti-Chagas and Anti-Leishmaniasis Data

All of the in vitro data used for constructing of the antiparasitic and toxicity QSAR models were taken from the literature. For constructing the anti-chagasic activity model, 153 structures derived from naphthoquinone were used, some of which were fused with triazoles and oxanes, among other heterocycles (Figure A1). All of these derivatives showed anti-*T. cruzi* activity, evaluated in vitro under the following experimental conditions. The stock solutions of the compounds were prepared in DMSO. Strain Y trypomastigotes were obtained at the peak of parasitemia from albino mice, then isolated through differential centrifugation, and resuspended in Dulbecco’s Modified Eagle Medium (DME), with a concentration of 107 parasite cells/mL in the presence of 10% mouse blood. A total of 100 µL of this solution was added to 100 µL of the compound solutions. The cell count was determined in a Neubauer chamber, and the trypanocidal activity was expressed as IC_50_, which corresponds to a concentration that allows the lysis of 50% of the parasites [23,24,25,26,27,28,29,30,31].

For the QSAR modeling of cutaneous anti-leishmaniasis (*L. amazonensis*) activity, 60 molecules (Figure A2) containing functional groups such as quinone, triazole, indole, and amine with high trypanocidal activity reported as IC_50_ were used. The anti-leishmanial activity of these structures was evaluated by tests with 3-(4,5-dimethylthiazol-2-yl)-2,5-diphenyltetrazolium (MTT) bromide. In these bioassays, promastigotes were seeded in RPMI medium supplemented with 10% FBS, and cultured in a 96-well plate with a concentration of 10 5 cells/plate at 37 °C. After the seeding period, the MTT solution was added, the formed complex was dissolved in DMSO, the supernatant was removed, and the cells were incubated under the same seeding conditions in the presence of various concentrations of the compounds. The concentration that inhibited 50% of parasite growth was determined as IC_50_ by linear regression [18,32,33,34,35,36,37,38,39].

For constructing the QSAR model of anti-visceral leishmaniasis (*L. infantum*) activity, 90 molecules (Figure A3) derived from quinone, triazole, and indole with activity reported as IC_50_ were used. The anti-leishmanial activity evaluation for these molecules was conducted using incubation in RPMI medium with 12% fetal calf serum (FBS), at a concentration of 105 cells/mL, for 48 h at 25 °C. After the incubation process, promastigote growth was estimated by counting the parasites with a Neubauer hemocytometer. The 50% inhibitory concentration (IC_50_) was defined as the drug concentration required to inhibit 50% of the parasite growth [40,41,42,43,44,45].

The QSAR model of toxicity was built from 76 structures (Figure A4) derived from naphthoquinone, some fused with triazoles, pyrazoles, imidazoles, and aromatic rings, with experimental data measured according to the following parameters. Mouse fibroblasts (L929) were used and determined by the reduction of 3-(4,5-dimethyl-2-thiazol)-2,5-diphenyl-2H-tetrazolium bromide (MTT), expressed as IC_50_ [30,46,47,48,49,50,51].

#### 3.1.2. Development of Antiprotozoal QSAR Models

All molecules were processed in MDL mol (V2000) format using the ACD/I-Lab software version 11.0 13 [119]. The calculation of 83,180 fingerprint-type, one-dimensional, and two-dimensional molecular descriptors was performed in the programs PaDEL-Descriptor [54], Mold2 [120], QuBiLS-MAS [56], and Fragmentor [121] (all free access). By using the balanced subsets method, the data sets were divided into calibration, validation, and test groups, using 70, 15, and 15%, for the anti-chagas activity model, 80%, 10%, and 10% for the anti-leishmanial activity models, and 70, 15, and 15% for toxicity, respectively. Subsequently, the Replacement Method (RM) [122], which is available in the MatLab programming language, was applied to explore the set of descriptors. RM is an unequivocal algorithm based on multivariable linear regression (MLR), which searches for the best subsets of descriptors in large databases. By using the RM algorithm, multivariable regression models of up to seven descriptors were generated for each model, which was carried out in the MATLAB software version 7.12.0 [123].

#### 3.1.3. Validation of the QSAR Models

Validation of all QSAR models was performed through both internal and external validation processes. Internal validation was performed using the LOO (Leave-One-Out) validation method. For the internal validation of each QSAR model, the statistical parameters R2 LOO (square correlation coefficient of LOO) and SLOO (standard deviation of LOO) were determined. Additionally, the statistical robustness of the models was determined using randomization Y [124,125]. For its part, the external validation verified the predictive capacity, applying the methodology proposed by Golbraikh and Tropsha [78], where the correlation coefficients between the predicted and experimental properties of the compounds in the test set are estimated.

### 3.2. Molecular Docking

The structures of the macromolecular targets used for molecular docking were extracted from the Protein Data Bank (PDB). In the case of *T. cruzi*, trypanothione reductase (*Tc*TR) [86] and lanosterol α-demethylase (*Tc*LαD) [87] proteins were selected, with 1BZL and 2WUZ coding and a resolution of 2.40 Å and 2.45 Å, respectively. For the *Leishmania* genus in general, the protein trypanothione reductase (*L*TR) [126,127] with code 2JK6 and a resolution of 2.95 Å was selected, while for the strains *L. amazonensis* and *L. infantum* arginase (*La*A) [128] and tyrosine aminotransferase (*Li*TA) [129] were selected, with codes 4IU0 and 4IX8 and resolutions of 1.77 Å and 2.35 Å, respectively.

**Figure 15:**
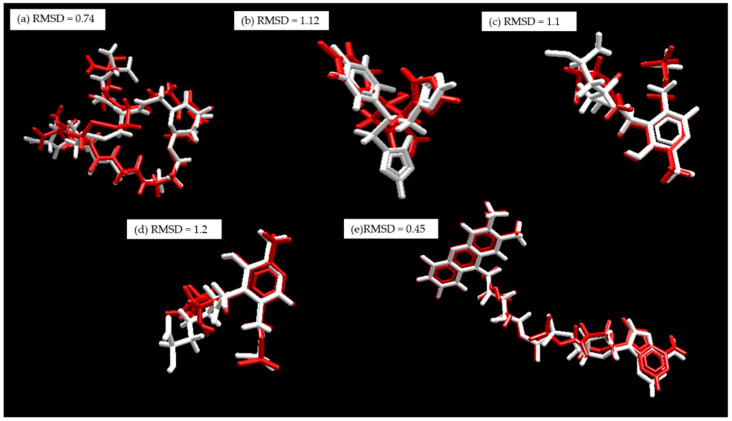
Re-coupling, white: co-crystallized substrate. Red: Substrate coupled using MVD. (**a**) *Tc*TR; (**b**) *Tc*LαD, (**c**) *Li*TA, (**d**) *La*A, and (**e**) *L*TR.

Before the molecular docking simulations, the proteins were prepared in the Molegro Virtual Docker (MVD) program [130], where bond assignment, bond order, hybridization, missing hydrogens, charge assignment, and atom tripos were performed. To validate the software and optimize the molecular docking parameters, a re-docking procedure of the co-crystallized ligands was carried out. For each protein, a cavity was created by delimiting the coupling space of the co-crystallized ligand, and the best pose (Figure 15) was selected based on the lowest RMSD value (<2) [131].

For the preparation of the ligands, the structures of the naphthoquinone derivatives (68) were built in the ACD-Labs program, and saved as .mol files. These structures were then optimized in the Gaussian 09W program [132] by simultaneously relaxing all geometric parameters to the theoretical level B3LYP/6–31 + G(d).

For the molecular docking simulations, 5 poses (conformation and orientation) were generated for each of the 68 ligands evaluated, using the search algorithm MolDock Optimizer, which was executed with 10 repetitions. The data analysis was performed in the Molegro Virtual Modeller (MVM) program, where the best pose in each case was selected, and the data were plotted based on the energy calculated using the scoring function (Equation (5)).

(5) $$E_{score}=E_{inter}+E_{intra}$$

where $E_{inter}$ is the intermolecular interaction energy of the ligand–protein, and the $E_{intra}$ is the internal energy of the ligand. The $E_{inter}$ is shown in Equation (6):

(6) $$E_{inter}=\sum_{i\in ligand}\sum_{i\in ligand}\left[E_{PLP}\left(r_{ij}\right)\right]+332.0\frac{q_{i}q_{j}}{4r_{ij}^{2}}$$

where the EPLP term represents the PLP (piecewise linear potential) energy, which consists of the use of two different parameter sets, described as follows: one for approximation of the steric term (van der Waals) among atoms, and the other potential for the hydrogen binding. The second term ($332.0\;\frac{q_{i}q_{j}}{4r_{ij}^{2}}$) is related to the electrostatic interactions among overloaded atoms. It is a Coulomb potential with a dielectric constant dependent on the distance (D(r) = 4r). The numerical value of 332.0 is responsible for the electrostatic energy unit to be given in kilocalories per mol. The *q_i_* and *q_j_* terms represent the charges of the atoms *i* and *j*, respectively. The *r_ij_* term indicates the interatomic distance between the atoms *i* and *j* [133].

The $E_{intra}$ is shown in Equation (7).

(7) $$E_{inter}=\sum_{i\in ligand}\sum_{i\in ligand}\left[E_{PLP}\left(r_{ij}\right)\right]+\sum_{flexible\;bonds}A\left[1-cos\left(m.\theta -\theta_{0}\right)\right]+E_{Clash}$$

The first part of the equation (double summation) is among all pairs of atoms in the ligand, taking off those which are connected by two bonds. The second term characterizes the torsional energy, where *θ* is the torsional angle of the bond and *θ*_0_ is its corresponding value in the equilibrium. The average of the torsional energy bond contribution is used if several torsions could be determined. The last term, *E_clash_*, assigns a penalty of 1.000 if the distance between two heavy atoms (more than two bonds apart) is shorter than 2.0 Å, not considering infeasible ligand conformations [134]. The docking search algorithm that is applied in the MVD program considers an evolutionary algorithm, the interactive optimization techniques which are inspired by Darwinian evolution theory, and a new hybrid search algorithm called guided differential evolution. This hybrid combines the differential evolution optimization technique with a cavity prediction algorithm during the search process, thereby allowing for fast and accurate identification of potential binding modes (poses) [133,134,135].

### 3.3. ADME Analysis

The ADME study was conducted using the SWISSADME web tool of the Swiss Institute of Bioinformatics [98]. This free web tool allows you to calculate properties, such as lipophilicity, water solubility, pharmacokinetics, and drug similarity, based on the structure or SMILE of the molecule.

## 4. Conclusions

A total of four QSAR models based on naphthoquinone derivative structures were developed and validated, three of them for antiprotozoal activity against *T. cruzi, L. amazonensis*, and *L. infantum*, and one for toxicity prediction. All QSAR models were built using in vitro inhibitory concentrations (IC_50_) which were previously reported. The anti-*T. cruzi* model was developed based on 153 molecules, the anti-*L. amazonensis* used 60 molecules, the anti-*L. infantum* model used 90 structures, and the toxicity model was built with 76. According to the anti-*T. cruzi* QSAR model, the anti-chagasic activity is favored by the refractivity, electronegativity, and polarizability of the molecules. These characteristics possibly give them a better binding capacity and inhibition of essential macromolecules in the metabolism of the parasite. Additionally, it was found that the presence of aromatic fragments in these structures increases their antiparasitic activity. Of the proposed molecules, structures 61 and 62, belonging to the derivatives fused with phenazine, and 24, 25, and 54, which are phenylamino derivatives, presented a better activity against *T. cruzi* predicted by the QSAR model compared to that of the reference drug. The QSAR anti-*L. amazonensis* predicts that molecules with less symmetry and with the presence of secondary and tertiary carbons will be more active. Thus, phenazines 62, 60, 59, 61, 58, and 57 showed a predicted potential activity against *L. amazonensis*, as well as derivatives 56, 27, and 26 of the phenylamine family. A total of 22 structures showed better predicted activity than Miltefosine, and 37 demonstrated better results than Glucantime. According to the anti-*L. infantum* QSAR model, the activity is a function of LogP, which is an essential characteristic for ligands to be considered drugs, since it gives them the ability to permeate biological membranes. Similarly, the activity increases with PSA, which is related to the penetration of the blood–brain barrier and its intestinal absorption. The derivatives with the best predicted anti-*L. infantum* activity were 6, 15, 7, 14, and 16, which belong to the phenylamino family. Additionally, it is highlighted that 23 molecules were more active than the reference drugs (Miltefosine and Glucantime).

According to the results of molecular docking, all the evaluated compounds present very favorable interaction energies with the selected receptors, which are essential in the metabolism of both *T. cruzi* and *Leishmania*. In the *Tc*TR protein, the derivatives fused with triazole stand out. The best of these was structure 64, which, unlike its analogs, features two hydrogen bonds between the triazole ring and the Gly 459 residue. Similarly, the triazole derivatives presented the best interaction energies for the *Tc*LαD protein. The best of them was structure 66. This derivative presents three interactions of hydrogen bonds between the triazole ring and the Tyr 103 residue, which is a fundamental residue in the active site of said receptor. The triazole derivatives 65, 66, and 67 presented the best interaction affinities against the catalytic sites of all leishmania proteins evaluated (Trypanothione reductase, Arginase, and Tyrosine Aminotransferase). These types of derivatives have a wide variety of biological activities, and according to our results, they are considered potent inhibitors of macromolecular targets. Ligands belonging to this type of molecules are currently used as active principles of antiparasitic drugs because they generate cell deformation and alterations in the mitochondria, thus interfering with the biosynthesis of the parasite’s cell membrane, causing cell death.

The results of ADME showed that the 68 evaluated compounds met the requirements to be administered orally. ADME calculations included lipophilicity, solubility, pharmacokinetics, and drug-likeness.

## Figures and Tables

**Table 1:** Molecular descriptors of the best anti-chagas QSAR model as defined by Equation (1).

Descriptor	Name	Short Description		Type
*a*_1_	frag16	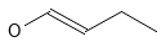		Fingerprint
*a*_2_	MACCSFP72	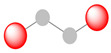	(Any bond; Red: Oxygen; Gray:Any atom)	Fingerprint
*a*_3_	K_Q_AB_nCi_2_SS10_T_KA_r_MAS	Refractivity		2D
*a*_4_	K_B_AB_nCi_2_DS7_C_KA_e-p_MAS	Electronegativity/Polarizability		2D
*a*_5_	K_B_AB_nCi_2_DS2_X_KA_r-c_MAS	Refractivity/Charge		2D
*a*_6_	N2_F_AB_nCi_2_NS7_P_KA_m_MAS	Mass		2D

**Table 2:** Molecular descriptors in the anti-*L. amazonensis* QSAR model, as described by Equation (2).

Descriptor	Name	Short Description		Type
*b*_1_	KRFP2	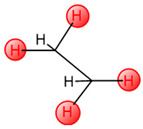	(Klekota–Roth fingerprint, presence of chemical substructures)	Fingerprint
*b*_2_	minHBint9	Electro-topological state atom type descriptor (minimal strength E-state type descriptor for potential hydrogen bonds of path length 9)		2D
*b*_3_	IC3	Information content index (3-order neighborhood symmetry)		2D
*b*_4_	MDEC-23	Molecular edge distance		2D

**Table 3:** Molecular descriptors in the anti-*L. infantum* QSAR model, as described by Equation (3).

Descriptor	Name	Short Description	Type
*c*_1_	MATS3c	Moran Correlation—lag 3/load-weighted	2D
*c*_2_	nHBint7	Atom-like electro-topological state	2D
*c*_3_	AM_F_AB_nCi_2_NS12_T_KA_a_MAS	Alog P (partition)	2D
*c*_4_	AM_B_AB_nCi_2_NS2_C_KA_psa-v_MAS	C atoms in aliphatic chain Topological area of the polar surface/Vdw volume	2D
*c*_5_	AM_B_AB_nCi_2_SS1_C_KA_v-c_MAS	Volume of Vdw/Charge	2D
*c*_6_	AM_Q_AB_nCi_2_NS15_X_KA_a_MAS	Heteroatom–Partitioning Algorithm (Alog P)	2D

**Table 4:** Molecular descriptors in the QSAR model of toxicity as described by Equation (4).

Descriptor	Name	Short Description	Type
*d*_1_	K_B_AB_nCi_2_NS3_T_KA_psa-r_MAS	Topological polar surface area; refractivity	2D
*d*_2_	K_B_AB_nCi_2_DS7_P_KA_c-p_MAS	Aromatic C atoms. Charge; polarization	2D
*d*_3_	N2_B_AB_nCi_2_MP4_P_KA_psa-p_MAS	Aromatic C atoms. Topological polar surface area; polarization	2D
*d*_4_	K_B_AB_nCi_2_DS3_X_KA_a-e_MAS	Heteroatoms. Partition algorithm (Log P); electronegativity	2D
*d*_5_	K_Q_AB_nCi_2_SS14_C_KA_c_MAS	C atoms in aliphatic chain. Charge.	2D

**Table 5:** Internal and external validation parameters for each best QSAR model.

Parameter	QSAR Models				
	Anti-Chagas (Ec. 1)	anti-*L. amazonensis* (Ec. 2)	Anti-*L. infantum* (Ec. 3)	Toxicity (Ec. 4)	
				98%	100%
$N_{train}$	107	48	72	60	60
$N_{val}$	23	6	9	8	8
$N_{test}$	23	6	9	7	8
$R_{test}^{2}$	0.83	0.78	0.66	0.91	0.70
$RMS_{test}$	0.48	0.38	0.35	0.22	0.32
$R_{ijmax}^{2}$	0.40	0.19	0.79	0.44	0.44
$R_{loo}^{2}$	0.71	0.70	0.66	0.92	0.92
$RMS_{loo}$	0.54	0.35	0.46	0.18	0.18
$R_{Rand}^{2}$	0.06	0.09	0.08	0.08	0.09
$RMS_{Rand}$	0.10	0.59	0.74	0.74	0.95
*k*	1.12	1.03	1.03	0.96	0,97
*k*’	0.86	0.88	0.92	1.03	1.00
$Q_{F1}^{2}$	0.80	0.80	0.71	0.89	0.75
$Q_{F2}^{2}$	0.76	0.77	0.65	0.82	0.63
$Q_{F3}^{2}$	0.77	0.65	0.80	0.88	0.75
*CCC*	0.86	0.86	0.79	0.89	0.83
$O_{3}$	0	0	0	0	1

## Data Availability

Data is contained within the article and Supplementary Material.

## Supplementary Materials

The following supporting information can be downloaded at: https://www.mdpi.com/article/10.3390/ph15060687/s1, Figure S1: Cavities in the active site of TcTR (a) and TcLαD (b) proteins; Figure S2: Cavities in the active site of LTR (a). LaA (b). and LiTA (c) proteins; Table S1: Antiparasitic QSAR models for chagas and leishmaniasis protozoa based on naphthoquinone derivatives obtained by the replacement method; Table S2: Toxicity QSAR models based on naphthoquinone derivatives obtained by the replacement method; Table S3: Intercorrelation matrix of the QSAR model descriptors for anti-chagas activity; Table S4: Numerical values of the QSAR anti-chagas model descriptors; Table S5: Experimental and predicted values of anti-chagas activity. Residuals and influence values h (h^lim = 0.196) are reported. ^ validation group and * test group; Table S6: Values predicted by the QSAR model of anti-chagas activity of the 68 naphthoquinone derivatives (prediction group); Table S7: Intercorrelation matrix of the QSAR model descriptors for anti-leishmanial activity; Table S8: Numerical values of the QSAR anti-L. amazonensis model descriptors; Table S9: Numerical values of the QSAR anti-L. infantum model descriptors; Table S10: Experimental and predicted values of anti-L. amazonensis activity. Residuals and influence values h (h^lim = 0.3125) are reported. ^ validation group and * test group; Table S11: Experimental and predicted values of anti-L. infantum activity. Residuals and influence values h (h^lim = 0.24) are reported. ^ validation group and * test group; Table S12: Values predicted by the QSAR models of anti-leishmanial activity of the 68 naphthoquinone derivatives (prediction group); Table S13: Intercorrelation matrix of the QSAR model descriptors for toxicity; Table S12: Numerical values of the QSAR toxicity model descriptors Table S14. Numerical values of the QSAR toxicity model descriptors; Table S15: Experimental and predicted values of toxicity. Residuals and influence values h (h^lim = 0.3125) are reported. ^ validation group and * test group; Table S16: Values predicted by the QSAR model of toxicity of the 68 naphthoquinone derivatives (prediction group); Table S17: Results of the coupling of naphthoquinone derivatives in the active site of the TcTR and TcLαD proteins. Table S18: Results of the docking of naphthoquinone derivatives in the active site of the LTR protein; Table S19: Results of the docking of naphthoquinone derivatives in the active site of the LaA protein; Table S20: Results of the docking of naphthoquinone derivatives in the active site of the LiTA protein; Table S21: ADME parameters.
