# Supplementary material for: In Silico Antiprotozoal Evaluation of 1,4-Naphthoquinone Derivatives against Chagas and Leishmaniasis Diseases Using QSAR, Molecular Docking, and ADME Approaches
Source: Pharmaceuticals (Basel). 2022 May 31;15(6):687. doi: 10.3390/ph15060687 (PMC9228275; doi:10.3390/ph15060687)
Supplement: Supplementary file 1 [file pharmaceuticals-15-00687-s001.zip › pharmaceuticals-1729941-supplementary.pdf]

Supplementary Materials

In Silico Antiprotozoal Evaluation of 1.4-Naphthoquinone Derivatives against Chagas and Leishmaniasis Diseases Using QSAR, Molecular Docking, and ADME Approaches

Lina S. Prieto Cárdenas [1], Karen A. Arias Soler [1], Diana L. Nossa González [1], Wilson E. Rozo Núñez [1], Agobardo Cárdenas-Chaparro [1], Pablo R. Duchowicz [2], and Jovanny A. Gómez Castaño [1,*]

[1] Grupo Química-Física Molecular y Modelamiento Computacional (QUIMOL), Facultad de Ciencias, Universidad Pedagógica y Tecnológica de Colombia (UPTC), Avenida Central del Norte, Tunja, Boyacá 050030, Colombia; grupo.quimol@uptc.edu.co

[2] Instituto de Investigaciones Fisicoquímicas Teóricas y Aplicadas (CONICET – Universidad Nacional de La Plata). Diagonal 113 y calle 64. C.C. 16. Sucursal 4. 1900. La Plata, Provincia de Buenos Aires, Argentina

* Correspondence: jovanny.gomez@uptc.edu.co

Table S1. Antiparasitic QSAR models for chagas and leishmaniasis protozoa based on naphthoquinone derivatives obtained by the replacement method. Best models in bold.

Model	N°	Descriptors	R^2_{train}	$RMSE_{train}$	R^2_{val}	$RMSE_{val}$
**Anti-*T. cruzi***	1	JGI8	0.41	0.78	0.53	0.78
	2	MACCSFP72 • AM_Q_AB_nCi_2_MP8_P_KA_p_MAS	0.53	0.70	0.62	0.70
	3	frag75 • MACCSFP72 • N2_B_AB_nCi_2_MP0_D_KA_e-p_MAS	0.64	0.61	0.71	0.61
	4	PubchemFP605 • K_B_AB_nCi_2_SS2_C_KA_psa-r_MAS • K_B_AB_nCi_2_SSS2_P_KA_psa-c_MAS • N2_F_AB_nCi_2_MP6_C_KA_psa_MAS	0.69	0.56	0.73	0.58
	5	D288 • MACCSFP72 • MATS5v • K_B_AB_nCi_2_DS8_T_KA_e-c_MAS • K_Q_AB_nCi_2_DS3_C_KA_e_MAS	0.72	0.53	0.71	0.58
	**6**	**frag16 • MACCSFP72 • K_Q_AB_nCi_2_SS10_T_KA_r_MAS • K_B_AB_nCi_2_DS7_C_KA_e-p_MAS • K_B_AB_nCi_2_DS2_X_KA_r-c_MAS • N2_F_AB_nCi_2_NS7_P_KA_m_MAS**	**0.76**	**0.50**	**0.82**	**0.52**
	7	KRFP4820 • MATS5v • K_B_AB_nCi_2_DS2_T_KA_psa-p_MAS • K_F_AB_nCi_2_DS3_T_KA_p_MAS • K_B_AB_nCi_2_NS14_A_KA_psa-c_MAS • K_B_AB_nCi_2_NS3_P_KA_psa-r_MAS • K_F_AB_nCi_2_NS5_M_KA_a_MAS	0.80	0.45	0.76	0.53
**Anti-*L. amazonensis***	1	KRFPC3710	0.52	0.44	0.79	0.51
	2	KRFP2 • SdssC	0.62	0.39	0.75	0.40
	3	KRFP2 • VE3_Dt • minHBint9	0.72	0.34	0.87	0.33
	**4**	**KRFP2 • minHBint9 • IC3 • MDEC-23**	**0.77**	**0.30**	**0.84**	**0.31**
	5	KRFP2 • D172 • VC-5 • VR1_Dt • minHBint9	0.83	0.26	0.92	0.19
	6	KRFP2 • PubchemFP206 • KRFPC3719 • AATSC2v • E3_Dt • SHBint8	0.86	0.24	0.89	0.26
	7	KRFP2 • KRFP133 • KRFPC3440 • APC2D7_C_O • ATSC8v • minHBint9 • SD_B_AB_nCi_2_DS9_A_KA_psa-c_MAS	0.89	0.21	0.90	0.22
**Anti-*L. infatum***	1	AM_B_AB_nCi_2_NS0_X_KA_a-p_MAS	0.52	0.44	0.79	0.51
	2	AATSC3c • AM_B_AB_nCi_2_NS0_X_KA_a-p_MAS	0.62	0.39	0.75	0.40
	3	PubchemFP503 • MATS3c • AM_B_AB_nCi_2_NS0_X_KA_a-p_MAS	0.72	0.34	0.87	0.33
	4	D470 • MATS3c • AM_F_AB_nCi_2_SS1_C_KA_psa_MAS • AM_Q_AB_nCi_2_NS13_X_KA_a_MAS	0.77	0.30	0.84	0.31
	5	D470 • MACCSFP159 • MATS3c • AM_B_AB_nCi_2_DS1_C_KA_psa-v_MAS • AM_Q_AB_nCi_2_NS13_X_KA_a_MAS	0.83	0.26	0.92	0.19
	**6**	**MATS3c • nHBint7 • AM_F_AB_nCi_2_NS12_T_KA_a_MAS • AM_B_AB_nCi_2_NS2_C_KA_psa-v_MAS • AM_B_AB_nCi_2_SS1_C_KA_v-c_MAS • AM_Q_AB_nCi_2_NS15_X_KA_a_MAS**	**0.72**	**0.42**	**0.71**	**0.42**
	7	PubchemFP503 • APC2D10_C_Cl • AATSC3c • nHBint7 • AM_B_AB_nCi_2_SS4_C_KA_psa-v_MAS • AM_F_AB_nCi_2_SS1_C_KA_c_MAS • AM_Q_AB_nCi_2_NS13_X_KA_a_MAS	0.89	0.21	0.90	0.22

Table S2. Toxicity QSAR models based on naphthoquinone derivatives obtained by the replacement method. Best model in bold.

N°	Descriptors	R^2_{train}	$RMSE_{train}$	R^2_{val}	$RMSE_{val}$
1	N2_B_AB_nCi_2_MP7_P_KA_psa-p_MAS	0.79	0.29	0.75	0.35
2	KRFPC3455 • K_B_AB_nCi_2_DS3_X_KA_a-e_MAS	0.84	0.26	0.90	0.26
3	frag14 • K_B_AB_nCi_2_DS10_C_KA_psa-c_MAS • K_B_AB_nCi_2_DS3_X_KA_a-e_MAS	0.89	0.22	0.92	0.21
4	K_B_AB_nCi_2_DS1_X_KA_a-p_MAS • K_F_AB_nCi_2_SS2_P_KA_c_MAS • N2_F_AB_nCi_2_MP4_P_KA_psa_MAS • K_Q_AB_nCi_2_NS4_A_KA_v_MAS	0.93	0.17	0.83	0.27
**5**	**K_B_AB_nCi_2_NS3_T_KA_psa-r_MAS • K_B_AB_nCi_2_DS7_P_KA_c-p_MAS • N2_B_AB_nCi_2_MP4_P_KA_psa-p_MAS • K_B_AB_nCi_2_DS3_X_KA_a-e_MAS • K_Q_AB_nCi_2_SS14_C_KA_c_MAS**	**0.93**	**0.16**	**0.91**	**0.23**
6	frag74 • K_B_AB_nCi_2_NS6_C_KA_r-c_MAS • N2_B_AB_nCi_2_NS4_C_KA_psa-c_MAS • K_B_AB_nCi_2_SS3_X_KA_psa-v_MAS • K_B_AB_nCi_2_DS1_X_KA_a-v_MAS • K_Q_AB_nCi_2_SS5_P_KA_c_MAS	0.95	0.15	0.86	0.24
7	D470 • ASP-4 • K_B_AB_nCi_2_DS5_P_KA_c-p_MAS • K_B_AB_nCi_2_DS3_X_KA_a-e_MAS • K_F_AB_nCi_2_NS13_D_KA_c_MAS • K_F_AB_nCi_2_DS3_X_KA_a_MAS • K_Q_AB_nCi_2_NS0_A_KA_psa_MAS	0.96	0.13	0.83	0.27

Table S3. Intercorrelation matrix of the QSAR model descriptors for anti-Chagas activity.

Descriptors	a_1	a_2	a_3	a_4	a_5	a_6	'VIF'
a_1	1.000	0.256	0.080	0.077	0.024	0.007	1.096
a_2		1.000	0.400	0.180	0.090	0.009	1.517
a_3			1.000	0.245	0.000	0.127	1.353
a_4				1.000	0.004	0.199	1.177
a_5					1.000	0.092	1.049
a_6						1.000	1.137

Table S4. Numerical values of the QSAR antichagas model descriptors.

ID	a_1	a_2	a_3	a_4	a_5	a_6
**1**	0	0	1.496	-0.007	-0.676	13111.508
**2**	0	0	-0.311	-0.111	-1.751	13835.308
**3**	0	0	-0.277	-0.001	-1.489	13729.559
**4**	0	0	-0.257	0.039	-1.063	13700.292
**5**	0	0	-0.761	-0.438	-2.381	13861.989
**6**	0	0	-0.796	-0.290	-1.908	13753.676
**7**	0	0	-0.800	-0.229	-1.310	13723.545
**8**	0	0	0.756	0.172	-1.207	13759.096
**9**	0	0	0.867	0.161	-0.708	13662.295
**10**	0	0	0.910	0.158	-0.194	13635.485
**11**	0	0	1.406	0.187	-1.871	13936.721
**12**	0	0	1.522	0.173	-1.494	13820.680
**13**	0	0	1.577	0.169	-1.185	13788.173
**14**	0	0	-0.453	0.508	-2.157	14446.252
**15**	0	0	-0.375	0.502	-1.847	14275.599
**16**	0	0	-0.328	0.503	-1.655	14228.385

**17**	0	0	-2.170	1.029	-2.406	15688.225
**18**	0	0	0.537	-0.362	-0.414	13684.902
**19**	0	0	0.554	-0.268	-0.388	13596.148
**20**	0	0	0.573	-0.228	-0.380	13571.738
**21**	0	0	-0.160	-0.750	-0.124	13786.813
**22**	0	0	-0.177	-0.616	-0.083	13686.212
**23**	0	0	-0.171	-0.559	-0.078	13658.503
**24**	0	0	-0.617	-1.153	0.173	13911.535
**25**	0	0	-0.675	-1.042	0.222	13796.826
**26**	0	0	-0.684	-0.992	0.226	13765.212
**27**	0	0	0.386	0.027	0.262	12938.598
**28**	0	0	-0.824	-0.134	-1.203	13666.712
**29**	0	0	-0.837	-0.029	-0.992	13561.939
**30**	0	0	-0.834	0.011	-0.615	13532.849
**31**	0	0	-1.119	-0.516	-1.748	13693.570
**32**	0	0	-1.190	-0.383	-1.348	13586.263
**33**	0	0	-1.206	-0.325	-0.809	13556.318
**34**	0	0	-0.051	0.210	-0.502	13589.988
**35**	0	0	0.006	0.198	-0.041	13494.075
**36**	0	0	0.031	0.195	0.447	13467.411
**37**	0	0	0.399	0.226	-1.225	13768.792
**38**	0	0	0.430	0.210	-0.912	13653.665
**39**	0	0	0.450	0.206	-0.652	13621.316
**40**	0	0	-1.106	0.552	-1.604	14281.758
**41**	0	0	-1.158	0.544	-0.490	14854.082
**42**	0	0	-1.122	0.546	-0.485	14788.235
**43**	0	0	-2.443	1.080	-2.104	15533.840
**44**	0	0	-0.754	-0.657	0.917	13490.723
**45**	0	0	-1.014	-1.228	1.149	13743.456
**46**	0	0	-1.095	-1.149	1.237	13629.827
**47**	0	0	-1.115	-1.112	1.240	13598.431
**48**	0	0	-0.227	-0.385	0.516	13515.299
**49**	0	0	-0.239	-0.301	0.581	13427.421
**50**	0	0	-0.234	-0.264	0.593	13403.152
**51**	0	0	-0.705	-0.816	0.831	13617.895
**52**	0	0	-0.748	-0.705	0.912	13518.258
**53**	0	0	-0.216	3.325	-2.585	20485.618
**54**	1	0	-0.134	3.533	-2.015	20267.303
**55**	0	0	-0.665	1.494	-2.437	20512.441
**56**	1	0	-0.648	1.627	-1.595	20288.806
**57**	0	0	0.457	2.850	-1.153	20319.540

**58**	0	0	0.465	2.938	-1.131	20139.368
**59**	0	0	-0.127	1.110	-0.972	20426.307
**60**	0	0	-0.131	1.144	-0.887	20221.951
**61**	0	0	-0.547	0.097	-0.744	20553.937
**62**	0	0	-0.579	0.087	-0.630	20321.568
**63**	0	0	1.230	0.132	-1.352	13901.892
**64**	0	0	0.031	0.277	-0.683	14422.467
**65**	0	0	0.005	0.264	-0.948	14457.586
**66**	0	0	-0.050	0.265	-1.198	14584.850
**67**	0	0	-0.688	0.416	-1.389	15094.040
**68**	0	0	-0.690	0.417	-0.926	15158.033
**69**	2	1	-1.337	-1.666	-0.286	10706.019
**70**	1	1	-2.210	-1.450	-1.319	10837.766
**71**	1	1	-2.210	-1.811	-0.557	9985.253
**72**	0	0	5.295	0.680	-0.040	15172.831
**73**	0	0	0.560	0.585	-0.525	15775.276
**74**	1	1	-2.123	-1.801	-2.615	11065.610
**75**	1	1	-1.482	-1.523	-2.558	15667.925
**76**	1	1	-1.418	-1.567	-2.209	16199.941
**77**	1	1	-1.467	-1.538	-2.508	15933.594
**78**	1	1	-1.085	-1.240	-1.257	15337.065
**79**	1	1	-1.338	-1.235	-0.796	15870.217
**80**	1	1	-1.628	-1.120	-0.232	15932.004
**81**	1	1	-1.854	-1.163	-1.607	17108.500
**82**	1	1	-1.285	-1.229	-1.083	15801.566
**83**	1	1	-1.251	-1.402	-0.200	15582.794
**84**	1	1	-1.216	-1.416	-1.295	14542.430
**85**	1	1	-1.928	-1.222	0.102	15774.963
**86**	1	1	-1.769	-1.373	-0.661	15476.202
**87**	1	1	-1.588	-1.499	-0.885	11034.161
**88**	1	1	-1.729	-1.340	-1.021	15081.080
**89**	1	1	-1.761	-1.364	-0.649	15460.264
**90**	1	1	-1.572	-1.611	-0.173	11035.114
**91**	1	1	-1.239	-1.539	-0.665	11033.694
**92**	1	1	-1.162	-1.363	0.135	11036.211
**93**	1	1	-2.009	-1.631	-0.134	11056.678
**94**	1	1	-1.828	-1.621	-0.826	11132.035
**95**	1	1	-1.174	-1.287	2.537	15406.068
**96**	1	1	-1.349	-1.300	1.437	16555.827
**97**	1	1	-1.328	-1.300	3.314	15937.317
**98**	1	1	-1.574	-1.159	1.385	15998.861

**99**	2	1	-1.328	-1.593	-1.832	15103.452
**100**	0	0	2.836	-1.435	-3.039	13343.651
**101**	0	0	0.673	-1.176	-2.095	16440.801
**102**	2	0	1.448	-1.213	-1.959	14200.882
**103**	2	0	0.196	-0.981	-1.417	17354.751
**104**	2	0	1.158	-1.676	-0.606	14363.506
**105**	2	0	1.126	-1.524	-2.038	14563.279
**106**	2	0	0.918	-1.783	-1.945	14734.934
**107**	2	0	1.061	-1.624	-1.454	14395.389
**108**	2	0	0.878	-1.862	-1.182	14557.470
**109**	0	0	1.442	-1.550	-1.408	14630.723
**110**	0	1	0.628	-1.569	-2.776	15817.688
**111**	0	0	2.250	-1.785	-2.989	14185.373
**112**	0	0	1.769	-1.215	-1.270	14161.017
**113**	0	0	0.889	-1.183	-1.502	15416.855
**114**	0	0	2.519	-1.122	-2.479	14058.657
**115**	0	0	2.356	-0.933	-1.718	14824.455
**116**	0	0	2.745	-0.960	-2.058	14082.609
**117**	0	0	3.539	-1.340	-0.555	11369.578
**118**	0	0	2.226	-0.426	0.455	17529.511
**119**	0	0	-1.293	-1.502	-3.668	12199.662
**120**	0	0	3.830	-1.344	1.362	14739.716
**121**	0	0	1.244	-1.362	1.305	18265.655
**122**	1	1	-0.552	-1.466	-1.541	13016.675
**123**	1	1	-0.579	-1.376	-2.060	13694.258
**124**	1	1	-0.887	-1.482	-1.176	13478.120
**125**	1	1	-1.266	-1.341	-1.736	13606.876
**126**	1	1	-0.746	-1.367	-1.607	13541.772
**127**	1	1	-1.475	-1.348	-3.166	13630.209
**128**	1	1	-0.924	-1.659	-0.872	13189.645
**129**	1	1	-1.122	-1.595	-0.806	14090.598
**130**	2	1	0.186	-0.904	0.545	15680.558
**131**	1	1	-1.191	-1.795	-2.590	10924.157
**132**	1	1	-1.236	-1.725	-2.104	10923.306
**133**	1	1	-1.535	-1.583	-2.508	10917.311
**134**	1	1	-1.338	-1.246	-3.444	10918.804
**135**	1	1	-1.375	-1.332	-0.648	10918.804
**136**	0	1	-1.605	-1.744	-4.112	9628.535
**137**	0	0	1.714	-1.370	-4.420	10616.460
**138**	1	1	-1.832	-1.702	-1.253	10694.968
**139**	1	1	-1.793	-1.729	1.290	10704.129

**140**	1	1	-1.850	-1.765	-1.108	10696.301
**141**	1	1	-1.571	-1.504	0.497	10704.128
**142**	0	0	-1.078	-0.798	-2.488	10614.837
**143**	0	0	5.295	0.680	-0.040	15172.831
**144**	0	0	0.560	0.585	-0.525	15775.276
**145**	0	0	3.433	0.920	0.060	16341.484
**146**	0	0	-0.412	1.419	-1.550	16286.350
**147**	0	0	3.087	0.295	0.245	15643.198
**148**	0	0	-1.306	-0.461	-2.751	11247.069
**149**	0	0	-1.896	-1.433	-1.484	11361.414
**150**	0	0	-1.900	-0.796	-2.378	11540.710
**151**	0	0	-0.977	-0.673	-2.363	10995.990
**152**	0	0	4.896	0.774	-0.722	15497.619
**153**	0	1	-1.711	-1.168	-2.643	14895.553
**154**	0	1	-1.603	-1.166	0.327	15554.030
**155**	0	1	-2.250	-1.086	-2.702	15498.627
**156**	0	1	-1.871	-1.067	-0.085	14961.358
**157**	0	1	-1.671	-1.182	-2.905	14822.476
**158**	0	1	-1.085	-2.009	0.285	10067.953
**159**	1	1	-1.788	-1.717	-1.809	11666.624
**160**	1	1	-1.774	-1.571	-1.923	11564.100
**161**	1	1	-1.085	-1.240	-1.257	15337.065
**162**	1	1	-1.585	-1.752	-2.040	11710.334
**163**	1	1	-1.604	-1.476	-0.226	11586.318
**164**	1	1	-1.725	-1.828	-1.079	11015.382
**165**	1	1	-1.471	-1.561	0.188	14047.617
**166**	1	1	-1.269	-1.497	-0.434	13688.571
**167**	1	1	-1.229	-1.494	0.510	14125.127
**168**	1	1	-1.849	-1.344	-1.350	14141.500
**169**	1	1	-1.289	-1.497	-0.078	13660.717
**170**	1	1	-1.247	-1.495	0.720	14083.651
**171**	1	1	-1.857	-1.347	-1.217	14101.207
**172**	1	1	-1.611	-1.444	0.562	13575.192
**173**	1	1	-1.341	-1.500	1.058	13510.718
**174**	1	1	-1.566	-1.496	0.845	12774.773
**175**	0	0	1.551	-1.669	-3.294	10249.534
**176**	0	0	0.001	-1.960	-0.558	10426.094
**177**	1	1	-0.153	-1.383	-2.847	10458.401
**178**	1	1	-1.553	-1.267	-5.268	10533.181
**179**	0	0	1.268	-1.358	-3.523	10980.540
**180**	0	0	1.404	-1.165	-3.005	11000.013

**181**	0	0	-0.295	-1.424	-3.171	11310.044
**182**	0	0	-1.437	-1.534	-5.069	11728.993
**183**	0	0	-1.030	-1.670	-3.440	11481.003
**184**	0	0	-0.126	-1.131	-2.126	12033.028
**185**	0	0	-1.437	-1.151	-3.979	12446.730
**186**	0	0	-0.906	-1.340	-2.824	12202.023
**187**	0	0	-0.161	-1.135	-2.160	12043.199
**188**	0	0	-1.444	-1.190	-3.943	12459.355
**189**	0	0	-0.933	-1.349	-2.754	12213.320
**190**	0	0	-0.922	-1.817	-1.026	11483.303
**191**	3	1	-1.851	-1.714	-2.089	11954.476
**192**	3	1	-1.960	-1.766	-0.125	11814.965
**193**	3	1	-0.910	-1.259	-1.113	14950.935
**194**	3	1	-0.897	-1.289	-0.690	14787.511
**195**	3	1	-0.904	-1.258	-2.242	14546.030
**196**	3	1	-1.144	-1.386	-2.334	14342.728
**197**	3	1	-1.197	-1.407	-0.561	14173.887
**198**	3	1	-1.448	-1.235	-1.585	14463.791
**199**	3	1	-0.917	-1.355	-2.625	13909.987
**200**	3	1	-0.965	-1.380	-0.816	13736.730
**201**	3	1	-1.166	-1.569	-1.981	14073.395
**202**	1	1	-1.412	-1.423	0.441	13878.897
**203**	1	1	2.508	-1.418	0.071	14357.454
**204**	1	1	-1.587	-1.428	1.706	13299.283
**205**	1	1	-1.391	-1.416	0.202	13920.235
**206**	1	1	-1.503	-1.420	-0.275	13478.794
**207**	1	1	-1.581	-1.426	0.930	13322.109
**208**	1	1	-0.928	-1.293	-0.013	16516.650
**209**	1	1	-1.180	-1.209	-0.132	16624.801
**210**	2	1	-1.309	-1.946	-1.200	10699.968
**211**	1	1	-1.067	-1.761	1.966	10974.098
**212**	1	1	-1.546	-1.866	-1.893	11391.144
**213**	1	1	-1.503	-1.826	-2.421	11457.450
**214**	1	1	-1.758	-1.677	-3.289	11396.658
**215**	1	1	-1.499	-1.913	-1.727	11618.522
**216**	1	1	-1.531	-1.699	-1.846	11329.871
**217**	0	1	-1.226	-1.767	3.056	13595.912
**218**	1	1	3.308	-1.173	1.527	17325.021
**219**	2	1	-1.512	-1.700	-1.098	10744.198
**220**	1	1	-1.374	-1.948	-2.318	10507.910
**221**	2	1	-1.309	-1.946	-1.200	10699.968

Table S5. Experimental and predicted values of antichagas activity. Residuals and influence values *h* (*h*^lim = 0.196) are reported. ^ validation group and * test group.

ID	Experimental activity	Predicted activity	Residuals	*h*
**69**	2.614	2.913	-0.300	0.034
**70**	2.503	2.618	-0.116	0.019
**71**	2.254	2.582	-0.328	0.026
**72**	1.037	1.819	-0.781	0.183
**73^**	1.661	2.000	-0.339	0.113
**74^**	1.701	1.986	-0.285	0.028
**75**	1.907	1.347	0.561	0.024
**76^**	0.833	1.268	-0.436	0.021
**77**	0.914	1.292	-0.378	0.023
**78**	1.238	1.887	-0.649	0.020
**79**	1.318	1.907	-0.589	0.023
**80***	2.555	2.143	0.413	0.032
**81**	1.338	1.642	-0.304	0.037
**82^**	1.598	1.865	-0.268	0.022
**83**	1.396	1.909	-0.512	0.022
**84**	1.641	1.872	-0.231	0.016
**85**	1.775	2.170	-0.395	0.036
**86**	2.454	1.919	0.535	0.023
**87**	2.721	2.557	0.164	0.015
**88***	1.745	1.950	-0.205	0.021
**89^**	2.194	1.932	0.262	0.023
**90**	2.429	2.587	-0.159	0.020
**91**	1.674	2.526	-0.852	0.017
**92**	2.747	2.839	-0.092	0.019
**93**	2.395	2.615	-0.220	0.022
**94**	1.369	2.459	-1.090	0.017
**95***	2.496	2.570	-0.074	0.073
**96^**	2.643	2.152	0.491	0.052
**97**	2.342	2.625	-0.283	0.105
**98**	2.026	2.400	-0.374	0.052
**99**	2.368	1.873	0.495	0.028
**100**	-0.229	-0.173	-0.056	0.086
**101**	-0.658	-0.100	-0.558	0.043
**102**	1.623	0.954	0.669	0.085
**103**	1.394	0.822	0.572	0.103
**104**	-0.004	0.777	-0.781	0.110
**105**	0.549	0.611	-0.062	0.091

**106**	0.879	0.373	0.506	0.101
**107^**	0.130	0.666	-0.536	0.097
**108***	0.609	0.483	0.126	0.112
**109***	0.068	-0.064	0.132	0.055
**110**	0.210	0.658	-0.449	0.080
**111**	-0.310	-0.590	0.280	0.084
**112^**	-0.252	0.333	-0.584	0.045
**113**	0.207	0.175	0.031	0.040
**114**	0.140	0.131	0.009	0.059
**115**	0.352	0.331	0.021	0.048
**116**	0.861	0.337	0.524	0.059
**117^**	0.253	0.689	-0.436	0.114
**118**	0.093	0.743	-0.650	0.074
**119**	0.780	0.268	0.512	0.065
**120^**	-0.721	0.405	-1.126	0.155
**121**	0.342	-0.014	0.356	0.115
**122**	1.748	1.992	-0.244	0.021
**123**	1.305	1.855	-0.550	0.024
**124**	1.711	1.996	-0.286	0.016
**125***	2.084	2.036	0.048	0.018
**126**	1.989	1.995	-0.006	0.019
**127**	2.121	1.770	0.351	0.036
**128^**	2.090	1.945	0.145	0.018
**129***	2.837	1.872	0.966	0.016
**130**	2.929	2.724	0.205	0.051
**131**	1.797	1.929	-0.132	0.031
**132***	2.207	2.094	0.113	0.025
**133^**	1.549	2.181	-0.632	0.026
**134**	2.520	2.299	0.221	0.048
**135**	2.945	2.760	0.184	0.015
**136**	2.593	1.600	0.993	0.084
**137**	1.090	0.238	0.852	0.078
**138**	2.558	2.382	0.176	0.019
**139***	2.598	2.841	-0.243	0.045
**140**	2.568	2.351	0.217	0.020
**141**	2.313	2.878	-0.565	0.024
**142**	1.619	1.431	0.188	0.042
**143^**	1.037	1.819	-0.781	0.183
**144**	1.661	2.000	-0.339	0.113
**145**	2.692	2.036	0.656	0.143
**146**	3.302	2.593	0.710	0.264

**147***	2.053	1.645	0.409	0.090
**148**	1.248	1.604	-0.356	0.063
**149**	1.904	0.969	0.935	0.077
**150**	1.830	1.365	0.465	0.073
**151**	1.714	1.492	0.222	0.043
**152**	1.623	1.756	-0.133	0.175
**153**	1.318	1.468	-0.150	0.060
**154^**	2.006	1.910	0.096	0.059
**155**	1.338	1.476	-0.138	0.073
**156**	2.555	2.061	0.495	0.057
**157***	1.598	1.413	0.184	0.062
**158**	3.107	2.065	1.043	0.070
**159**	2.182	2.076	0.105	0.019
**160**	2.542	2.210	0.332	0.019
**161***	1.238	1.887	-0.649	0.020
**162**	1.762	1.970	-0.208	0.021
**163**	2.409	2.605	-0.196	0.017
**164**	2.327	2.226	0.101	0.021
**165**	3.245	2.138	1.106	0.024
**166**	2.522	2.125	0.397	0.017
**167**	2.149	2.225	-0.076	0.026
**168**	1.936	2.068	-0.132	0.020
**169***	2.585	2.200	0.384	0.019
**170***	2.979	2.274	0.705	0.030
**171^**	2.933	2.098	0.835	0.020
**172**	1.945	2.422	-0.477	0.028
**173***	3.401	2.450	0.951	0.035
**174***	2.534	2.570	-0.036	0.031
**175**	-0.102	0.257	-0.359	0.071
**176**	0.781	0.632	0.149	0.083
**177**	2.751	2.250	0.501	0.053
**178**	1.799	2.019	-0.220	0.104
**179**	0.137	0.400	-0.263	0.050
**180**	0.336	0.664	-0.328	0.040
**181^**	0.814	0.501	0.313	0.041
**182**	-0.796	0.069	-0.865	0.088
**183**	0.009	0.259	-0.250	0.060
**184**	0.332	0.829	-0.496	0.030
**185^**	0.386	0.508	-0.123	0.071
**186***	0.097	0.544	-0.447	0.048
**187***	0.401	0.820	-0.419	0.031

**188**	-0.071	0.476	-0.547	0.071
**189**	0.149	0.550	-0.401	0.048
**190**	0.140	0.575	-0.435	0.075
**191***	3.335	2.706	0.629	0.083
**192**	3.231	3.073	0.158	0.086
**193**	2.340	2.678	-0.338	0.079
**194**	3.569	2.760	0.810	0.078
**195**	1.430	2.535	-1.105	0.088
**196**	1.371	2.457	-1.086	0.085
**197^**	3.448	2.816	0.632	0.077
**198**	3.521	2.752	0.768	0.084
**199**	3.225	2.488	0.737	0.091
**200***	3.260	2.850	0.410	0.077
**201***	2.916	2.405	0.511	0.078
**202**	2.960	2.343	0.617	0.025
**203***	2.573	1.794	0.779	0.119
**204**	3.168	2.706	0.462	0.048
**205^**	3.039	2.293	0.746	0.022
**206***	2.644	2.290	0.354	0.018
**207^**	1.951	2.554	-0.603	0.033
**208**	2.896	1.843	1.053	0.026
**209^**	2.362	1.904	0.458	0.028
**210**	2.547	2.472	0.075	0.039
**211**	2.600	2.818	-0.218	0.062
**212**	2.329	1.946	0.383	0.023
**213**	1.952	1.866	0.086	0.026
**214**	1.723	1.875	-0.152	0.036
**215^**	1.743	1.887	-0.144	0.023
**216**	1.938	2.123	-0.185	0.020
**217**	2.052	2.192	-0.140	0.127
**218**	2.097	1.680	0.417	0.163
**219**	2.217	2.736	-0.519	0.031
**220**	2.624	1.932	0.692	0.032
**221^**	2.519	2.472	0.048	0.039

Table S6. Values predicted by the QSAR model of anti-Chagas activity of the 68 naphthoquinone derivatives (prediction group).

ID	Predicted activity	$h^* = 0.196$
1	1.808	0.041
2	1.551	0.064
3	1.721	0.068
4	1.844	0.069
5	1.161	0.063
6	1.415	0.068
7	1.594	0.070
8	1.830	0.059
9	1.922	0.055
10	2.020	0.056
11	1.618	0.061
12	1.687	0.056
13	1.743	0.054
14	1.959	0.126
15	2.036	0.118
16	2.078	0.115
17	2.346	0.299
18	1.515	0.040
19	1.623	0.042
20	1.664	0.043
21	1.255	0.051
22	1.409	0.052
23	1.469	0.052
24	0.955	0.072
25	1.096	0.072
26	1.150	0.071
27	2.164	0.055
28	1.717	0.076
29	1.878	0.081
30	1.993	0.085
31	1.276	0.068
32	1.506	0.076
33	1.672	0.081
34	2.113	0.074
35	2.203	0.074
36	2.297	0.078
37	1.911	0.067

**38**	1.975	0.064
**39**	2.025	0.064
**40**	2.203	0.149
**41**	2.309	0.157
**42**	2.321	0.155
**43**	2.508	0.319
**44**	1.658	0.084
**45**	1.143	0.109
**46**	1.264	0.111
**47**	1.306	0.111
**48**	1.78	0.064
**49**	1.889	0.067
**50**	1.93	0.067
**51**	1.463	0.082
**52**	1.605	0.084
**53**	3.399	0.810
**54**	4.101	0.871
**55**	1.739	0.375
**56**	2.430	0.400
**57**	3.189	0.598
**58**	3.309	0.616
**59**	1.621	0.268
**60**	1.707	0.270
**61**	0.727	0.169
**62**	0.785	0.167
**63**	1.690	0.054
**64**	1.981	0.085
**65**	1.913	0.085
**66**	1.848	0.088
**67**	1.924	0.127
**68**	2.003	0.128

Table S7. Intercorrelation matrix of the QSAR model descriptors for antileishmanial activity.

**QSAR model for anti-*L. amazonensis***					
**Descriptor**	b_1	b_2	b_3	b_4	**VIF**
b_1	1	0.00277	0.02	0.14	1.04
b_2		1	0.000131	0.14	1.05
b_3			1	0.19	1.05
b_4				1	1.25

**QSAR model for anti-*L infantum***							
**Descriptor**	c_1	c_2	c_3	c_4	c_5	c_6	**VIF**
c_1	1	0.01	0.02	0.03	0.03	0.01	1.00
c_2		1	0.03	0.04	0.09	0.03	1.03
c_3			1	0.27	0.21	0.15	1.45
c_4				1	0.79	0.05	3.34
c_5					1	0.04	2.87
c_6						1	1.25

Table S8. Numerical values of the QSAR anti-*L. amazonensis* model descriptors.

ID	b_1	b_2	b_3	b_4
**1**	0	0	4.12	16.79
**2**	0	0	4.54	17.05
**3**	0	0	4.63	17.61
**4**	0	0	4.48	17.99
**5**	0	0	4.65	18.55
**6**	0	0	4.74	18.8
**7**	0	0	4.59	18.9
**8**	0	0	4.37	17.05
**9**	0	0	4.48	17.61
**10**	0	0	4.37	17.99
**11**	0	0	4.37	17.05
**12**	0	0	4.48	17.61
**13**	0	0	4.37	17.99
**14**	0	0	4.44	17.05
**15**	0	0	4.6	17.61
**16**	0	0	4.38	17.99
**17**	0	0	4.68	17.37
**18**	0	0	4.42	17.05
**19**	0	0	4.57	17.61
**20**	0	0	4.35	17.99
**21**	0	0	4.54	19.11
**22**	0	0	4.68	19.29
**23**	0	0	4.54	19.34

**24**	0	0	4.43	18.65
**25**	0	0	4.56	19.19
**26**	0	0	4.43	19.52
**27**	0	0	4.07	16.79
**28**	0	0	4.48	17.05
**29**	0	0	4.57	17.61
**30**	0	0	4.43	17.99
**31**	0	0	4.6	18.55
**32**	0	0	4.68	18.8
**33**	0	0	4.55	18.9
**34**	0	0	4.31	17.05
**35**	0	0	4.41	17.61
**36**	0	0	4.31	17.99
**37**	0	0	4.31	17.05
**38**	0	0	4.41	17.61
**39**	0	0	4.31	17.99
**40**	0	0	4.38	17.05
**41**	0	0	4.54	17.61
**42**	0	6.02	4.32	17.99
**43**	0	6.36	4.61	17.37
**44**	0	0	4.49	19.34
**45**	0	0	4.39	18.65
**46**	0	0	4.52	19.19
**47**	0	0	4.39	19.52
**48**	0	0	4.36	17.05
**49**	0	0	4.51	17.61
**50**	0	0	4.31	17.99
**51**	0	0	4.49	19.11
**52**	0	0	4.63	19.29
**53**	0	0	4.27	17.21
**54**	0	0	4.29	18.03
**55**	0	0	4.4	18.96
**56**	0	0	4.42	19.36
**57**	0	0	4.13	17.21
**58**	0	0	4.22	18.03
**59**	0	0	4.34	19.6
**60**	0	0	4.36	19.93
**61**	0	0	4.23	18.48
**62**	0	0	4.25	19.33
**63**	0	0	4.63	18.37
**64**	0	5.15	4.82	19.85

**65**	0	0	4.91	19.65
**66**	0	0	4.82	19.33
**67**	0	5.32	4.93	20.04
**68**	0	5.36	4.98	20.02
**222**	0	0	4.26	21.34
**223**	0	0	4.37	18.94
**224**	0	0	4.19	15.2
**225**	0	0	4.15	15.2
**69**	0	0	4.14	14.32
**226**	0	0	4.93	20.93
**227**	0	0	4.96	19.47
**228**	0	0	5	20.76
**229**	0	0	3.79	4.75
**230**	0	0	3.69	4.75
**231**	0	0	3.75	6.45
**232**	0	0	4.21	9.82
**233**	0	0	4.16	12.3
**234**	1	0	4.61	26.8
**235**	1	0	4.67	23.65
**236**	1	0	4.84	23.5
**237**	1	0	4.88	24.57
**238**	1	0	4.95	27.33
**239**	1	0	4.95	26.92
**240**	1	0	4.67	23.69
**241**	0	0	5.01	26.03
**242**	0	0	5.16	25.39
**243**	0	0	5.16	25.52
**244**	0	0	5.05	24.26
**245**	0	0	5.18	21.58
**246**	0	0	5.12	23.06
**247**	0	0	3.17	11.11
**248**	0	0	1.92	5.66
**249**	0	0	3.83	11.11
**250**	0	0	3.32	10.48
**251**	0	0	3.97	9.35
**252**	0	0	4.57	10.14
**253**	0	0	4.47	15.81
**254**	0	0	4.79	14.18
**255**	0	0	4.77	14.18
**256**	0	0	4.98	15.66
**257**	0	0	4.98	15.66

**258**	0	0	4.67	15.66
**259**	0	0	4.6	15.66
**260**	0	0	5.02	16.61
**261**	0	0	3.9	25.71
**262**	0	0	4.44	34.38
**263**	1	0	4.28	31.45
**264**	0	0	4.19	39.51
**265**	0	6.8	4.44	39.7
**266**	0	3.8	4.5	39.7
**267**	0	0	4.39	39.7
**268**	0	0	4.9	21.29
**269**	0	0	4.81	21.29
**270**	0	0	5.05	15.3
**271**	0	0	4.92	21.31
**272**	0	0	4.47	6.98
**273**	0	0	3.94	5.14
**274**	0	0	4.18	7.38
**275**	0	0	4.64	9.07
**276**	0	0	4.05	7.38
**277**	0	0	3.83	5.14
**278**	0	0	4.05	7.38
**279**	0	0.39	4.47	11.45
**280**	0	0	4.53	11.8

Table S9. Numerical values of the QSAR anti-*L. infantum* model descriptors.

ID	c_1	c_2	c_3	c_4	c_5	c_6
**1**	0.06	0	-3.44E+05	58.58	0.43	-6.95E+04
**2**	0.19	0	-3.47E+05	53.25	0.25	-1.19E+04
**3**	0.01	0	-3.42E+05	53.25	0.25	-2.34E+04
**4**	0.04	0	-3.41E+05	53.25	0.25	-2.65E+04
**5**	0.16	0	-3.35E+05	55.44	0.48	-9.55E+03
**6**	-0.02	0	-3.29E+05	55.44	0.48	-2.12E+04
**7**	0.02	0	-3.28E+05	55.44	0.48	-2.40E+04
**8**	0.16	0	-3.34E+05	55.79	0.41	-1.25E+05
**9**	-0.01	0	-3.33E+05	55.79	0.41	-1.19E+05
**10**	0.04	0	-3.33E+05	55.79	0.41	-1.17E+05
**11**	0.14	0	-3.24E+05	55.79	0.41	-2.00E+05
**12**	0.01	0	-3.25E+05	55.79	0.41	-1.82E+05
**13**	0.05	0	-3.26E+05	55.79	0.41	-1.77E+05
**14**	0.08	0	-3.64E+05	50.94	0.38	1.15E+05
**15**	0.01	0	-3.56E+05	50.94	0.37	5.74E+04
**16**	0.06	0	-3.54E+05	50.94	0.37	5.08E+04
**17**	0.07	0	-3.79E+05	45.06	0.33	2.71E+05
**18**	0.09	0	-3.58E+05	55.79	0.41	-5.00E+04
**19**	0.04	0	-3.53E+05	55.79	0.4	-5.84E+04
**20**	0.05	0	-3.52E+05	55.79	0.4	-5.87E+04
**21**	0.09	0	-3.48E+05	53.25	0.42	-4.61E+04
**22**	0.05	0	-3.43E+05	53.25	0.42	-5.39E+04
**23**	0.05	0	-3.42E+05	53.25	0.42	-5.49E+04
**24**	0.07	0	-3.48E+05	50.94	0.42	-4.02E+04
**25**	0.04	0	-3.41E+05	50.94	0.42	-4.79E+04
**26**	0.05	0	-3.39E+05	50.94	0.42	-4.96E+04
**27**	0.16	0	-3.97E+05	102.39	0.38	3.73E+05
**28**	0.25	0	-3.96E+05	93.08	0.2	4.01E+05
**29**	0.1	1	-3.90E+05	93.08	0.2	3.84E+05
**30**	0.13	0	-3.89E+05	93.08	0.2	3.79E+05
**31**	0.22	0	-3.81E+05	93.54	0.43	3.85E+05
**32**	0.07	1	-3.76E+05	93.54	0.43	3.68E+05
**33**	0.1	0	-3.74E+05	93.54	0.43	3.64E+05
**34**	0.23	0	-3.85E+05	97.51	0.36	2.88E+05
**35**	0.09	1	-3.84E+05	97.51	0.36	2.96E+05
**36**	0.13	0	-3.84E+05	97.51	0.36	2.99E+05
**37**	0.23	0	-3.75E+05	97.51	0.36	2.03E+05
**38**	0.12	1	-3.76E+05	97.51	0.36	2.27E+05
**39**	0.15	0	-3.77E+05	97.51	0.36	2.34E+05

**40**	0.17	1	-4.12E+05	89.03	0.33	5.13E+05
**41**	0.11	0	-4.03E+05	89.03	0.33	4.57E+05
**42**	0.15	0	-4.00E+05	89.03	0.33	4.43E+05
**43**	0.15	1	-4.21E+05	78.76	0.29	6.30E+05
**44**	0.16	0	-3.90E+05	93.08	0.37	3.49E+05
**45**	0.17	0	-3.95E+05	89.03	0.38	3.56E+05
**46**	0.14	0	-3.87E+05	89.03	0.38	3.42E+05
**47**	0.15	0	-3.85E+05	89.03	0.38	3.38E+05
**48**	0.19	0	-4.09E+05	97.51	0.36	3.77E+05
**49**	0.15	0	-4.04E+05	97.51	0.35	3.67E+05
**50**	0.16	0	-4.02E+05	97.51	0.35	3.64E+05
**51**	0.18	0	-3.97E+05	93.08	0.38	3.63E+05
**52**	0.15	0	-3.92E+05	93.08	0.37	3.52E+05
**53**	0.11	0	-4.96E+05	13.15	-0.02	-2.47E+05
**54**	-2.23E-03	0	-4.87E+05	13.15	-0.02	-2.51E+05
**55**	0.08	0	-4.77E+05	17.09	0.22	-2.37E+05
**56**	-0.02	0	-4.68E+05	17.09	0.22	-2.40E+05
**57**	0.08	0	-5.12E+05	13.78	0.13	-3.10E+05
**58**	0.04	0	-5.05E+05	13.78	0.12	-3.01E+05
**59**	0.07	0	-4.96E+05	13.15	0.16	-2.98E+05
**60**	0.05	0	-4.88E+05	13.15	0.15	-2.88E+05
**61**	0.06	0	-4.91E+05	12.58	0.17	-2.95E+05
**62**	0.04	0	-4.80E+05	12.58	0.16	-2.82E+05
**63**	0.08	0	-3.43E+05	50.62	0.35	4.42E+03
**64**	0.07	0	-3.35E+05	48.75	0.33	-4.26E+04
**65**	0.03	0	-3.35E+05	48.75	0.33	-4.51E+04
**66**	0.12	1	-3.37E+05	48.75	0.33	-5.14E+04
**67**	0.06	0	-3.26E+05	47.01	0.32	-9.51E+04
**68**	0.04	1	-3.28E+05	47.01	0.32	-9.56E+04
**281**	-0.06	0	-2.84E+05	69.68	0.52	-3.13E+05
**282**	-0.01	0	-3.28E+05	80.16	0.32	1.01E+05
**283**	-0.06	0	-4.03E+05	72.99	0.45	3.40E+04
**284**	-0.08	0	-3.64E+05	69.68	0.55	-2.95E+05
**285**	0	0	-3.96E+05	100.64	0.58	3.80E+04
**286**	0.07	0	-2.72E+05	80.79	0.47	2.48E+04
**222**	-0.04	0	-2.42E+05	66.83	0.34	2.49E+04
**223**	-2.75E-04	0	-3.36E+05	44.39	-0.04	2.22E+04
**287**	0.02	0	-3.19E+05	64.19	0.34	2.98E+04
**288**	-0.02	0	-3.16E+05	84.24	0.33	8.64E+03
**224**	-9.44E-04	0	-3.05E+05	53.74	0.09	2.39E+04
**225**	0.03	0	-3.10E+05	49.91	0.21	2.35E+04

**289**	0.03	0	-3.72E+05	83.68	0.3	1.52E+05
**69**	4.68E-03	0	-3.23E+05	80.43	0.29	3.38E+04
**290**	-0.09	0	-3.71E+05	54.75	0.34	3.97E+04
**291**	-0.01	0	-4.15E+05	30.66	0.2	2.35E+04
**292**	-0.13	0	-3.71E+05	47.9	0.27	4.28E+04
**294**	4.57E-03	1	-4.84E+05	6.15	0.1	1.50E+05
**293**	-0.12	0	-3.53E+05	42.58	0.27	3.97E+04
**295**	-1.09E-03	0	-4.55E+05	36.56	0.25	1.55E+05
**296**	-0.14	0	-4.66E+05	38.32	-0.06	1.79E+05
**297**	-0.06	4	-3.29E+05	68.66	0.47	8.73E+04
**298**	-0.06	4	-3.29E+05	68.66	0.47	8.73E+04
**299**	0.1	0	-3.62E+05	54.09	0.53	1.37E+05
**300**	-0.08	2	-3.94E+05	58.96	0.38	4.54E+04
**301**	0.09	2	-3.85E+05	34.84	0.17	8.12E+04
**302**	0.01	0	-3.23E+05	82.46	0.4	-1.30E+05
**303**	0.01	0	-3.20E+05	75.29	0.22	-1.09E+05
**304**	0.01	0	-3.06E+05	78.71	0.38	-2.03E+05
**305**	0.01	0	-3.13E+05	78.71	0.38	-1.64E+05
**306**	-0.01	0	-3.20E+05	61.85	0.31	-2.07E+05
**307**	0.02	0	-3.32E+05	72.15	0.35	-6.05E+04
**308**	-3.21E-04	0	-3.19E+05	78.71	0.38	-1.26E+05
**309**	-0.01	0	-3.20E+05	61.85	0.31	-2.07E+05
**310**	-0.01	0	-3.25E+05	61.85	0.31	-1.76E+05
**311**	-0.01	0	-3.20E+05	59.71	0.27	-2.08E+05
**312**	-0.01	0	-3.39E+05	61.85	0.31	-1.56E+05
**313**	0	0	-3.30E+05	59.71	0.19	-1.32E+05
**314**	-0.01	0	-3.30E+05	61.85	0.31	-1.46E+05
**315**	1.86E-03	0	-3.40E+05	57.72	0.29	-9.28E+04
**316**	-0.01	0	-3.39E+05	61.85	0.31	-1.56E+05
**317**	-0.01	0	-3.33E+05	64.14	0.32	-1.50E+05
**318**	0.01	0	-3.41E+05	61.85	0.31	-1.67E+05
**319**	-0.01	0	-3.79E+05	55.86	0.28	-1.62E+05
**320**	0.01	0	-3.77E+05	46.8	0.23	-1.03E+05
**321**	0.01	0	-3.60E+05	46.8	0.24	-1.63E+05
**322**	0.01	0	-3.65E+05	46.8	0.24	-1.30E+05
**323**	0.01	0	-3.74E+05	48.1	0.24	-1.05E+05
**324**	0.01	0	-3.61E+05	45.57	0.21	-1.58E+05
**325**	0.02	0	-3.77E+05	44.4	0.22	-3.76E+04
**326**	0.02	0	-3.70E+05	45.57	0.15	-8.33E+04
**327**	4.63E-03	0	-3.78E+05	46.8	0.23	-1.04E+05
**234**	0.01	0	-3.82E+05	46.8	0.23	-1.07E+05

**235**	0.03	0	-4.33E+05	51.34	0.36	1.10E+05
**236**	-2.01E-03	0	-4.31E+05	55.85	0.37	1.58E+05
**237**	-0.01	0	-4.09E+05	53.52	0.36	1.64E+04
**238**	-0.03	0	-4.39E+05	49.4	0.27	2.38E+05
**239**	0.03	0	-4.26E+05	47.23	0.21	1.36E+05
**327**	0.02	0	-4.26E+05	47.23	0.21	1.37E+05
**329**	-0.07	0	-4.30E+05	60.35	0.4	1.69E+05
**330**	-0.05	0	-5.06E+05	0	-0.18	1.31E+05
**331**	0.01	0	-4.82E+05	0	-0.13	1.55E+05
**332**	-0.03	0	-4.70E+05	0	-0.07	4.71E+04
**333**	0.03	0	-3.98E+05	0	-0.19	9.85E+04
**334**	-0.01	0	-3.91E+05	20.28	0.05	9.55E+04
**335**	-0.04	0	-3.77E+05	19.47	0.05	9.17E+04
**336**	0.02	0	-4.18E+05	0	-0.09	8.56E+04
**337**	0.04	0	-4.06E+05	0	-0.09	4.90E+04
**338**	0.04	0	-4.00E+05	0	-0.09	1.26E+04
**339**	-0.07	0	-4.25E+05	0	-0.18	-4.90E+05
**340**	0.01	0	-4.05E+05	0	-0.13	-4.36E+05
**341**	-0.04	0	-3.93E+05	0	-0.07	-5.12E+05
**342**	0.03	0	-3.35E+05	0	-0.19	-3.80E+05
**343**	-0.01	0	-3.30E+05	20.28	0.05	-3.67E+05
**344**	-0.04	0	-3.18E+05	19.47	0.05	-3.53E+05
**345**	0.03	0	-3.64E+05	0	-0.09	-3.25E+05
**346**	0.05	0	-3.54E+05	0	-0.09	-3.47E+05
**347**	0.05	0	-3.59E+05	0	-0.19	-2.83E+05
**348**	0.23	0	-5.61E+05	10.34	0.07	2.65E+05
**349**	0.06	0	-5.34E+05	9.04	-0.14	3.06E+05
**350**	0.06	0	-4.63E+05	37.09	-0.08	3.13E+05
**351**	0.01	0	-3.30E+05	0	0	-3.09E+05
**269**	0.05	0	-3.62E+05	53.81	0.15	1.09E+05
**352**	0.01	2	-2.93E+05	87.73	0.68	1.16E+05
**353**	-0.03	0	-2.81E+05	84.22	0.67	1.07E+05
**354**	0.03	2	-2.70E+05	77.98	0.51	1.06E+05
**355**	0.03	2	-2.84E+05	84.22	0.62	1.12E+05
**356**	0.05	0	-2.80E+05	68.8	0.37	8.85E+04
**357**	0.06	2	-3.25E+05	58.98	0.35	1.17E+05
**358**	0.02	0	-3.14E+05	56.94	0.34	1.10E+05

Table S10. Experimental and predicted values of anti-*L. amazonensis* activity. Residuals and influence values *h* (*h*^lim = 0.3125) are reported. ^ validation group and * test group

ID	Experimental activity	Predicted activity	Residuals	h^{lim} = 0.3125
222	0.62	1.22	-0.6	0.04
223	1.99	2.52	-0.53	0.03
224	1.6	0.99	0.61	0.02
225	1.67	1.25	0.42	0.02
69	1.9	1.85	0.05	0.02
226	0.95	1.13	-0.18	0.04
227	1.2	-0.62	1.82	0.03
228	1.26	-0.31	1.57	0.04
229	1.7	0.22	1.48	0.05
230	1.71	0.37	1.34	0.05
231	1.74	0.66	1.08	0.04
232	1.27	-0.89	2.16	0.03
233	1.55	0.42	1.13	0.02
234	0.02	0.38	-0.36	0.2
235	0.03	-0.14	0.17	0.2
236^	0.06	-0.3	0.36	0.21
237*	0.14	0.05	0.09	0.2
238	-0.3	-0.98	0.68	0.2
239*	-0.4	-1.35	0.95	0.2
240	0.24	0.51	-0.27	0.2
241*	1.3	1.3	0	0.06
242	0.9	-0.91	1.81	0.06
243	1.38	0.6	0.78	0.06
244^	1.36	0.49	0.87	0.12
245	1.48	0.26	1.22	0.04
246	1.45	0.48	0.97	0.04
247	0.7	-1.11	1.81	0.01
248	0.93	0.43	0.5	0.01
249^	0.85	-1.52	2.37	0.02
250	0.78	-1.16	1.94	0.01
251	1.34	-0.43	1.77	0.03
252	1.94	0.78	1.16	0.04
253	1.38	0.02	1.36	0.02
254	1.13	-1.42	2.55	0.03
255	1.67	0.27	1.4	0.03
256	1.52	-0.24	1.76	0.03

**257**	1.88	0.87	1.01	0.03
**258**	2.13	2.06	0.07	0.03
**259**	1.29	-0.45	1.74	0.03
**260**	1.09	-1.49	2.58	0.03
**261**	0.85	0.64	0.21	0.09
**262**	0.72	0.83	-0.11	0.19
**263**	-0.4	0.23	-0.63	0.22
**264**	-0.52	-1.91	1.39	0.32
**265**	1.84	-0.01	1.85	1
**266***	0.89	-0.77	1.66	0.537
**267^**	-0.22	-1.21	0.99	0.31
**268^**	1.51	0.69	0.82	0.04
**269***	1.8	1.71	0.09	0.04
**270**	1.5	-0.46	1.96	0.03
**271**	1.78	1.52	0.26	0.04
**272***	1.45	-1.13	2.58	0.06
**273**	1.38	0.91	0.47	0.06
**274**	1.46	-0.65	2.11	0.05
**275**	1.63	-0.47	2.1	0.05
**276**	1.62	0.65	0.97	0.04
**277**	1.34	-0.9	2.24	0.05
**278^**	1.29	-0.99	2.28	0.04
**279**	1.72	0.12	1.6	0.04
**280**	1.45	-0.48	1.93	0.03

Table S11. *Experimental* and predicted values of anti-L. infantum activity. Residuals and influence values *h* (*h*^lim = 0.24) are reported. ^ validation group and * test group

ID	Experimental activity	Predicted activity	Residuals	$h^{lim} = 0.24$
281^	1.13	0.13	1	0.13
282	1.26	0.03	1.23	0.06
283*	1.38	1.52	-0.14	0.06
284	1.29	0.48	0.81	0.15
285	2.1	1.12	0.98	0.07
286	1.21	0.2	1.01	0.07
222	0.35	0.12	0.23	0.03
223	1.45	-0.33	1.78	0.1
287^	1.65	1.5	0.15	0.03
288	1.82	0.45	1.37	0.07
224	1.41	0.18	1.23	0.06
225	1.59	1.44	0.15	0.02
289	1.74	0.04	1.7	0.09
69	2.13	0.96	1.17	0.07
290	0.08	0.04	0.04	0.07
291	0.46	0.19	0.27	0.04
292	-0.15	0.33	-0.48	0.12
294^	0	0.04	-0.04	0.12
293	-0.15	0.66	-0.81	0.11
295	0.65	1.07	-0.42	0.06
296	-0.1	-1.07	0.97	0.24
297	0.72	-0.89	1.61	0.34
298	1.13	0.34	0.79	0.04
299	0.38	0.46	-0.08	0.18
300	0.43	-0.68	1.11	0.11
301	2.09	1.06	1.03	0.15
302	1.88	-0.29	2.17	0.05
303	1.93	-0.83	2.76	0.09
304*	2.11	-0.05	2.16	0.06
305	2.28	0.61	1.67	0.05
306	0.93	-1.7	2.63	0.04
307	2.28	1.47	0.81	0.03
308	1.32	-1.16	2.48	0.04
309	1.46	-0.51	1.97	0.04
310	1.65	0.14	1.51	0.03
311*	1.86	0.24	1.62	0.04

**312**	1.84	0.66	1.18	0.03
**313**	1.77	-0.2	1.97	0.04
**314***	0.96	-1.15	2.11	0.03
**315**	1.42	0.23	1.19	0.02
**316**	1.44	-0.24	1.68	0.03
**317***	1.49	-0.17	1.66	0.03
**318^**	1.44	-0.64	2.08	0.03
**319**	2.03	0.99	1.04	0.03
**320^**	1.69	0.82	0.87	0.02
**321**	1.71	0.46	1.25	0.03
**322***	1.6	0.44	1.16	0.02
**323**	1.31	-0.09	1.4	0.02
**324***	2.08	1.18	0.9	0.02
**325**	0.91	-0.4	1.31	0.02
**326**	1.75	0.38	1.37	0.02
**327**	1.92	1.33	0.59	0.02
**234**	0.08	-2.93	3.01	0.02
**235**	0	-1.43	1.43	0.06
**236**	0.26	-0.22	0.48	0.06
**237**	0.3	-0.9	1.2	0.04
**238**	0.34	0.74	-0.4	0.07
**239**	0.15	-1.72	1.87	0.03
**327***	0.68	-0.41	1.09	0.03
**329**	-0.3	-0.41	0.11	0.09
**330**	0.73	0.97	-0.24	0.13
**331**	0.56	0.45	0.11	0.09
**332^**	0.38	0.39	-0.01	0.08
**333**	0.49	-0.14	0.63	0.07
**334**	0.34	0.35	-0.01	0.03
**335**	-0.4	-0.8	0.4	0.04
**336**	0.11	-0.39	0.5	0.06
**337***	0.28	-0.5	0.78	0.05
**338**	0.04	-1.23	1.27	0.05
**339^**	2.26	0.26	2	0.17
**340**	1.7	-1.26	2.96	0.12
**341**	2.18	0.58	1.6	0.15
**342***	1.71	-0.91	2.62	0.11
**343**	1.45	-0.48	1.93	0.07
**344**	2.14	1.79	0.35	0.07
**345**	2.29	1.5	0.79	0.08
**346**	2.19	0.86	1.33	0.09

**347**	2.1	0.28	1.82	0.09
**348**	1.28	-0.31	1.59	0.35
**349**	0.57	-0.36	0.93	0.13
**350**	2.07	1.84	0.23	0.15
**351**	0.62	-0.76	1.38	0.08
**269**	0.92	-0.98	1.9	0.05
**352^**	-0.15	-1.93	1.78	0.13
**353**	-0.52	-0.63	0.11	0.12
**354**	0.9	-0.46	1.36	0.11
**355**	1.75	1.78	-0.03	0.12
**356**	-0.1	-2.15	2.05	0.04
**357^**	0.92	-0.89	1.81	0.11
**358**	0.91	1.2	-0.29	0.03

Table S12. Values predicted by the QSAR models of antileishmanial activity of the 68 naphthoquinone derivatives (prediction group).

**ID**	**Predicted** *anti-L. amazonensis activity*	*h**	**Predicted anti-** *L. infantum activity*	*h**
**1**	1.18	0.02	1.13	0.07
**2**	1.34	0.03	2.29	0.2
**3**	1.35	0.03	1.08	0.02
**4**	1.27	0.03	1.33	0.03
**5**	1.32	0.03	1.27	0.21
**6**	1.34	0.03	0.1	0.08
**7**	1.28	0.03	0.35	0.08
**8**	1.27	0.02	1.98	0.2
**9**	1.29	0.03	0.8	0.05
**10**	1.23	0.03	1.11	0.06
**11**	1.27	0.02	2.1	0.18
**12**	1.29	0.03	1.11	0.06
**13**	1.23	0.03	1.36	0.07
**14**	1.3	0.02	0.58	0.08
**15**	1.34	0.03	0.29	0.05
**16**	1.23	0.03	0.63	0.06
**17**	1.39	0.03	-0.05	0.09
**18**	1.29	0.02	1.32	0.09
**19**	1.33	0.03	1.03	0.06
**20**	1.22	0.03	1.06	0.06
**21**	1.24	0.03	1.09	0.1

**22**	1.29	0.03	0.85	0.07
**23**	1.23	0.03	0.89	0.08
**24**	1.22	0.03	0.87	0.1
**25**	1.25	0.03	0.67	0.07
**26**	1.17	0.03	0.73	0.08
**27**	1.16	0.02	2.33	0.31
**28**	1.32	0.03	3.13	0.57
**29**	1.33	0.03	2.41	0.33
**30**	1.25	0.03	2.34	0.33
**31**	1.29	0.03	2.1	0.37
**32**	1.32	0.03	1.42	0.15
**33**	1.26	0.03	1.34	0.17
**34**	1.25	0.02	2.96	0.42
**35**	1.26	0.03	2.26	0.2
**36**	1.2	0.03	2.23	0.23
**37**	1.25	0.02	3.18	0.39
**38**	1.26	0.03	2.63	0.22
**39**	1.2	0.03	2.54	0.24
**40**	1.28	0.02	1.85	0.33
**41**	1.31	0.03	1.35	0.22
**42**	2.67	0.94	1.68	0.27
**43**	2.9	1.09	1.09	0.33
**44**	1.22	0.02	2.64	0.34
**45**	1.19	0.03	1.88	0.27
**46**	1.19	0.03	1.6	0.28
**47**	1.18	0.03	0.87	0.3
**48**	1.27	0.02	2.44	0.24
**49**	1.3	0.03	2.21	0.25
**50**	1.2	0.03	2.25	0.25
**51**	1.22	0.03	2.16	0.21
**52**	1.27	0.03	1.98	0.22
**53**	1.22	0.02	2.64	0.12
**54**	1.19	0.03	1.88	0.07
**55**	1.19	0.03	1.6	0.17
**56**	1.18	0.03	0.87	0.14
**57**	1.16	0.02	2.2	0.16
**58**	1.16	0.03	1.93	0.13
**59**	1.13	0.03	1.94	0.17
**60**	1.13	0.03	1.73	0.14
**61**	1.14	0.03	1.73	0.16
**62**	1.11	0.03	1.56	0.15

**63**	1.32	0.03	1.05	0.07
**64**	2.57	0.68	1.08	0.06
**65**	1.37	0.03	0.79	0.04
**66**	1.35	0.03	1.73	0.12
**67**	2.65	0.73	1.14	0.05
**68**	2.68	0.75	1.31	0.05

Table S13. Intercorrelation matrix of the QSAR model descriptors for toxicity.

Descriptor	d_1	d_2	d_3	d_4	d_5	VIF
d_2	1	4.05E-03	0.03	0.07	5.03E-04	1.04
d_2		1	0.18	2.05E-05	0.10	1.08
d_3			1	0.22	0.44	1.53
d_4				1	0.31	1.28
d_5				0.31	1	1.39

Table S14. Numerical values of the QSAR toxicity model descriptors.

ID	d_1	d_2	d_3	d_4	d_5
**1**	4.44	0.22	1.67	15.60	0.73
**2**	4.67	0.65	1.70	9.26	0.62
**3**	4.79	0.87	1.67	9.43	0.76
**4**	4.80	0.94	1.66	9.50	0.80
**5**	5.01	0.76	1.69	16.83	0.81
**6**	5.13	0.91	1.66	17.23	0.94
**7**	5.15	0.98	1.66	17.31	0.99
**8**	4.83	1.03	1.62	11.13	0.83
**9**	4.87	0.75	1.61	11.73	0.88
**10**	4.87	0.78	1.61	11.83	0.89
**11**	4.68	-0.02	1.68	6.70	0.89
**12**	4.87	-0.21	1.65	7.03	0.91
**13**	4.87	-0.15	1.64	7.08	0.91
**14**	5.14	1.26	1.81	8.70	1.26
**15**	5.18	0.76	1.76	8.57	1.29
**16**	5.19	0.71	1.73	8.55	1.29
**17**	5.94	0.41	1.86	3.85	1.82
**18**	4.79	0.27	1.64	16.33	0.92
**19**	4.89	0.11	1.62	16.27	0.91
**20**	4.87	0.08	1.62	16.32	0.91
**21**	5.22	0.46	1.63	17.06	1.11
**22**	5.32	0.28	1.61	17.00	1.10
**23**	5.30	0.24	1.60	17.05	1.10

**24**	5.64	0.62	1.61	17.65	1.30
**25**	5.73	0.43	1.59	17.73	1.28
**26**	5.72	0.37	1.58	17.77	1.28
**27**	1.88	0.37	1.68	11.18	0.94
**28**	2.18	0.77	1.70	7.18	0.79
**29**	2.27	1.00	1.67	7.28	0.94
**30**	2.28	1.08	1.67	7.18	0.99
**31**	2.50	0.89	1.70	10.52	0.98
**32**	2.59	1.05	1.67	11.15	1.13
**33**	2.60	1.13	1.66	11.29	1.18
**34**	2.25	1.14	1.62	6.58	1.03
**35**	2.28	0.88	1.62	7.05	1.10
**36**	2.28	0.92	1.62	7.09	1.11
**37**	2.15	0.08	1.68	8.76	1.10
**38**	2.28	-0.11	1.65	8.57	1.13
**39**	2.28	-0.05	1.64	8.55	1.13
**40**	2.57	1.40	1.81	9.30	1.50
**41**	0.44	0.83	1.99	11.83	1.56
**42**	0.45	0.78	1.94	11.90	1.55
**43**	3.31	0.50	1.86	3.86	2.07
**44**	1.95	-0.87	3.45	17.08	-0.13
**45**	1.94	-0.77	3.41	16.89	0.01
**46**	2.29	-0.74	3.44	2.21	0.03
**47**	2.28	-0.66	3.40	2.11	0.17
**48**	2.22	0.42	1.64	11.62	1.14
**49**	2.29	0.24	1.63	11.86	1.13
**50**	2.28	0.21	1.63	11.95	1.13
**51**	2.61	0.61	1.63	12.47	1.33
**52**	2.68	0.42	1.61	12.63	1.32
**53**	2.67	0.37	1.61	12.72	1.32
**54**	2.98	0.78	1.61	12.99	1.53
**55**	3.05	0.57	1.59	13.38	1.51
**56**	3.04	0.51	1.58	13.46	1.51
**57**	1.81	-0.50	3.45	2.29	0.39
**58**	1.79	-0.56	3.43	2.68	0.39
**59**	2.19	-0.37	3.44	2.87	0.55
**60**	2.17	-0.43	3.41	2.81	0.55
**61**	2.56	-0.25	3.42	2.97	0.72
**62**	2.54	-0.32	3.37	2.92	0.72
**63**	3.34	-0.09	1.63	6.37	1.53
**64**	3.66	0.12	1.58	5.31	1.69
**65**	3.66	0.10	1.58	5.24	1.69

**66**	3.64	0.35	1.58	4.99	1.68
**67**	3.97	0.09	1.53	3.68	1.84
**68**	3.95	-0.36	1.53	4.00	1.84
**359**	4.95	1.50	1.56	6.16	1.61
**360**	4.94	1.64	1.56	3.95	0.94
**361**	5.32	1.69	1.53	9.09	1.79
**362**	4.83	0.61	1.54	6.31	1.29
**363**	5.75	0.39	1.73	13.73	0.23
**364**	4.88	0.64	1.52	6.38	1.31
**365**	1.22	-0.05	1.41	5.57	-0.01
**366**	1.38	-0.08	1.39	7.96	-0.07
**367**	7.54	1.75	1.50	12.95	1.84
**368**	7.37	0.69	1.40	18.81	1.52
**369**	7.37	1.31	1.42	16.03	1.52
**370**	7.37	0.77	1.38	18.04	1.52
**371**	7.13	1.22	1.41	20.24	1.51
**372**	7.35	1.53	1.36	9.49	1.51
**373**	7.53	1.80	1.52	12.97	1.85
**374**	7.35	0.63	1.41	19.02	1.52
**375**	6.26	0.75	1.96	6.45	1.15
**376**	2.37	1.18	0.98	5.31	0.42
**377**	4.00	0.95	1.34	1.81	1.38
**160**	3.09	0.23	1.33	3.38	0.93
**78**	4.79	1.71	1.36	5.87	2.70
**162**	4.78	1.37	1.32	5.88	1.87
**378**	4.94	0.06	2.93	0.93	-0.66
**379**	5.37	0.31	2.92	0.12	-0.55
**380**	5.75	0.36	2.91	1.37	-0.45
**381**	5.85	0.62	2.89	1.42	-0.45
**382**	6.37	0.98	2.84	1.49	-0.34
**383**	6.14	1.22	2.77	5.50	0.62
**384**	6.22	0.62	2.88	0.17	-0.35
**385**	6.12	0.41	2.88	2.65	-0.35
**386**	6.59	0.65	2.86	1.81	-0.25
**387**	5.36	0.32	2.47	1.59	-0.02
**388**	5.84	0.47	2.82	0.68	-0.45
**389**	6.22	0.61	2.81	0.79	-0.35
**390**	7.20	0.84	2.78	2.29	-0.06
**391**	7.80	0.89	2.24	1.96	-0.04
**392**	7.35	0.61	2.65	1.40	-0.21
**393**	8.77	1.10	2.26	2.24	0.19
**394**	7.69	0.67	2.59	1.59	-0.11

**395**	8.44	0.90	2.52	2.05	0.07
**396**	7.69	0.18	2.57	5.01	-0.08
**397**	7.91	-0.21	2.54	6.85	-0.02
**398**	7.72	0.01	2.57	1.93	-0.11
**399**	8.08	-0.22	2.52	7.79	-0.01
**400**	8.79	-0.02	2.46	6.80	0.05
**401**	9.15	-0.35	2.41	5.45	0.26
**402**	4.46	0.22	3.78	2.90	-0.35
**403**	5.03	-0.34	2.88	0.87	-0.50
**404**	3.86	0.16	3.38	1.50	-0.35
**405**	4.24	-0.27	2.94	1.14	-0.41
**406**	4.69	-0.31	2.82	0.85	-0.27
**407**	4.85	-0.26	2.82	1.00	-0.22
**408**	2.65	-0.90	3.00	0.46	-0.48
**409**	4.04	1.50	1.99	3.33	0.08
**410**	3.57	1.43	2.01	3.78	0.00
**411**	3.63	1.27	2.05	-0.16	-0.09
**412**	4.43	1.71	2.02	0.62	0.23
**413**	4.17	1.56	2.15	3.00	0.18
**414**	3.70	1.48	2.21	1.47	0.01
**415**	3.75	1.34	2.21	0.67	0.02
**416**	4.57	1.77	2.17	2.01	0.33
**191**	3.38	1.61	1.90	-1.25	1.17
**417**	2.97	1.40	1.94	1.96	1.00
**418**	3.76	1.84	1.91	3.80	1.43
**419**	5.53	1.82	1.61	3.77	0.22
**420**	6.45	1.45	1.53	0.72	0.61
**421**	6.20	1.46	1.55	9.30	0.13
**422**	5.49	-0.11	2.62	9.82	1.54
**423**	6.73	1.11	2.25	6.97	2.11
**424**	5.11	-0.57	2.43	10.13	2.62
**425**	5.63	1.81	1.14	3.95	2.17
**426**	5.63	0.40	1.16	11.86	2.18
**427**	5.63	0.03	1.18	12.77	2.19
**428**	5.68	1.73	1.33	11.50	2.58
**429**	5.25	0.51	1.17	1.40	1.99
**430**	6.12	0.34	1.41	1.09	0.91

Table S15. Experimental and predicted values of toxicity. Residuals and influence values *h* (*h*^lim = 0.3125) are reported. ^ validation group and * test group

ID	Experimental activity	Predicted activity	Residuals	$h^{lim} = 0.3125$
**359**	0.70	0.61	0.09	0.05
**360**	0.59	-1.15	1.73	0.04
**361***	1.10	3.96	-2.86	0.06
**362**	0.92	0.65	0.27	0.03
**363**	0.92	0.11	0.81	0.42
**364^**	0.82	0.16	0.66	0.03
**365**	0.72	-0.29	1.00	0.06
**366**	0.83	0.88	-0.05	0.1
**367^**	0.14	-1.72	1.86	0.08
**368**	0.38	0.01	0.37	0.14
**369**	0.36	0.11	0.26	-0.15
**370^**	0.27	-0.72	0.99	0.13
**371**	-0.13	-1.80	1.67	0.19
**372^**	0.51	-0.71	1.22	0.07
**373**	0.66	1.35	-0.69	0.08
**374**	0.58	1.17	-0.59	0.15
**375**	1.14	0.22	0.92	0.02
**376**	0.52	0.57	-0.06	0.04
**377^**	0.98	1.18	-0.20	0.05
**160**	0.79	-0.21	1.00	0.02
**78**	0.38	0.57	-0.19	0.13
**162**	0.45	-0.17	0.62	0.06
**378**	2.07	0.23	1.84	0.06
**379***	2.03	-0.11	2.13	0.05
**380**	1.90	-0.51	2.41	0.05
**381**	1.90	-0.22	2.12	0.05
**382**	1.93	0.31	1.62	0.05
**383**	1.21	-1.23	2.44	0.04
**384^**	2.00	-0.09	2.09	0.05
**385**	2.20	1.51	0.68	0.04
**386***	1.90	-0.29	2.19	0.04
**387**	1.98	1.56	0.43	0.03
**388**	1.60	-2.17	3.77	0.04
**389**	1.93	-0.21	2.13	0.04
**390**	1.90	0.07	1.83	0.04
**391**	2.08	1.86	0.22	0.07
**392***	1.61	-2.11	3.72	0.05
**393**	1.61	0.10	1.51	-0.94

**394**	1.90	-0.27	2.17	0.06
**395**	1.86	-0.27	2.13	0.08
**396**	2.00	0.73	1.27	0.05
**397**	1.83	-0.25	2.07	0.08
**398**	2.12	0.30	1.82	0.08
**399**	1.65	-1.12	2.77	0.08
**400**	1.86	-0.17	2.03	0.09
**401**	2.05	0.24	1.81	0.14
**402**	2.25	0.79	1.46	0.15
**403***	2.24	0.93	1.32	0.07
**404**	2.09	0.63	1.46	0.12
**405***	2.07	0.56	1.51	0.08
**406^**	1.65	-1.90	3.55	0.07
**407**	2.06	0.55	1.51	0.06
**408**	1.70	-1.76	3.46	0.16
**409**	1.11	0.35	0.76	0.06
**410**	0.67	-2.00	2.67	0.07
**411**	1.61	1.96	-0.34	0.05
**412**	1.12	0.07	1.05	-0.28
**413***	0.89	-1.26	2.15	0.07
**414***	0.84	-2.10	2.94	0.07
**415**	1.28	0.01	1.26	0.06
**416**	1.33	1.07	0.26	0.07
**191**	0.74	0.10	0.63	-1.24
**417^**	0.48	-2.14	2.62	0.08
**418**	0.74	0.44	0.30	0.09
**419**	0.82	-0.82	1.64	0.08
**420**	1.43	1.42	0.01	0.08
**421**	0.69	-0.45	1.14	0.11
**422**	1.13	-0.45	1.58	0.11
**423**	1.11	0.72	0.40	0.08
**424**	0.93	-0.37	1.30	0.27
**425**	0.19	-1.55	1.74	0.11
**426**	0.52	0.79	-0.28	0.08
**427**	0.41	-0.04	0.45	0.10
**428**	0.06	-0.34	0.40	0.1
**429**	0.93	0.47	0.46	0.14
**430**	0.98	-1.63	2.61	0.08

Table S16. Values predicted by the QSAR model of toxicity of the 68 naphthoquinone derivatives (prediction group).

ID	Predicted activity	h^{lim}=0.3
**1**	0.55	0.14
**2**	0.81	0.04
**3**	0.74	0.04
**4**	0.71	0.04
**5**	0.43	0.16
**6**	0.36	0.17
**7**	0.33	0.17
**8**	0.60	0.06
**9**	0.62	0.06
**10**	0.61	0.06
**11**	1.02	0.03
**12**	1.05	0.04
**13**	1.03	0.04
**14**	0.70	0.04
**15**	0.78	0.03
**16**	0.79	0.03
**17**	1.11	0.10
**18**	0.49	0.14
**19**	0.54	0.14
**20**	0.54	0.14
**21**	0.43	0.14
**22**	0.48	0.14
**23**	0.48	0.14
**24**	0.37	0.14
**25**	0.41	0.14
**26**	0.42	0.14
**27**	0.42	0.14
**28**	0.59	0.08
**29**	0.51	0.08
**30**	0.49	0.09
**31**	0.41	0.12
**32**	0.32	0.13
**33**	0.28	0.13
**34**	0.48	0.08
**35**	0.50	0.08
**36**	0.49	0.08
**37**	0.59	0.10
**38**	0.64	0.10
**39**	0.63	0.09

**40**	0.32	0.14
**41**	0.14	0.32
**42**	0.13	0.31
**43**	0.76	0.13
**44**	1.23	0.61
**45**	1.18	0.59
**46**	1.92	0.25
**47**	1.87	0.25
**48**	0.38	0.13
**49**	0.40	0.13
**50**	0.41	0.14
**51**	0.30	0.13
**52**	0.33	0.13
**53**	0.34	0.13
**54**	0.24	0.13
**55**	0.26	0.13
**56**	0.27	0.13
**57**	1.75	0.29
**58**	1.74	0.29
**59**	1.71	0.27
**60**	1.71	0.27
**61**	1.68	0.25
**62**	1.68	0.25
**63**	0.78	0.08
**64**	0.77	0.08
**65**	0.78	0.08
**66**	0.74	0.07
**67**	0.85	0.10
**68**	0.92	0.14

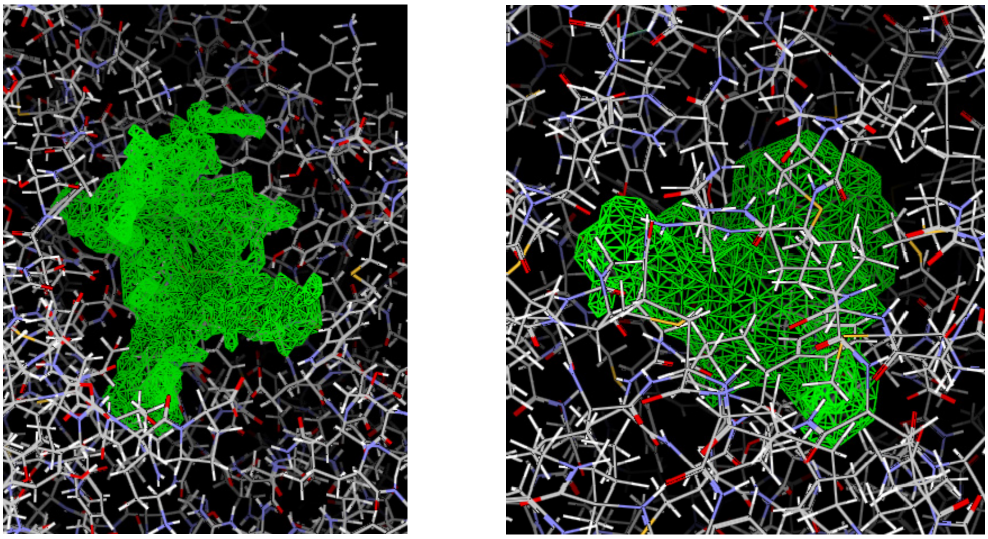

(**a**) (**b**)

Figure S1. Cavities in the active site of *Tc*TR (**a**) and *Tc*LαD (**b**) proteins.

Table S17. Results of the coupling of naphthoquinone derivatives in the active site of the *Tc*TR and *Tc*LαD proteins.

Ligand	*Tc*TR				*Tc*LαD			
	Energy (kcal/mol)	Hydrogen bonds (HB)			Energy (kcal/mol)	Hydrogen bonds (HB)		
		Residues	Dinstance (Å)	Force		Residues	Distance (Å)	Force
**1**	-93.2095	Lys 62 A	3.27	-1.60	-79.966	-		
		Ser 464 B	2.78	-2.50				
**2**	-93.1213	-			-101.396	Tyr 103 B	3.12	-2.4
**3**	-97.3813	Lys 62 A	3.25	-1.75	-102.118	-		
		Ser 464 B	2.69	-2.50				
**4**	-98.8420	Lys 62 A	2.93	-2.50	-89.6888	-		
		Ser 464 B	2.84	-2.50				
		Thr 463 B	3.35	-1.15				
**5**	-96.1777	Asn 340 A	3.12	-2.39	-99.6799	Tyr 103 B	3.34	-1.31
**6**	-108.0890	Lys 62 A	2.94	-2.50	-113.434	-		
		Ser 464 B	2.84	-2.50				
		Thr 463 B	3.32	-1.31				
**7**	-101.9060	Lys 62 A	3.27	-1.67	-93.4699	-		
		Ser 464 B	2.86	-2.50				
**8**	-91.5210	Lys 62 A	2.95	-2.05	-80.5908	-		
		Ser 464 B	2.60	-2.39				
		Thr 463 B	3.30	-1.48				
**9**	-96.9612	Lys 62 A	2.94	-2.50	-89.3261	-		
		Ser 464 B	2.84	-2.50				
		Thr 463 B	3.28	-1.54				
**10**	-94.0496	Lys 62 A	3.28	-1.58	-86.8946	-		
		Ser 464 B	2.78	-2.50				
**11**	-90.5348	Ser 464 B	2.68	-2.50	-94.7798	-		
**12**	-96.7141	Lys 62 A	2.94	-2.50	-97.2161	-		
		Ser 464 B	2.84	-2.50				
		Thr 463 B	3.30	-1.43				
**13**	-95.6654	Lys 62 A	3.19	-1.83	-92.1773	Tyr 116 B	2.77	-2.50
		Ser 464 B	2.82	-2.50		Tyr 103 B	2.76	-2.50
		Thr 463 B	3.37	-1.05				
**14**	-103.9370	Ser 464 B	2.48	-1.54	-108.916	Tyr 116 B	3.03	-2.5
**15**	-100.7810	Lys 62 A	3.20	-1.90	-109.628	Tyr 116 B	3.18	-2.08
		Ser 464 B	2.83	-2.50				
		Thr 463 B	3.29	-1.51				
**16**	-90.6406	Lys 62 A	2.78	-0.94	-90.6885	Tyr 103 B	3.47	-0.65
			3.12	-1.48				

**17**	-111.5950	Ser 464 B	2.99	-0.70	-96.9022	-		
		Leu 399 B	2.48	-1.48				
**18**	-88.1999	Ser 464 B	3.0700	-2.4200	-84.9483	-		
**19**	-93.0333	Lys 62 A	3.24	-1.80	-102.487	-		
		Ser 464 B	2.80	-2.50				
**20**	-87.2394	-			-88.8781	-		
**21**	-102.4510	Lys 62 A	3.37	-1.04	-90.7122	-		
		Ser 464 B	2.72	-2.50				
**22**	-101.7750	Lys 62 A	3.13	-2.37	-94.9361	Tyr 116 B	3.22	-1.89
		Ser 464 B	2.78	-2.50				
**23**	-99.1009	Lys 62 A	3.16	-2.13	-95.958	Tyr 116 B	2.71	-2.50
		Ser 464 B	2.84	-2.50		Tyr 103 B	2.72	-2.50
						HEM_1450 B	0	-0.21
**24**	-102.5440	Ser 464 B	2.65	-2.50	-107.877	-		
**25**	-97.5418	Glu 467 B	3.22	-1.88	-108.501	-		
**26**	-100.7280	Lys 62 A	2.93	-2.50	-98.0511	Tyr 116 B	2.65	-2.5
		Ser 464 B	2.84	-2.50				
		Thr 463 B	3.35	-1.11				
**27**	-87.9757	Lys 62 A	3.27	-1.64	-82.1982	-		
		Ser 464 B	3.77	-2.5				
		Thr 463 B	2.90	-2.5				
**28**	-95.2882	Lys 62 A	2.94	-2.12	-91.0531	Tyr 103 B	3.08	-1.82
		Ser 464 B	2.58	-2.32				
		Thr 463 B	3.22	-1.83				
**29**	-100.6680	Lys 62 A	2.98	-2.5	-95.5983	-		
		Ser 464 B	2.83	-2.5				
		Thr 463 B	3.25	-1.27				
**30**	-98.5385	Lys 62 A	3.12	-1.27	-90.1862	HEM_1450 B	0	-2.5
		Ser 464 B	2.57	-2.22				
		Thr 463 B	3.19	-1.79				
**31**	-91.1649	Ser 464 B	3.28	-1.61	-97.5895	-		
**32**	-99.3677	Gly 459 B	3.11	-2.33	-107.973	-		
		Arg 355 A	2.63	-2.50				
**33**	-101.1350	Lys 62 A	3.18	-1.04	-100.14	Met 360 B	3.04	-1.25
		Ser 464 B	2.77	-2.50		HEM_1450 B	0	-2.5
		Thr 463 B	3.30	-1.02				
**34**	-90.2534	Lys 62 A	3.43	-0.76	-85.9238	-		
		Ser 464 B	2.78	-2.50				
**35**	-95.1599	Lys 62 A	3.03	-2.25	-96.4178	-		
		Ser 464 B	2.79	-2.50				

		Thr 463 B	3.22	-1.42				
**36**	-92.4304	Lys 62 A	3.27	-1.67	-87.0079	HEM_1450 B	0	-2.48
		Ser 464 B	2.62	-2.50				
**37**	-89.9000	Lys 62 A	3.39	-0.93	-90.2266	-		
		Ser 464 B	2.85	-2.50				
**38**	-91.8954	Glu 466 B	2.84	-1.16	-95.5077	Met 360 B	3.18	-0.99
			3.34	-1.30		HEM_1450 B	0	-2.15
			2.64	-2.22		Met 358 B	3.16	-2.18
**39**	-92.2933	Ser 464 B	2.59	-2.38	-86.5687	Tyr 103 B	2.56	-2.17
		Thr 463 B	3.20	-1.66				
**40**	-98.1876	Arg 472 B	2.67	-2.50	-79.3277	Tyr 103 B	2.97	-2.5
			3.29	-1.55				
			3.07	-1.82				
		Tyr 455 B	2.99	-1.53				
		Thr 457 B	2.71	-0.75				
**41**	-98.3294	Lys 62 A	2.97	-2.50	-105.707	Tyr 116 B	3.13	-2.37
		Ser 464 B	2.84	-2.50				
		Thr 463 B	3.26	-1.24				
**42**	-99.5445	Gly 459 B	3.10	-1.17	-92.1493	Tyr 116 B	2.92	-2.50
		His 461 B	2.57	-1.18		Met 358 B	3.39	-1.03
						Tyr 103 B	3.11	-1.09
							2.79	-2.50
**43**	-110.8730	Leu 399 B	2.96	-0.81	-99.1137	-		
		Ser 464 B	2.49	-1.57				
		Thr 463 B	3.06	-2.50				
**44**	-87.7661	Gly 459 B	2.66	-2.15	-99.1828	Tyr 116 B	2.93	-2.50
		Arg 472 B	3.13	-1.42			2.78	-1.74
		Glu 466 B	3.33	-0.72		Tyr 103 B	2.79	-2.50
**45**	-101.9210	Ser 464 B	2.56	-2.20	-96.7045	-		
		Thr 463 B	3.96	-0.70				
**46**	-100.4100	Ser 464 B	2.55	-2.07	-106.116	-		
		Lys 62 A	3.20	-1.93				
**47**	-102.4440	His 461 B	2.60	-1.56	-100.698	Tyr 103 B	2.65	-2.5
**48**	-84.4483	Asn 340 A	2.85	-2.50	-92.4174	Tyr 116 B	3.11	-2.47
		Thr 457 B	2.72	-2.50		Tyr 103 B	2.70	-2.50
		Ser 470 B	3.21	-0.98			2.48	-1.81
**49**	-91.8201	Ser 464 B	2.78	-2.50	-94.2563	-		
**50**	-93.6531	Thr 463 B	3.26	-1.71	-84.7217	Tyr 103 B	3.12	-2.42
**51**	-93.4097	Ser 464 B	2.50	-1.70	-96.9773	Tyr 103 B	3.05	-0.92

**52**	-99.7175	Ser 464 B<br>Lys 62 A	2.68<br>3.19	-2.50<br>-1.35	-96.1798	-		
**53**	-89.8147	Lys 62A<br>Ser 464 B	3.02<br>2.56	-2.07<br>-2.2	-86.3019	Tyr 103 B	2.83	-2.5
**54**	-91.1998	Lys 62A<br>Ser 464 B	3<br>2.63	-2.31<br>-2.5	-84.2155	Tyr 103 B	2.98	-2.5
**55**	-90.4967	Asn 340	2.97<br>3.02	2.5<br>-2.5	-89.4427	Tyr 103 B	3.03	-2.5
**56**	-92.4062	Asn 340 A<br>Gly 459 B	2.98<br>2.82	-2.5<br>2	-95.4859	Tyr 103 B	2.69	-2.34
**57**	-79.6845	Ser 464 B	3.11	-2.40	-80.471	Tyr 103 B	2.85<br>2.99	-4.02
**58**	-81.9298	Arg 355 A<br>Asn 340 A	2.45<br>2.86<br>3.18	-0.97<br>-0.90<br>-2.08	-82.6084	Tyr 103 B	2.75<br>3.13	-2.46<br>-2.13
**59**	-90.5529	Lys 62 A<br>Ser 464 B	3.05<br>2.55	-2.03<br>-2.12	-88.3182	Tyr 103 B	2.84<br>3.08	-2.5<br>-2.5
**60**	-91.8292	Lys 62 A<br>Ser 464 B	2.96<br>2.65	-2.21<br>-2.50	-90.6668	Tyr 103 B	2.75<br>3.16	-2.5<br>-1.77
**61**	-91.2961	Asn 340A	3.14<br>2.84	-0.81<br>-2.50	-90.6484	Tyr 103 B	2.9	-2.5
**62**	-94.5097	Asn 340A	2.98<br>3.16	-0.83<br>-2.21	-92.0233	Tyr 103 B	3.04	-2.4
**63**	-120.0300	Ser 15 A	3.01	2.20	-112.45	HEM_1450 B<br>Tyr 103 B	0<br>3.05<br>3.08<br>2.58	-2.06<br>-0.71<br>-2.5<br>-1.39
**64**	-128.7530	Asn 340 A<br>Gly 459 B<br>Arg 355 A	2.59<br>2.64<br>3.02	-2.41<br>-2.50<br>-2.06	-116.652	HEM_1450 B<br>Tyr 103 B	0<br>2.77<br>3.12	-2.21<br>-1.46<br>-2.41
**65**	-122.4240	Ser 15 A	3.08	-2.50	-114.785	HEM_1450 B	0	-2.5
**66**	-124.3250	Ser 15 A	3.02	-2.08	-121.598	Tyr 103 B	3.09<br>3.10<br>3.06	-1.79<br>-2.50<br>-2.50
**67**	-123.3630	Ser 15 A	3.10	-2.50	-107.714	-		
**68**	-123.5650	Ser 15 A	3.11	-2.45	-121.185	HEM_1450 B	0	-2.5

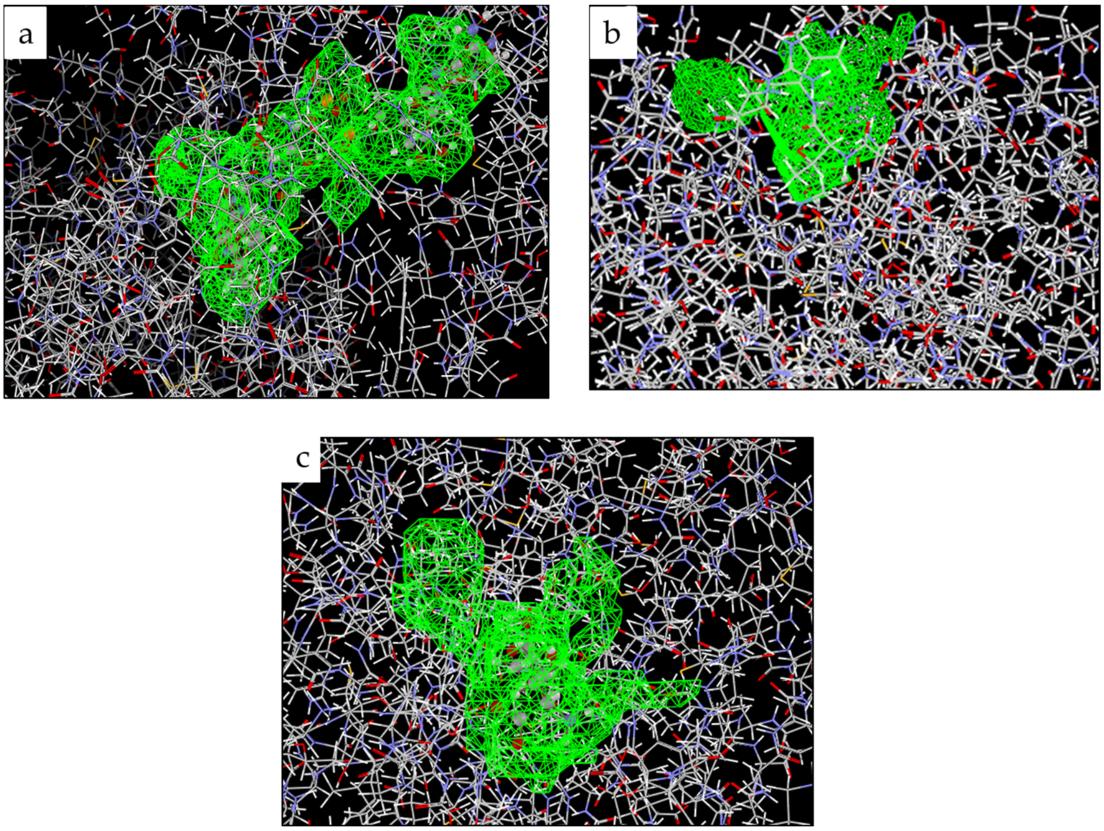

Figure S2. Cavities in the active site of *L*TR (**a**). *La*A (**b**). and *Li*TA (**c**) proteins.

Table S18. Results of the docking of naphthoquinone derivatives in the active site of the *L*TR protein.

ID	Energy (kcal/mol)	Hydrogen bond (HB)			ID	Energy (kcal/mol)	Hydrogen bond (HB)		
		Residues	Distance (Å)	Force			Residues	Distance (Å)	Force
**1**	-99.538	Lys 60	3.13	-2.16	**35**	-99.739	Arg 287	3.58	-0.10
**2**	-114.200	Ser 14	2.88	-2.50	**36**	-105.775	Lys 60	3.55	-0.21
		Thr 335	3.13	-2.00					
			3.11	-2.45					
			3.05	-2.50					
			2.32	-0.15					
		Arg 287	3.05	-2.04					
		Ser 162	3.18	-2.12					
**3**	-120.227	Cys 52	3.16	-2.18	**37**	-101.309	Ser 14	2.71	-2.50
		Ser 14	3.02	-2.50			Thr 335	3.19	-2.07
								2.57	-1.82
		Thr 335	3.56	-0.13			Asp 327	3.03	-0.17
							Arg 287	3.12	-2.31
		Lys 60	2.97	-1.77			Ser 162	2.93	-2.23
								3.37	-1.14
**4**	-116.010	Thr 335	3.55	-0.24	**38**	-100.132	Lys 60	2.75	-0.54
		Lys 60	2.98	-1.77					
**5**	-121.668	Thr 335	3.07	-2.50	**39**	-104.915	Lys 60	3.42	-0.64
			2.95	-2.04					
			2.99	-2.50					
		Ser 14	2.84	-2.50					
		Arg 287	3.05	-2.04					
		Ser 262	3.17	-1.77					
**6**	-127.514	Cys 52	3.18	-2.09	**40**	-106.550	Thr 335	2.75	-1.64
		Ser 14	3.04	-2.50				3.05	-2.50
								3.50	-0.49
		Thr 335	3.59	-0.05			Ser 14	2.97	-2.50
							Arg 287	3.04	-2.45
		Lys 60	2.98	-1.79			Ser 162	3.28	-1.62
								2.94	-2.39
**7**	-118.471	Thr 335	3.53	-0.36	**41**	-117.009	Thr 51	3.27	-0.78
								3.02	-0.17
		Lys 60	2.97	-1.76			Cys 52	3.41	-0.95
								2.89	-2.13
							Ser 14	3.43	-0.85
								3.50	-0.25
								2.63	-2.50

							Asp 327	3.53	-0.02
**8**	-100.768	Lys 60	3.10	-1.85	**42**	-114.535	Ser 14	3.04	-1.51
							Gly 15	2.92	-2.50
							Thr 335	3.35	-0.22
								3.07	-1.82
								3.08	-2.50
							Asp 327	2.85	-2.08
**9**	-107.033	Lys 60	2.65	-0.84	**43**	-101.908	Thr 335	3.00	-2.50
								3.06	-0.63
							Ser 162	2.54	-0.94
								3.50	-0.15
							Arg 287	3.30	-0.62
								3.40	-0.42
								2.71	-1.96
								3.15	-0.05
							Ser 14	3.03	-2.11
								2.80	-2.50
							Lys 60	2.78	-1.75
**10**	-114.045	Lys 60	3.08	-1.99	**44**	-112.801	Arg 287	3.02	-2.50
**11**	-106.527	Lys 60	2.51	-0.61	**45**	-93.208	Thr 335	2.85	-2.50
							Arg 287	3.10	-2.48
							Asp 327	2.98	-2.50
							Ser 162	2.97	-2.50
								2.89	-2.50
								2.75	-2.50
**12**	-112.686	Lys 60	3.06	-1.86	**46**	-109.701	Ser 14	2.73	-2.50
							Ser 162	3.10	-2.13
								3.58	-0.09
							Arg 287	3.05	-2.15
							Thr 335	2.95	-2.50
								2.77	-2.50
**13**	-108.036	Lys 60	2.62	-0.87	**47**	-114.397	Ser 14	2.72	-2.50
							Ser 162	3.16	-2.21
								3.22	-1.89
							Arg 287	3.11	-2.31
							Thr 335	3.32	-1.38
								3.08	-2.29
**14**	-111.703	Lys 60	3.07	-0.69	**48**	-93.423	Lys 60	2.85	-1.49
			2.64	-0.98					
**15**	-121.911	Ser 14	3.28	-1.45	**49**	-106.004	Lys 60	3.43	-0.62

			3.06	-2.50					
		Thr 51	3.03	-0.59					
		Cys 52	2.84	-2.50					
		Lys 60	3.37	-1.04					
**16**	-116.118	Lys 60	2.75	-1.72	**50**	-107.717	Arg 287	2.97	-2.50
		Thr 51	2.86	-2.50					
		Thr 335	2.85	-1.46					
			3.04	2.50					
**17**	-127.261	Ser 14	3.21	-1.93	**51**	-109.342	Lys 60	3.60	-0.01
		Thr 335	2.92	-2.50			Arg 287	3.14	-2.31
			2.96	-1.77					
			3.59	-0.07					
		Lys 60	2.62	-0.99					
		Ser 162	3.02	-2.31					
			3.42	-0.92					
		Arg 287	2.90	-2.25					
**18**	-96.817	Lys 60	2.61	-2.50	**52**	-109.544	Arg 287	2.91	-2.50
**19**	-113.030	Lys 60	3.06	-2.50	**54**	-114.265	Thr 51	2.74	-2.40
								3.18	-1.22
							Thr 335	2.93	-2.50
								3.08	-1.56
							Cys 52	3.07	-0.90
**20**	-114.556	Lys 60	3.03	-2.50	**54**	-118.580	Thr 51	2.60	-1.74
								3.30	-1.51
							Cys 52	3.55	-0.24
							Cys 57	3.00	-0.64
							Thr 335	3.13	-2.36
**21**	-104.131	Lys 60	2.69	-2.50	**55**	-118.004	Lys 60	2.98	-1.25
							Try 198	3.49	-0.53
							Thr 335	2.77	-1.76
								3.04	-2.50
**22**	-111.561	Lys 60	3.16	-2.19	**56**	-125.794	Thr 51	3.40	-0.57
								2.69	-1.91
							Cys 52	3.14	-0.98
							Thr 335	3.12	-2.42
							Cys 57	3.00	-0.64
**23**	-118.762	Lys 60	3.02	-1.85	**57**	-102.122	Lys 60	2.69	-1.36
								3.10	-2.08
**24**	-118.061	Ser 14	2.97	-2.50	**58**	-102.453	Lys 60	2.90	-1.53
		Thr 335	2.21	0.76					

			3.18	-2.11					
		Ser 162	3.17	-1.77				2.80	-1.98
		Arg 287	3.05	-2.04					
**25**	-116.771	Lys 60	3.57	-0.13	**59**	-105.655	Lys 60	2.36	-0.47
							Try 198	3.46	-0.39
**26**	-118.707	Lys 60	3.08	-2.00	**60**	-107.908	Lys 60	2.79	-1.82
								2.65	-1.77
**27**	-94.284	Lys 60	2.71	-0.51	**61**	-112.204	Thr 335	3.07	-2.50
								2.96	-1.03
							Lys 60	2.37	-0.44
							Tyr 198	3.57	-0.16
**28**	-107.867	Ser 14	2.57	-2.23	**62**	-121.668	Thr 335	3.15	-2.25
		Thr 335	2.57	-1.89			Thr 51	3.19	1.07
			3.11	-1.90				2.63	-1.85
			2.97	-2.50					
		Arg 287	3.07	-2.16			Cys 52	3.24	-0.71
		Ser 162	3.60	0					
			3.08	-2.08					
**29**	-113.613	Ser 14	2.88	-2.50	**63**	-145.502	Lys 60	2.80	-2.06
		Cys 52	3.16	-2.19			Cys 52	3.10	-1.07
								3.34	-1.18
		Lys 60	3.51	-0.44			Thr 335	3.50	-0.19
**30**	-108.388	Ser 14	2.57	-2.27	**64**	-155.221	Lys 60	2.53	-1.26
		Tyr 198	3.45	-0.76			Cys 52	3.09	-0.83
		Lys 60	3.37	-0.49				3.23	-1.53
		Thr 335	2.57	-1.87			Thr 335	3.24	-0.54
			2.97	-2.50					
		Arg 287	3.05	-2.13			Ser 14	3.54	-0.30
		Ser 162	3.11	-2.07					
**31**	-115.255	Ser 14	2.57	-2.22	**65**	-158.024	Lys 60	3.02	-2.26
		Thr 335	2.57	-1.88			Cys 52	3.08	-2.37
			3.09	-1.91				3.03	-1.04
			2.97	-2.50			Thr 335	3.14	-0.69
		Arg 287	3.07	-2.15					
		Ser 162	3.09	-2.09			Ser 14	3.35	-1.27
**32**	-115.011	Ser 14	3.45	-0.73	**66**	-147.809	Lys 60	2.18	-1.93
		Cys 52	3.08	-2.50			Cys 52	3.11	-1.13
								3.39	-1.03
							Thr 335	3.19	-0.72
							Ser 14	3.37	-1.15
**33**	-118.296	Ser 14	2.56	-2.19	**67**	-143.973	Lys 60	3.51	-0.33
		Thr 198	3.22	-1.90			Thr 335	3.07	-1.09

		Thr 335	3.10	-2.50				3.44	-0.11
			3.18	-2.09				3.07	-0.14
		Arg 287	3.19	-2.06			Cys 52	2.98	-0.81
		Ser 162	3.11	-2.44					
			3.09	-2.50					
**34**	-95.239	Thr 335	3.04	-0.34	**68**	-151.683	Lys 60	2.59	-1.68
			2.96	-1.89					
			3.01	-0.99			Cys 52	3.38	-0.42

Table S19. Results of the docking of naphthoquinone derivatives in the active site of the *La*A protein.

ID	Energy (kcal/mol)	Hydrogen Bond (HB)			ID	Energy (kcal/mol)	Hydrogen Bond (HB)		
		Residues	Distance (Å)	Force			Residues	Distance (Å)	Force
**1**	-81.600	Val 149	3.10	-2.42	**35**	-90.191	Val 149	2.74	-1.80
							Ser 150	3.10	-1.06
		Thr 148	2.98	-2.50			Asn 152	3.26	-1.53
		Ser 150	3.48	-0.12			Thr 148	2.60	-2.49
								3.10	-2.49
**2**	-83.661	-	-	-	**36**	-89.049	Val 149	3.18	-1.63
							Asn 152	3.12	-1.76
							Thr 148	2.60	-2.50
								3.39	-0.96
**3**	-90.616	-	-	-	**37**	-82.695	Asp 194	2.63	-2.50
**4**	-99.235	Thr 257	3.00	-2.50	**38**	-88.696	Asp 194	2.60	-1.65
		Thr 148	2.60	-2.21			Glu 197	3.15	-1.61
		Val 149	3.21	-1.12			Asp 141	3.04	-1.88
		Ser 150	2.94	-2.46					
**5**	-100.881	His 154	3.11	-1.85	**39**	-82.015	Ala 140	3.45	-0.30
							Asp 141	3.20	-2.01
							Ala 192	2.60	-0.66
							Glu 197	2.58	-0.66
							Asp 194	2.72	-2.50
**6**	-102.105	-	-	-	**40**	-105.329	Ser 150	3.27	-1.66
								3.48	-0.60
								2.82	-1.36
								2.60	-2.50
								2.60	-2.43
							Thr 148	3.43	-0.85
								2.41	-0.84
							Asn 143	3.32	-0.03
								2.87	-0.10
							Val 149	2.89	-2.50
							Asp 194	2.60	-1.25
							Asp 141	3.33	-0.99
**7**	-100.978	Thr 148	3.14	-2.29	**41**	-108.543	Val 149	3.08	-2.39
								2.60	-2.50
		Asn 143	2.60	-2.50			Ser 150	3.36	-1.20
								3.29	-1.53
								2.60	-2.50
		Ser 150	2.56	-1.97			Thr 148	3.27	-1.67
								2.34	-0.30
							Glu 197	3.10	-1.67
			3.27	-0.25			Asp 194	2.65	-0.24

							Asp 141	3.07	-1.72
**8**	-83.514	Ser 150	3.10	-2.50	**42**	-94.784	Thr 257	3.32	-1.38
			2.58	-1.67			Ser 150	3.00	-0.68
		Val 149	3.39	-0.77			Thr 148	2.77	-2.50
								3.27	-0.85
							Asn 152	3.35	-0.70
		Thr 148	3.12	-2.40			Val 149	3.10	-2.18
**9**	-89.104	Thr 148	3.01	-2.50	**43**	-111.305	Ser 150	2.96	-2.21
								2.60	-2.30
								2.60	-2.50
								3.29	-1.57
								3.47	-0.65
		Asn 143	2.61	-2.49			Gly 151	3.55	-0.22
							Thr 148	3.34	-1.29
								2.31	-0.10
							Val 149	2.87	-2.50
							Asp 194	2.91	-1.10
		Val 149	3.22	-1.81			Asp 141	3.10	-1.60
**10**	-85.563	Ser 150	2.86	-1.68	**44**	-75.88	Ser 150	3.39	-1.07
		Asp 141	3.14	-1.47			Val 149	2.87	-2.5
							Asn 143	3.1	-2.5
								3.1	-2.5
								3.2	-1.98
**11**	-81.801	-	-	-	**45**	-86.191	Asn 143	2.86	-2.01
**12**	-85.155	Asn 143	2.86	-0.50	**46**	-81.159	Asn 152	3.19	-0.92
		Thr 148	2.91	-2.50					
**13**	-89.131	Thr 148	2.90	-2.33	**47**	-85.875	Thr 148	3.36	-1.22
							Asn 143	2.7	-1.08
							Thr 257	3.04	-2.5
**14**	-101.370	Ser 150	2.95	-1.51	**48**	-94.363	Val 149	2.63	-2.50
			3.30	-1.48					
			3.38	-1.12			Ser 150	3.03	-1.00
			2.62	-2.26					
			2.51	-1.79					
		Thr 148	3.30	-1.51			Thr 148	3.11	-2.46
			2.31	-0.08					
		Gly 151	3.52	-0.34				3.10	-2.09
		Val 149	2.96	-2.44					
		Asp 141	3.11	-1.58					

**15**	-91.161	Thr 257	3.10	-2.50	**49**	-86.276	Asp 141	3.10	-2.49
							Ala 140	3.48	-0.22
							Glu 197	2.44	-0.59
							Asp 194	2.65	-0.33
**16**	-91.305	Asn 143	2.60	-0.34	**50**	-96.301	Asp 194	3.56	-0.001
		Thr 148	3.08	-2.50				3.58	-0.01
		Thr 257	2.90	-2.50				2.60	-2.50
		Ser 150	2.46	-1.09			Ala 192	3.10	-2.50
**17**	-105.910	Asn 143	3.20	-0.23	**51**	-90.319	His 139	3.14	-2.29
			3.11	-1.20					
		Ser 150	3.02	-2.50					
		His 139	2.73	-0.02					
		Thr 257	3.10	-2.39					
**18**	-85.587	Thr 143	3.06	-2.50	**52**	-96.058	Thr 148	2.97	-2.50
								3.11	-2.06
		Val 149	3.09	-2.50			Asn 152	3.53	-0.34
		Ser 150	2.60	-2.47			Val 149	2.97	-2.48
							Ser 150	2.60	-2.50
							Asn 143	2.68	-2.50
**19**	-85.055	-	-	-	**53**	-83.897	Asp 141	3.05	-1.82
							Glu 197	3.13	-0.47
							Asp 194	2.6	-1.41
**20**	-82.806	-	-	-	**54**	-89.613	Thr 148	2.6	-2.06
							Asn 152	2.78	-1.58
							Ser 150	3.35	-0.08
**21**	-84.880	-	-	-	**55**	-87.163	Thr 148	2.6	-2.5
							Asn 143	2.6	-0.16
							Asn 152	2.86	-0.03
							His 154	3.5	-0.19
**22**	-92.461	-	-	-	**56**	-94.045	Thr 148	2.98	-2.1
							His 154	3.46	-0.71
							Asn 152	3.2	-2.01
**23**	-84.315	Asn 143	3.10	-2.44	**57**	-67.630	Ser 150	3.13	-2.27
		Ser 150	2.41	-0.04			Asn 143	3.35	-0.57
**24**	-97.226	Asn 143	3.22	-1.58	**58**	-86.668	Asn 143	2.94	-1.58
**25**	-89.138	-	-	-	**59**	-71.623	Asn 152	3.24	-1.42
							Ser 150	3.25	-0.53
								2.82	-2.50
							Thr 257	3.46	-0.67
**26**	-99.623	Ser 150	3.12	-2.38	**60**	-76.008	Asn 143	2.88	-0.54
			2.41	-0.71					
		Val 149	3.47	-0.42					
		Thr 148	2.94	-2.29					
**27**	-85.381	Asp 141	3.09	-2.03	**61**	-71.498	Val 149	2.64	-2.40
		Glu 197	3.36	-1.03			Thr 148	3.34	-1.29

							Asn 143	2.71	-0.07
							Ser 150	3.20	-2.02
								3.44	-0.35
**28**	-87.716	Asp 141	3.07	-1.72	**62**	-83.099	Thr 148	3.47	-0.63
		Glu 197	3.14	-0.92					
		Asp 194	2.60	-2.42			Asn 143	3.10	-0.84
**29**	-99.351	Val 149	3.00	-1.95	**63**	-104.823	Asn 143	3.10	-0.81
								3.33	-0.11
		Thr 148	2.74	-2.50			Ser 150	3.11	-2.21
								3.20	-2.01
		Ser 150	3.29	-1.53				3.01	-2.50
								2.90	-1.68
							Thr 148	2.33	-0.14
			3.29	-2.50				2.62	-2.50
		Asp 141	3.05	-1.74			Val 149	2.95	-1.80
		Glu 197	3.07	-1.19					
		Asp 194	2.65	-2.49					
**30**	-88.975	Asn 143	2.60	-0.23	**64**	-116.708	Ser 150	2.60	-1.12
								3.24	-1.63
								3.39	-1.03
								2.86	-2.50
		Thr 148	3.00	-2.50			Asn 143	3.39	-0.52
								3.14	-0.15
								3.05	-0.11
		Ser 150	2.40	-0.56			Thr 148	2.60	-2.50
								2.60	-1.41
							Val 149	3.28	-1.09
**31**	-90.099	Asp 194	2.90	-2.50	**65**	-110.876	Asn 152	3.18	-2.11
								3.33	-0.14
							Thr 148	2.79	-2.50
								2.09	1.81
							Asn 143	3.48	-0.07
		Ala 192	3.10	-2.48				3.10	-0.38
							Val 149	2.67	-2.02
								3.40	-0.54
							Ser 150	3.10	-2.50
								3.10	-2.50
								3.53	-0.36
**32**	-10.517	Thr 257	3.44	-0.80	**66**	-121.516	Asn 152	3.08	-2.50
								3.10	-0.43
		Ser 150	3.53	-0.01			Thr 148	1.99	2.74
								3.52	-0.38
								2.54	-1.95
		Thr 148	3.10	-2.50			Val 149	3.15	-0.15

								2.61	-1.18
								3.34	-1.31
			2.90	-1.66			Ser 150	2.80	-2.50
								3.10	-1.51
								2.16	1.26
								2.49	-1.57
		Val 149	2.60	-2.49			Gly 151	3.22	-1.32
**33**	-10.459	Val 149	2.63	-2.50	**67**	-115.454	Ser 150	3.36	-1.20
								3.04	-2.15
								3.10	-2.50
								2.81	-1.15
		Ser 150	2.88	-1.71			Thr 148	2.66	-2.50
								2.46	-0.80
		Thr 148	2.87	-1.22			Asn 143	3.32	-0.11
								3.16	-0.87
							Val 149	3.10	-1.85
**34**	-82.222	Thr 148	2.99	-2.17	**68**	-112.983	Val 149	2.65	-1.76
		His 154	3.29	-1.53			Thr 148	2.60	-2.50
		Asn 152	3.10	-2.50			Asn 152	3.42	-0.87
								2.50	-1.65
								2.97	-2.50
							Ser 150	3.01	-2.50
								2.89	-2.50
								3.52	-0.39

Table S20. Results of the docking of naphthoquinone derivatives in the active site of the *Li*TA protein.

ID	Energy (kcal/mol)	Hydrogen bond (HB)			ID	Energy (kcal/mol)	Hydrogen bond (HB)		
		Residues	Distance (Å)	Force			Residues	Distance (Å)	Force
			2.96	-2.50			Tyr 256	2.18	1.05
**1**	-79.606	Tyr 256	2.79	-0.74	**35**	-82.252	Asn 225	3.10	-2.29
		Asn 225	2.90	-1.44				3.01	-2.50
**2**	-85.849	Gly 79	3.46	-0.39	**36**	-80.826	Tyr 256	2.95	-2.50
								2.93	-2.50
**3**	-86.939	Tyr 256	3.00	-1.42	**37**	-82.337	Tyr 256	2.86	-2.50
			3.03	-2.50				2.73	-2.50
**4**	-87.891	Tyr 256	3.02	-2.50	**38**	-81.804	Tyr 256	2.94	-2.50
			2.74	-2.50				3.01	-2.50
**5**	-92.143	Tyr 256	3.08	-2.50	**39**	-83.067	Tyr 256	2.94	-2.50
								2.60	-2.50
								3.30	-1.48
							Tyr 256	3.12	-2.40
								3.36	-1.19
**6**	-95.046	-	-	-	**40**	-84.225	Asn 225	2.43	-1.12
			3.10	-2.50			Tyr 256	2.67	-2.50
								3.50	-0.24
**7**	-90.656	Tyr 256	2.94	-2.50	**41**	-79.365	Asn 225	3.31	-0.61
			2.99	-2.50					
**8**	-84.381	Tyr 256	2.89	-2.50	**42**	-96.985	Asn 225	3.10	-1.32
		Tyr 256	3.10	-2.34					
							Asn 225	2.60	-2.49
								3.02	-0.05
							Gly 79	3.16	-2.19
								2.60	-2.50
**9**	-89.649	Asn 225	2.19	0.93	**43**	-74.541	Tyr 256	3.02	-0.93
			2.92	-2.50					
**10**	-84.295	Tyr 256	3.07	-2.50	**44**	-83.134	Tyr 256	3.1	-2.5
			2.95	-2.50				2.95	-2.5
**11**	-84.611	Tyr 256	2.87	-2.50	**45**	-78.98	Tyr 256	2.74	-2.5
			2.85	-2.50					
**12**	-83.999	Tyr 256	2.96	-2.50	**46**	-81.058	Asn 256	2.96	-1.43
**13**	-83.373	Asn 225	3.01	-1.50	**47**	-88.304	-	-	-
			2.95	-2.50					
**14**	-87.445	Tyr 256	2.84	-2.50	**48**	-82.930	Tyr 256	2.99	-2.49
			2.93	-2.50					
**15**	-94.888	Tyr 256	2.79	-2.50	**49**	-84.899	Asn 225	3.00	-1.43
			3.03	-2.50				2.75	-0.69
**16**	-88.165	Tyr 256	2.99	-2.50	**50**	-94.022	Gly 79	2.63	-0.57
		Asn 225	3.14	-1.27					
**17**	-94.674	Gly 79	3.43	-0.50	**51**	-87.735	Tyr 256	2.71	-1.57

			3.07	-2.50				2.98	-2.50
**18**	-80.138	Tyr 256	2.91	-1.79	**52**	-66.356	Tyr 256	3.02	-2.50
								3.1	-0.88
			2.94	-2.50		-74.986	Tyr 256	2.86	-2.5
**19**	-87.227	Tyr 256	2.78	-2.50				2.89	-0.31
								3.1	-0.29
			3.03	-2.50		-77.535	Tyr 256	2.69	-2.5
**20**	-85.218	Tyr 256	2.98	-2.50		-81.348	Asn 225	3.12	-2.42
								2.69	-0.72
			3.04	-2.50			Tyr 256	2.6	-2.5
**21**	-91.061	Tyr 256	2.77	-0.92				3.08	-2.5
			2.94	-2.50		-66.914	Tyr 256	2.65	-1.19
**22**	-89.106	Tyr 256	2.79	-1.01				3.12	-2.41
		Asn 225	3.10	-1.40					
**23**	-85.104	Gly 79	3.43	-0.43	**57**	-66.913	-	-	-
								3.16	-2.18
**24**	-83.580	Tyr 256	3.11	-2.44	**58**	-69.657	Tyr 256	2.75	-1.29
								2.87	-2.50
**25**	-96.427	Asn 225	3.06	-1.87	**59**	-70.068	Tyr 256	3.09	-0.04
								3.15	-2.25
**26**	-87.100	-	-	-	**60**	-76.338	Tyr 256	2.80	-1.20
								2.89	-2.50
**27**	-86.191	-	-	-	**61**	-72.644	Tyr 256	3.07	-0.13
			2.92	-2.50					
**28**	-85.924	Tyr 256	2.88	-2.50	**62**	-78.314	Tyr 256	3.08	-2.50
							Asn 225	3.12	-1.78
								3.44	-0.78
								3.28	-1.58
**29**	-84.210	Tyr 256	2.74	-0.14	**63**	-109.969	Tyr 256	2.97	-2.50
			3.00	-2.50				3.07	-2.50
**30**	-89.359	Tyr 256	3.01	-2.50	**64**	-119.504	Tyr 256	2.98	-2.45
			2.77	-2.50			Gly 79	2.71	-2.71
								2.84	-1.70
								2.68	-2.50
**31**	-95.517	Tyr 256	3.10	-2.50	**65**	-108.703	Tyr 256	3.10	-2.49
			2.96	-2.50				3.28	-1.60
								2.70	-2.50
							Tyr 256	3.14	-2.21
**32**	-87.559	Tyr 256	2.73	-2.50	**66**	-108.761	Asn 225	3.01	-1.66
			3.02	-2.50				3.08	-2.50
**33**	-80.096	Tyr 256	3.01	-2.50	**67**	-121.426	Tyr 256	3.00	-2.46
			2.98	-2.50			Asn 225	3.12	-1.65
								3.23	-1.85
								2.87	-2.50
**34**	-87.434	Try 256	2.82	-2.50	**68**	-113.611	Tyr 256	3.17	-1.91

Table S21. ADME parameters

ID	MW	H-bond acceptors	H-bond donors	TPSA	Log P	Solubility (Log S)			Pharmacokinetics								Lipinski	Ghose	Veber	Egan	Muegge	Bio-availability Score
									GI	Pgp	CYP					log Kp (cm/s)						
						ESOL	Ali	Silicos-IT			1A2	2C19	2C9	2D6	3A4							
1	283.71	2	1	46.17	3.06	-4.57	-4.9	-6.43	High	No	Yes	Yes	Yes	Yes	Yes	-5.03	0	0	0	0	0	0.55
2	313.74	3	1	55.4	3.07	-4.63	-5.06	-6.55	High	No	Yes	Yes	Yes	Yes	Yes	-5.24	0	0	0	0	0	0.55
3	313.74	3	1	55.4	3.05	-4.63	-5.06	-6.55	High	No	Yes	Yes	Yes	Yes	Yes	-5.24	0	0	0	0	0	0.55
4	313.74	3	1	55.4	3.05	-4.63	-5.06	-6.55	High	No	Yes	Yes	Yes	Yes	Yes	-5.24	0	0	0	0	0	0.55
5	327.76	3	1	55.4	3.4	-4.87	-5.45	-6.95	High	No	Yes	Yes	Yes	Yes	Yes	-5.06	0	0	0	0	0	0.55
6	326.77	3	0	43.37	3.9	-4.86	-5.19	-7.3	High	No	Yes	Yes	Yes	Yes	Yes	-5.06	0	0	0	0	0	0.55
7	327.76	3	1	55.4	3.38	-4.87	-5.45	-6.95	High	No	Yes	Yes	Yes	Yes	Yes	-5.06	0	0	0	0	0	0.55
8	301.7	3	1	46.17	3.41	-4.72	-5	-6.71	High	No	Yes	Yes	Yes	Yes	Yes	-5.07	0	0	0	0	0	0.55
9	301.7	3	1	46.17	3.38	-4.72	-5	-6.71	High	No	Yes	Yes	Yes	Yes	Yes	-5.07	0	0	0	0	0	0.55
10	301.7	3	1	46.17	3.38	-4.72	-5	-6.71	High	No	Yes	Yes	Yes	Yes	Yes	-5.07	0	0	0	0	0	0.55
11	318.15	2	1	46.17	3.62	-5.16	-5.55	-7.04	High	No	Yes	Yes	Yes	No	Yes	-4.8	0	0	0	0	0	0.55
12	318.15	2	1	46.17	3.62	-5.16	-5.55	-7.04	High	No	Yes	Yes	Yes	No	Yes	-4.8	0	0	0	0	0	0.55
13	318.15	2	1	46.17	3.6	-5.16	-5.55	-7.04	High	No	Yes	Yes	Yes	No	Yes	-4.8	0	0	0	0	0	0.55
14	328.71	4	1	91.99	2.47	-4.96	-6.26	-5.79	High	No	Yes	Yes	Yes	No	Yes	-5.04	0	0	0	0	0	0.55
15	328.71	4	1	91.99	2.3	-4.62	-5.69	-5.79	High	No	Yes	Yes	Yes	No	Yes	-5.43	0	0	0	0	0	0.55
16	328.71	4	1	91.99	2.3	-4.62	-5.69	-5.79	High	No	Yes	Yes	Yes	No	Yes	-5.43	0	0	0	0	0	0.55
17	373.7	6	1	137.81	1.68	-5.03	-7.04	-5.14	Low	No	Yes	Yes	Yes	Yes	Yes	-5.43	0	0	0	1	0	0.55
18	297.74	2	1	46.17	3.41	-4.86	-5.27	-6.82	High	No	Yes	Yes	Yes	Yes	Yes	-4.86	0	0	0	0	0	0.55
19	297.74	2	1	46.17	3.42	-4.86	-5.27	-6.82	High	No	Yes	Yes	Yes	Yes	Yes	-4.86	0	0	0	0	0	0.55
20	297.74	2	1	46.17	3.4	-4.86	-5.27	-6.82	High	No	Yes	Yes	Yes	Yes	Yes	-4.86	0	0	0	0	0	0.55
21	311.76	2	1	46.17	3.7	-5.14	-5.73	-7.21	High	No	Yes	Yes	Yes	Yes	Yes	-4.64	0	0	0	0	1	0.55
22	311.76	2	1	46.17	3.7	-5.14	-5.73	-7.21	High	No	Yes	Yes	Yes	Yes	Yes	-4.64	0	0	0	0	1	0.55
23	311.76	2	1	46.17	3.7	-5.14	-5.73	-7.21	High	No	Yes	Yes	Yes	Yes	Yes	-4.64	0	0	0	0	1	0.55
24	325.79	2	1	46.17	4.01	-5.42	-6.07	-7.24	High	No	Yes	Yes	Yes	No	Yes	-4.49	0	0	0	0	1	0.55
25	325.79	2	1	46.17	4.02	-5.42	-6.07	-7.24	High	No	Yes	Yes	Yes	No	Yes	-4.49	0	0	0	0	1	0.55

26	325.79	2	1	46.17	4.01	-5.42	-6.07	-7.24	High	No	Yes	Yes	Yes	No	Yes	-4.49	0	0	0	0	1	0.55
27	264.28	2	2	72.19	2.05	-3.62	-4.09	-5.47	High	No	Yes	Yes	Yes	No	Yes	-5.85	0	0	0	0	0	0.55
28	294.3	3	2	81.42	1.95	-3.57	-4.06	-5.58	High	No	Yes	Yes	Yes	No	Yes	-6.18	0	0	0	0	0	0.55
29	294.3	3	2	81.42	1.94	-3.68	-4.25	-5.58	High	No	Yes	Yes	Yes	No	Yes	-6.05	0	0	0	0	0	0.55
30	294.3	3	2	81.42	1.95	-3.68	-4.25	-5.58	High	No	Yes	Yes	Yes	No	Yes	-6.05	0	0	0	0	0	0.55
31	308.33	3	2	81.42	2.26	-3.81	-4.45	-5.98	High	No	Yes	Yes	Yes	Yes	Yes	-6	0	0	0	0	0	0.55
32	308.33	3	2	81.42	2.23	-3.92	-4.63	-5.98	High	No	Yes	Yes	Yes	Yes	Yes	-5.87	0	0	0	0	0	0.55
33	308.33	3	2	81.42	2.27	-3.92	-4.63	-5.98	High	No	Yes	Yes	Yes	Yes	Yes	-5.87	0	0	0	0	0	0.55
34	282.27	3	2	72.19	2.28	-3.78	-4.19	-5.74	High	No	Yes	No	No	Yes	Yes	-5.88	0	0	0	0	0	0.55
35	282.27	3	2	72.19	2.26	-3.78	-4.19	-5.74	High	No	Yes	No	No	Yes	Yes	-5.88	0	0	0	0	0	0.55
36	282.27	3	2	72.19	2.25	-3.78	-4.19	-5.74	High	No	Yes	No	No	Yes	Yes	-5.88	0	0	0	0	0	0.55
37	298.72	2	2	72.19	2.49	-4.21	-4.74	-6.07	High	No	Yes	Yes	Yes	Yes	Yes	-5.61	0	0	0	0	0	0.55
38	298.72	2	2	72.19	2.49	-4.21	-4.74	-6.07	High	No	Yes	Yes	Yes	Yes	Yes	-5.61	0	0	0	0	0	0.55
39	298.72	2	2	72.19	2.48	-4.21	-4.74	-6.07	High	No	Yes	Yes	Yes	Yes	Yes	-5.61	0	0	0	0	0	0.55
40	309.28	4	2	118.01	1.27	-3.9	-5.26	-4.83	High	No	Yes	No	Yes	No	Yes	-5.98	0	0	0	0	0	0.55
41	309.28	4	2	118.01	1.22	-3.67	-4.87	-4.83	High	No	Yes	No	Yes	No	Yes	-6.24	0	0	0	0	0	0.55
42	309.28	4	2	118.01	1.2	-3.67	-4.87	-4.83	High	No	Yes	No	Yes	No	Yes	-6.24	0	0	0	0	0	0.55
43	354.27	6	2	163.83	0.49	-3.97	-6.04	-4.18	Low	No	No	No	Yes	No	Yes	-6.37	0	0	1	1	1	0.55
44	292.33	2	2	72.19	2.57	-4.2	-4.92	-6.25	High	No	Yes	Yes	Yes	Yes	Yes	-5.45	0	0	0	0	0	0.55
45	306.36	2	2	72.19	2.86	-4.36	-5.07	-6.27	High	No	Yes	Yes	Yes	Yes	Yes	-5.43	0	0	0	0	0	0.55
46	306.36	2	2	72.19	2.89	-4.47	-5.26	-6.27	High	No	Yes	Yes	Yes	Yes	Yes	-5.3	0	0	0	0	0	0.55
47	306.36	2	2	72.19	2.87	-4.47	-5.26	-6.27	High	No	Yes	Yes	Yes	Yes	Yes	-5.3	0	0	0	0	0	0.55
48	278.31	2	2	72.19	2.26	-3.8	-4.27	-5.85	High	No	Yes	Yes	Yes	Yes	Yes	-5.8	0	0	0	0	0	0.55
49	278.31	2	2	72.19	2.29	-3.92	-4.47	-5.85	High	No	Yes	Yes	Yes	Yes	Yes	-5.67	0	0	0	0	0	0.55
50	278.31	2	2	72.19	2.28	-3.92	-4.47	-5.85	High	No	Yes	Yes	Yes	Yes	Yes	-5.67	0	0	0	0	0	0.55
51	292.33	2	2	72.19	2.56	-4.08	-4.73	-6.25	High	No	Yes	Yes	Yes	Yes	Yes	-5.58	0	0	0	0	0	0.55
52	292.33	2	2	72.19	2.58	-4.2	-4.92	-6.25	High	No	Yes	Yes	Yes	Yes	Yes	-5.45	0	0	0	0	0	0.55
53	290.27	5	0	69.15	2.21	-3.87	-3.9	-6.29	High	No	Yes	No	Yes	Yes	Yes	-6.09	0	0	0	0	0	0.55
54	290.27	5	0	69.15	2.25	-3.87	-3.9	-6.29	High	No	Yes	No	Yes	Yes	Yes	-6.09	0	0	0	0	0	0.55
55	304.3	5	0	69.15	2.55	-4.1	-4.28	-6.69	High	No	Yes	No	Yes	No	Yes	-5.91	0	0	0	0	0	0.55
56	304.3	5	0	69.15	2.56	-4.1	-4.28	-6.69	High	No	Yes	No	Yes	No	Yes	-5.91	0	0	0	0	0	0.55
57	274.27	4	0	59.92	2.59	-4.11	-4.11	-6.56	High	No	Yes	No	No	No	Yes	-5.72	0	0	0	0	0	0.55
58	274.27	4	0	59.92	2.62	-4.11	-4.11	-6.56	High	No	Yes	No	Yes	No	Yes	-5.72	0	0	0	0	0	0.55
59	288.3	4	0	59.92	2.88	-4.38	-4.57	-6.96	High	No	Yes	Yes	Yes	No	Yes	-5.49	0	0	0	0	0	0.55

60	288.3	4	0	59.92	2.88	-4.38	-4.57	-6.96	High	No	Yes	Yes	Yes	No	Yes	-5.49	0	0	0	0	0	0.55
61	302.33	4	0	59.92	3.19	-4.65	-4.91	-6.99	High	No	Yes	Yes	Yes	No	Yes	-5.34	0	0	0	0	0	0.55
62	302.33	4	0	59.92	3.19	-4.65	-4.91	-6.99	High	No	Yes	Yes	Yes	No	Yes	-5.34	0	0	0	0	0	0.55
63	364.79	4	1	76.88	2.65	-4.49	-4.74	-6.95	High	No	Yes	Yes	Yes	No	Yes	-6.08	0	0	0	0	0	0.55
64	382.78	5	1	76.88	3.01	-4.65	-4.84	-7.22	High	No	Yes	Yes	Yes	No	Yes	-6.12	0	0	0	0	0	0.55
65	382.78	5	1	76.88	3.01	-4.65	-4.84	-7.22	High	No	Yes	Yes	Yes	No	Yes	-6.12	0	0	0	0	0	0.55
66	382.78	5	1	76.88	2.98	-4.65	-4.84	-7.22	High	No	Yes	Yes	Yes	No	Yes	-6.12	0	0	0	0	0	0.55
67	400.77	6	1	76.88	3.3	-4.8	-4.94	-7.48	High	No	Yes	Yes	Yes	No	Yes	-6.16	0	0	0	0	0	0.55
68	400.77	6	1	76.88	3.28	-4.8	-4.94	-7.48	High	No	Yes	Yes	Yes	No	Yes	-6.16	0	0	0	0	0	0.55
